# Community Case Study of Naloxone Distribution by Hospital-Based Harm Reduction Program for People Who Use Drugs in New York City

**DOI:** 10.3389/fsoc.2021.619683

**Published:** 2021-07-07

**Authors:** Farah Riazi, Wilma Toribio, Emaun Irani, Terence M. Hughes, Zina Huxley-Reicher, Elisa McBratney, Trang Vu, Keith Sigel, Jeffrey J. Weiss

**Affiliations:** ^1^ Division of General Internal Medicine, Department of Medicine, Icahn School of Medicine at Mount Sinai, New York, NY, United States; ^2^ Icahn School of Medicine at Mount Sinai, New York, NY, United States

**Keywords:** naloxone, narcan, overdose, opioid education, naloxone training, take-home-naloxone, overdose education and naloxone distribution, COVID-19

## Abstract

**Background:** In 2017, The Respectful and Equitable Access to Comprehensive Healthcare (REACH) Program at Mount Sinai Hospital became a registered Opioid Overdose Prevention Program (OOPP) and received funding from the New York City Department of Health and Mental Hygiene to develop a program to provide overdose education and naloxone distribution (OEND) training to at risk population and bystanders. We report on the programmatic quality improvement initiatives conducted.

**Methods:** From April 2017 to December 2020, the REACH OOPP conducted 290 opioid overdose reversal trainings, throughout the Mount Sinai Health System and in multiple other community settings. OEND training was at times offered alone and in other settings alongside Hepatitis C Virus point of care testing. Additionally, a “train the trainer” model was implemented whereby medical students and nurses at outpatient clinics were trained to train others.

**Results:** There were 4235 naloxone kits distributed to 3,906 participants. The training venues included hospital settings (patients and medical staff), public events, substance use programs, educational facilities, homeless prevention programs, faith-based organizations, alternative to incarceration programs, and community-based organizations. We implemented two types of training. During outreach sessions, we utilized one-on-one personalized sessions to train bystanders. When training clinic staff in the “train the trainer” model we utilized a standardized didactic presentation with slides. The two top reasons participants reported for being trained were “Just in case I see someone overdose” (59.3%) and “I'm worried that someone I know will overdose OR that I will overdose” (20.2%).

**Conclusion:** The REACH program at Mount Sinai Hospital developed an effective model to train community bystanders and health care staff by leveraging administrative support and building on broader programmatic initiatives to promote drug user health and stigma-free care for people who use drugs. Hospitals do not currently mandate staff training or keeping naloxone stocked at inpatient units or outpatients clinics posing a challenge when implementing an OEND program in this setting. A recommended policy change needed to decrease overdose deaths is for hospitals to be required to implement systematic naloxone education and access for all health care personal and at risk patients.

## Introduction

Unintentional drug overdose deaths related to opioids have been on the rise in the United States (U.S.) since 1999, becoming a public health concern that has affected a wide spectrum of Americans. The rate of drug overdose deaths involving heroin, a “natural” opioid derived from opium, increased by 18% per year from 2014 to 2016 (Hedegaard et al., 2017). Deaths involving synthetic opioids other than methadone, including fentanyl, a synthetic opioid 30 to 50 times stronger than heroin, increased by 88% per year from 2013 to 2016 (Hedegaard et al., 2017). The total cost of the opioid overdose epidemic in the U.S., including costs associated with the use and misuse of prescription and illicit opioids in 2015, has been estimated at over $500 billion (Haegerich et al., 2019; Council of Economic Advisers, 2020). Interventions that stem the mortality and cost related to opioid overdose are a major public health priority.

Naloxone is a competitive opioid antagonist that temporarily displaces opioids in the brain and reverses opioid-induced respiratory depression (Doyon et al., 2014). Naloxone is a safe, rapid, effective and easy-to-use (nasally administered) medication, without any psychoactive effects and no potential for abuse (Bazazi et al., 2010). The increased opioid overdoses have shifted naloxone from emergency rooms to first responders such as police, emergency medical technicians, and the friends and family of overdose victims (Skolnick 2018). Naloxone has played an essential role in community-based health promotion programs, proving an invaluable tool for laypersons who witness an overdose. Research has demonstrated that naloxone distribution to laypersons provided by community organizations may decrease opioid-related deaths in those communities (Wheeler et al., 2015; Opioid Overdose Prevention Programs Providing Naloxone to Laypersons — United States, 2014, 2020).

The U.S. Department of Health and Human Services (HHS) launched a comprehensive campaign called the “5-Point Strategy” to better combat the opioid crisis and naloxone distribution was one of key components (Division, 2018). In April 2018, the U.S. Surgeon General issued a public health advisory on naloxone and opioid overdose encouraging health care providers to play an active role in naloxone awareness, availability and administration (Sohn et al., 2019). In 2016, Public Health Law Section 3309 10 NYCRR 80.138 in New York authorized clinical directors (physicians, physician assistants or nurse practitioners) to prescribe naloxone to train overdose responders and anyone likely to experience or witness an overdose. In New York City, naloxone can also be directly dispensed by pharmacies without a prescription and with or without patient insurance.

Overdose Education and Naloxone Distribution (OEND) programs have therefore emerged in multiple settings as a resource for people at risk of witnessing or experiencing an overdose. In this paper, we describe how our primary-care-based program, The Respectful and Equitable Access to Comprehensive Healthcare (REACH) Program at Mount Sinai Hospital in New York City, developed an OEND model to address the opioid epidemic in our community inclusive of the general public [not only targeting people who use drugs (PWUD)], and the broader impact of this initiative in the larger hospital system. Our harm reduction-oriented approach involved interventions targeting stakeholders at many levels in our healthcare environment and fostering new partnerships. OEND sessions provided an opportunity to destigmatize PWUD and equip the public with a tool to potentially prevent an opioid overdose death. In this paper, we describe our OEND programs and address the multitude of challenges and lessons learned from our experiences as an OEND program. The authors of this paper implemented the community case study described.

## Background and Rationale

Literature on the impact of take-home naloxone on PWUD first emerged in the late 1990s. OEND was created as a harm reduction tool by PWUD and advocacy agencies to empower PWUD and their communities. (Dettmer et al., 2001; Maxwell et al., 2006; Winhusen et al., 2017). Syringe Service Programs (SSP) are ideal places for OEND because they provide culturally relevant services designed to reach persons at high risk for experiencing or observing an opioid overdose (Lambdin et al., 2020). However, naloxone should be available in multiple settings to ensure equitable access to OEND to all individuals, including those that do not access harm reduction services through SSPs. Recent articles have focused on pharmacies and health systems as viable sources for broader naloxone distribution to bystanders and substance users in rural and urban areas (Drainoni et al., 2016; Akers, Hansen, and Oftebro, 2017; Devries, Rafie, and Polston, 2017; Morton et al., 2017). A pilot study developed a four-step program for OEND at The Veterans Health Administration (VHA) that focused on identifying target populations, garnering support, training staff members, and implementation (Peckham and Boggs, 2016). Inpatient medical OEND integration was explored in another pilot study that enrolled newly admitted patients who had used opioids in the year before admission, exposed them to a short training video, and gave them a take-home naloxone supply (Jakubowski et al., 2019).

Several barriers to OEND in clinical settings were identified in these and other studies For example, Peckham et al. found that when implementing the OEND pilot program at VHA, providers felt that only mental health or substance use providers should distribute take-home naloxone due to familiarity with substance use disorders. Providers raised concerns of a possible increase in opioid consumption given the availability of the reversal agent. Informing providers of previous programs’ successes in reducing opioid use was key for implementation (Peckham et al., 2016). Another study attempted a broader evaluation of opioid overdose prevention initiatives, by surveying 18 naloxone training programs in Ohio and identifying barriers to widespread medical distribution. The authors found that stigma surrounding opioid use and the cost to purchase and dispense naloxone were preventing optimal implementation (Winstanley et al., 2016). The pilot study examining OEND among hospital inpatients also described limited hospitalization times as a barrier to effectively training all inpatients. Furthermore, they proposed expanding the classification of opioid-related events as a universal assessment rather than targeting only those admitted with opioid-related diagnoses to broaden the eligible pool of participants (Jakubowski et al., 2019).

The REACH Program at Mount Sinai Hospital is located in East Harlem, an area of New York City with a high prevalence of opioid overdose deaths. REACH staff implemented OEND during community outreach events and in some settings OEND was accompanying by hepatitis C virus (HCV) testing. REACH also pioneered multiple initiatives that impacted other hospital areas, including creating a curriculum for medical students and residents, providing a once-a-month naloxone training outside of the main hospital cafeteria, and working together with the emergency services department to identify patients with a substance use disorder for overdose education and naloxone distribution. Our findings are based on almost four years of experience (2017–2020). We outline REACH’s naloxone program structure while detailing the successes and difficulties of OEND in three settings: 1) community outreach, 2) primary care clinic, and 3) hospital setting.

## Methods

### Setting

The REACH program is a community-based program at the Mount Sinai Hospital, a large urban academic medical center. The REACH Program receives funding from the New York City Department of Health and Mental Hygiene. n.d. “Naloxone”, 2020 (NYC DOHMH) and New York State Department of Health (NYS DOH). In April 2017, the REACH Program became a legally registered Opioid Overdose Prevention Program (OOPP) funded by the NYC DOHMH to provide overdose education and naloxone training.

The REACH Program has two main components: 1) an outpatient primary care clinic for persons with HCV infection and/or substance use disorders, and 2) community outreach. The program is located in an epicenter of unintentional drug overdoses in NYC. East Harlem experienced 56.1 deaths per 100,000 residents in 2018 compared to 20.5 per 100,000 residents in the same year in NYC (NYC DOHMH, 2019). The outreach team at REACH began by providing OEND in East Harlem and expanded training programs over time to other boroughs (Bronx, Queens, and Brooklyn) in need of overdose prevention training. Efforts to recruit interested participants started during outreach events at health-fairs, substance use programs, homeless shelters, faith-based organizations, and re-entry programs. Community-based point of care HCV testing was a major ongoing focus of the program, and OEND was offered alongside this service. Naloxone kits were provided to participants for free once training was completed.

The REACH OEND efforts in the outpatient setting led to a domino effect providing the opportunity to impact multiple areas in our health system. This effort began with our model of “training the trainers” which allowed the program to expand naloxone distribution by increasing the number of individuals able to train others. The training follows two different formats depending on the target audience: 1) For clinical staff, we utilized the “train-the-trainer” model, with the purpose of educating attendees on how to train other staff and patients on OEND, 2) For bystanders during outreach sessions, we provided shorter and more personalized training (one-on-one).

### Program Description-Opioid Overdose Prevention Training

Our program provided overdose education and distribution of intranasal naloxone (Narcan®) rescue kits to self-identified or interested individuals likely to experience or witness an overdose. The training was performed by five certified patient navigators and one peer outreach worker from REACH based on the NYC DOHMH “Save a life. Carry naloxone” training. The training objectives were 1) to increase the number of people able to train others to distribute naloxone (“Train-the-trainer” sessions), 2) train the community to become opioid overdose responders, 3) present naloxone as a harm reduction tool 4) promote REACH services including primary care for PWUD, HCV testing and treatment, and office-based buprenorphine treatment and, 5) education on how to refer a patient by phone or email to REACH.

Naloxone rescue kits included a patient handout with instructional information in English/Spanish, two non-latex gloves, one rescue breathing mask, a certificate of completed training, information to call REACH for naloxone refills, and two naloxone (Narcan®) single-use intranasal spray (0.4 ml in each nostril for a total of 0.8 ml). Participants were instructed to administer one dose and wait 1–2 min. A second dose was included if the first dose failed to reverse overdose symptoms. At the end of each training, participants were asked to complete information regarding demographics (gender, race/ethnicity, zip code of the residence), how they planned to use the naloxone rescue kit and, if they had received a kit before.

Naloxone rescue kits were given to participants without an individual prescription under a standing order, allowing for distribution without a physician present and without any cost.

The curriculum for the trainings included education and techniques in overdose prevention and management including: 1) definition of an opioid, 2) fentanyl’s presence in other drugs, 3) reducing overdose risk, 4) naloxone as a harm reduction tool, 5) assessing for an opioid overdose, 6) seeking help by calling 911, 7) delivering intranasal naloxone, 8) information on aftercare including potential withdrawal symptoms (recue position), 9) information about the Good Samaritan Law, and 10) brief education about REACH Program services.

### Types of Trainings

#### Train-the-Trainer Model

Trainings directed to clinical staff including medical students, medical residents, nursing staff, and other health care staff. REACH utilized an hour long PowerPoint presentation (see Supplementary Presentation S1) based on “Save a life. Carry naloxone” (Naloxone–NYC Well (cityofnewyork.us). The trainings addressed preventing, recognizing, and reversing opioid overdose, an overview of the most current data about unintentional drug deaths in New York State, and basic information about REACH services. Sessions emphasized naloxone as a harm reduction tool, and participants learned how naloxone could be a point of engagement for PWUD to discuss their drug use and potential treatment modalities. After the session, attendees were encouraged to train others utilizing the “Train-the-trainer” model. The intent of this approach was to expand OEND throughout the hospital. Sessions were led by a physician or patient navigator with a background in health sciences.

Training requests were at times for one-off sessions and at other times for a more integrated multicomponent collaboration between the REACH program and various hospital divisions.

##### A1) Medical Students and Residents

Medical students at the Icahn School of Medicine at Mount Sinai have a harm reduction interest group. This group approached REACH as a coalition partner, and together medical students and REACH staff created a harm reduction educational power point presentation accessible to all first-year medical students. This presentation was subsequently included as part of medial students’ orientation, and served as a recruitment tool for new coalition participants. Additionally, the OOPP’s Medical Director created a 30 min presentation for internal medicine resident physicians. The presentations followed the “train-the-trainer model” with the intention of continued training of incoming first-year medical students by residents.

##### A2) Nursing Staff at Outpatient Clinics

REACH also provided training for outpatient nursing staff in a variety of primary care and specialty settings (e.g., pain management). Providers that identified a patient at risk of overdose or a family member of someone at risk were instructed to refer patients to trained nurses in these settings who then provided OEND to participants. The REACH program stocked nursing stations in these outpatient clinics with take-home-naloxone for distribution.

##### A3) Other Healthcare Staff

REACH provided OEND to healthcare professionals from various specialties familiar and unfamiliar with substance use disorders. The training encouraged the providers to call the REACH program at the time of conducting OEND to determine the appropriateness of a referral and, if with an at risk-patient, to arrange a “warm handoff’ to a REACH staff member (patient navigator or peer outreach worker). If the patient was interested, an appointment to the program was offered and scheduled at that time.

### Training the Community

#### One-on-One Trainings

The bulk of these efforts by the REACH outreach team (patient navigators and peer outreach worker), was in providing OEND trainings in settings outside the hospital, such as public events, substance use treatment facilities, or re-entry programs. The goal was to reach the community in diverse settings to capture at-risk populations and educate a wide range of community members. This type of training covered the nine points mentioned in the curriculum in a more summarized manner, highlighting key elements; assessing for an opioid overdose, seeking help by calling 911, delivering intranasal naloxone, information on aftercare including potential withdrawal symptoms (recue position), and information about the Good Samaritan Law. The trainings were designed to assess and build on potential overdose bystander knowledge in a 10–15 min one-on-one talk with any interested attendees.

Because the OEND was met with immediate interest in the community we increased the number of outreach sessions and broadened our presence. As one example, we created partnerships with churches in high-risk neighborhoods. Participation during outreach sessions offered an opportunity to further educate the community about REACH services. By word-of-mouth, participants referred peers for training.

Monthly quality improvement meetings were held with the medical director, program director, project coordinator, and relevant staff to discuss progress on the project, new initiatives, improve quality, and review data. The data collection informed the meetings, shaped the program's next steps, and provided a monthly space to reflect on lessons learned. REACH team also attended a yearly meeting with the funder and submitted monthly and quarterly reports that provided additional opportunity to reflect on and synthesize lessons learned in the project.

### Opioid Overdose Prevention Programmatic Adaptation During COVID-19

During the COVID-19 pandemic, many OOPPs had to modify their services of overdose education and naloxone distribution. In an effort to fill this gap and support increased access to free naloxone during the pandemic, the NYC Department of Health and Mental Hygiene (NYC DOHMH) launched the NYC Emergency Overdose Rescue Kit Pharmacy Pilot in June 2020. The pilot was established by a collaboration between the NYC DOHMH and two chain pharmacies in the 15 neighborhoods with the highest rates and numbers of overdose mortality. Pharmacies participating in this pilot (*n* = 15) dispensed free overdose rescue kits to any individual who requested one. Kits are accessible without an ID or insurance coverage. NYC DOHMH posted a list of participating pharmacies on their website.

Given incidental patient reports about difficulty obtaining naloxone from pharmacies, REACH decided to evaluate the NYC Emergency Overdose Rescue Kit Pharmacy Program given its importance as a distribution channel during the COVID-19 pandemic. REACH obtained an IRB exemption to implement a protocol to visit these 15 pharmacies and determine ease of accessibility and barriers to obtaining naloxone. REACH staff visited these pharmacies unannounced and without identifying themselves requesting pharmacists/technicians to dispense naloxone. Information was collected regarding availability, cost, and formulation (intramuscular, nasal spray, etc.).

### Data Collection

The REACH OEND Project was reviewed by the Department of Medicine Quality Improvement Committee and it was designated as a Department of Medicine Quality Improvement Project and not human subject’s research. The data presented on the REACH OEND program are quality improvement metrics which were routinely collected as part of REACH’s clinical operations for the purpose of program evaluation and improvement. We also present findings from the Pharmacy Outreach project related to the larger REACH OEND program.

As a requirement to receive a naloxone kit, each participant filled out a Naloxone Recipient Form (NRF) which included basic demographic questions such as zip code, race and/or ethnicity, age, experience receiving or using naloxone, and the reason to acquire a kit. The form also had a section filled by the session trainer which specified the program and address of the OOPP conducting the training, the number of kits received by the trainee, expiration date of the naloxone, location of the training, and the name of the trainer. The back of the NRF provided a designated space for the participant to write their name, although participants were advised that a signature or initials would be suffice and were given the option to refuse or leave the section blank if desired.

Information collected from the NRF was then used to create a database in Excel format which served as the reference for the information sent to the NYC DOHMH in the Site Summary Sheet each month and the Quarterly Reports submitted through NYCoverdose.org. In addition to the information in the NRF, the database collected the number of trainings per reporting period, type of organization or location where the training was performed, and the overdose reporting forms filled or communicated to the OOPP.

## Results

### Community-Based Overdose Education and Naloxone Distribution

From April 2017 to December 2020, the REACH Program distributed 4,235 naloxone kits to 3,906 participants at 290 direct trainings and trainings provided by those we trained. The majority of trained participants identified primarily as black (29.4%), Hispanic (24.2%), or white (22%), and the mean age was 42 years (range 14–97 years old). Data collection did not capture demographics related to gender.

**FIGURE 1:**
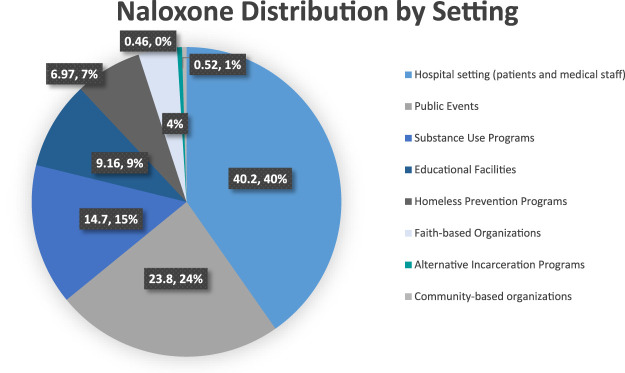
Naloxone distribution by setting.

Information collected from the NRF indicated that the three top reasons for getting a kit were “Just in case I see someone overdose” (59.3%), “I’m worried that someone I know will overdose OR that I will overdose” (20.2%) and “I work with people who use drugs as part of my job” (18%). There were 275 overdose prevention trainings conducted by REACH in which 3,308 people were trained. Of the 3,308 trained participants, almost all (3,301) agreed to receive one or more kits for a total of 3,724 kits distributed. See Figure 1 for the setting in which these 3,724 kits were distributed.

### Naloxone Distribution by Setting

#### Hospital Setting

**FIGURE 2:**
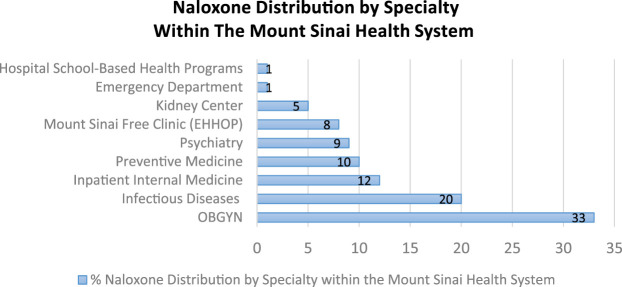
Naloxone distribution by specialty within the Mount Sinai health system.

We distributed 40% (see Figure 1) of the take-home-naloxone kits within Mount Sinai Hospital. Distribution settings included the hospital’s cafeteria, a diverse range of specialty settings within the Mount Sinai Hospital (see Figure 2), the waiting rooms of outpatient clinics, the medical student run free clinic for East Harlem residents, the emergency department, nurse stations at outpatient clinics, student harm reduction coalition trainings to first-year medical students, trainings to first-year Mount Sinai residents, REACH support groups and Community Advisory Board meetings. In these settings, we targeted the general public, health care staff, and patients utilizing hospital services. When training medical students, medical residents, and staff from various specialties we utilized a power point presentation. However, we used 10–15 min one on one talks when training bystanders at the hospital cafeteria, waiting rooms, free clinic, emergency rooms, and nurse stations as well as during REACH hosted events.

##### a) Naloxone Education Outside the Hospital Cafeteria

The hospital cafeteria is located in the main hospital, open to Mount Sinai employees, patients and family members. Our program coordinated a monthly session open to the public (patients clinic and non-clinic staff) during high traffic times, outside the entrance to the hospital cafeteria. The sessions started in August 2019 and a total of 213 kits were distributed among participants over the course of 8 months until the COVID-19 pandemic began. We found that these sessions enabled our team to interact with a wider variety of staff than did our targeted trainings. Additionally, we expanded education about REACH Program services. These trainings increased the number of referrals to REACH (for services including primary care for PWUD and to provide additional OEND trainings).

##### b) Naloxone by Specialty

REACH was able to train personnel in nine specialty disciplines to distribute 91 naloxone kits (Figure 2).

##### c) Involving Medical Students

Medical students at the Icahn School of Medicine at Mount Sinai quickly became interested in helping to distribute naloxone and educate community members about overdose prevention. REACH staff and medical students created a brief curriculum to increase medical student knowledge on substance use and harm reduction principles including doing rotations at syringe exchange programs. All first-year medical students were trained and received naloxone kits during orientation week. Additionally, trained medical students distributed naloxone to at-risk patients during emergency department overdose visits twice a month in the outpatient clinic waiting rooms, and during East Harlem Health Outreach Partnership (EHHOP) sessions. EHHOP is a free clinic at Mount Sinai hospital run by medical students and residents to serve the uninsured. Medical students also volunteered during REACH community outreach sessions.

##### d) Nurses Provide OEND to Patients

The REACH Program partnered with nurses at the Internal Medicine Associates (IMA) outpatient clinic. REACH is co-located within IMA which offers various primary care and specialty services. REACH provided naloxone training to the nurses via a 45 min Power Point presentation utilizing the train-the-trainer model. Our goal was to identify nurse champions who would in turn identify potential patients at risk, provide OEND and keep an inventory of the kits at each nurses station. However, after the training, only two nurses were interested in becoming champions. Most felt they could not incorporate the role into their daily tasks. We shifted our focus and asked providers (including residents that received the naloxone training by our team) to refer patients at risk or their family members to the nurses. Nurses provided 15 min one-on-one training with patients identified by the clinicians and collected the NRF forms. Our team kept an inventory of the five kits per firm (4 firms participated) and restocked them every two months.

#### Public Events

This setting represented 24% of our OEND efforts (see Figure 1). They included events hosted by the East Harlem community, such as the Juneteenth Health Fair, the Malcolm Shabazz Harlem Market, and a theater performance about substance use. Our team also attended events tailored to at-risk populations, such as one hosted by a re-entry program offering free haircuts. There were events hosted by REACH, such as a Medication Assistance Waiver Training for medical providers and the International Overdose Awareness Day (IOAD) remembrance event. We also participated at health fairs for the Metropolitan Transportation Authority staff.

Additionally, building on our hepatitis C testing collaboration with the Georgian community, we participated in an annual festival celebrated by Georgians. We offered OEND at a supermarket in one of the top five zip codes for rates of unintentional drug poisoning (overdose) (NYC DOH MH 2012-2013). Other public events were hosted by the Mount Sinai hospital or community members. We used a 10–15 min one-on-one talk model at all events. In some settings we offered hepatitis C testing, but in the majority, we only did OEND. At some of the events, we encountered an audience with previous naloxone experience. For example, at the IOAD, participants shared their personal and family experiences with overdose and naloxone administration.

#### Substance Use Programs

We collaborated with inpatient substance use treatment programs, transitional housing programs including halfway housing, long-term residential, and outpatient substance use treatment programs, and a nursing residential healthcare facility. Based on the program’s needs, we provided only OEND, or we offered naloxone along with HCV testing. While offering both services, we had two approaches: 1) during HCV testing, participants completed a brief intake that included risk factors. If a patient had active substance use or was at risk of an opioid overdose, our outreach team offered naloxone via a one-on-one training as the participant waited for the rapid antibody HCV testing results. 2) If we had a larger group, we provided a 15 min oral presentation on naloxone and then offered HCV testing. As in the public events setting, REACH encountered individuals with previous knowledge of naloxone administration, opioid overdose, and in some cases, death due to an overdose.

#### Educational Facilities

The REACH Program partnered with community health centers at various community colleges in NYC. Our team provided OEND during the community health centers’ presentations of services to first-year students. At other colleges, our team distributed naloxone through 15 min one-on-one talks at college health fairs. We trained research coordinators and staff at a university conducting a study on PWUD. Additionally, we trained health staff from an adolescent health center for students at a charter school.

#### Community-Based Organizations

We collaborated with the Mexican consulate by giving an oral 45 min Power Point presentation in Spanish to participants in the waiting room of the consulate. We partnered with a community center in East Harlem by offering OEND to groups run by agencies co-located in the community center. Furthermore, we hosted several sessions at an LGBTQ center in NYC. In some of these sessions, REACH offered HCV testing and OEND to all participants utilizing the same approach described for the substance use setting.

#### Homeless Prevention Programs

The REACH Program teamed with agencies that provide temporary and permanent housing with supportive services to homeless individuals at various locations. In some of these sessions, REACH offered HCV testing and OEND to all participants utilizing the same approach described for the substance-use setting. Additionally, we distributed naloxone in the streets by collaborating with a mobile soup kitchen bus agency that traveled to at-risk neighborhoods targeting the homeless population. One of the locations was near one of the largest methadone programs in the country. OEND was provided in 10–15 min one-on-one sessions or in small groups. A bilingual (English/Spanish) REACH member provided culturally and linguistically appropriate training in Latinx neighborhoods. In this setting, we often encounter individuals’ already knowledgeable about naloxone administration, opioid overdose, and overdose death. Additionally, individuals in these settings often mentioned two main themes: 1) they were previously trained but did not get a kit after the naloxone training, or 2) owned a kit and wanted an additional one due to the high risk of overdose for themselves or people around them.

#### Faith-Based Organizations

REACH collaborated with one of the chaplains at the Center for Spirituality and Health at Mount Sinai's Icahn School of Medicine that introduced REACH to various faith-based organizations, including the Salvation Army. OEND was offered at small health fairs for members of the faith organizations or after religious services. OEND was provided by 10–15 min one-on-one sessions.

#### Alternative to Incarceration Programs

We hosted various group sessions at a re-entry from incarceration for program participants. We also provided a 45 min Power Point presentation to staff at a Brooklyn Courthouse.

### Naloxone Distribution During the COVID-19 Pandemic

#### Pharmacy Outreach Evaluation

During August and September 2020, REACH staff made a single visit to all 15 pharmacies that were part of the NYC DOHMH Emergency Overdose Rescue Kit Pharmacy Pilot. These pharmacies were located in all five boroughs Bronx (5), Brooklyn (3), Manhattan (4), Staten Island (2), and Queens (1). We evaluated 1) the percentage of pharmacies that had naloxone in stock; 2) the types of naloxone available; 3) the reason for not having naloxone in stock; and 4) whether education was provided when the kit was dispensed.

Of the 15 pharmacies, 10 (66.6%) pharmacies dispensed naloxone to our staff. When dispensed, pharmacy staff did not request ID or insurance information. All kits were given without any cost and within a few minutes of speaking with pharmacy staff. All the kits contained two doses of naloxone nasal spray (Narcan®), two non-latex gloves, a face shield for rescue breathing, and an insert with Spanish/English instructions for responding to an overdose.

At the other five (33.3%) pharmacies, our staff was not able to obtain naloxone. Three pharmacies did not have naloxone in stock when visited. One pharmacy refused to dispense naloxone without a prescription, and one pharmacy was closed on weekends and had limited hours. These results were shared with the NYC DOHMH. In response, NYC DOHMH actively worked with pharmacies to ensure program awareness and adequate naloxone stock. Additionally, NYC DOHM updated the online participating pharmacies list and included the business hours and phone numbers of each pharmacy participating in the pilot.

### REACH OEND Program Shift During COVID-19

Our program is located at what was the epicenter of the U.S. COVID-19 pandemic during March-April of 2020. We had to find creative ways to continue to provide naloxone trainings during this time. NYC DOHMH authorized OOPPs to mail naloxone kits to participants in response to the public health emergency. Medical students attempted to contact a total of 509 patients that were identified as at risk for an opioid overdose (patients prescribed buprenorphine or with history of active opioid use). Of those 509, 338 were patients from the REACH program; the remainder were patients in the Emergency Department or Inpatient services that presented with or were at risk of an overdose. The medical students provided training to 90 of these patients, 84 of whom received an overdose kit by mail or through the attending physician/care team at discharge. Six patients who received remote naloxone training did not want a kit mailed for reasons including already having one at home. In addition, we began a monthly Zoom OEND session open to the public. Sessions were promoted using social media (Instagram and Twitter) and through our program newsletter. The sessions were hosted by one of our team members for approximately 15 min utilizing the same principles of our in-person sessions, and naloxone kits were mailed to participants after the sessions. From May to December 2020, we hosted 14 virtual trainings and mailed 276 naloxone kits.

## Discussion

Expanding naloxone distribution for treatment of opioid overdose has been a focus of policy at local, state and national levels in the U.S. Our program illustrates several methods by which naloxone distribution can be facilitated.

### Lessons Learned

#### Lessons Learned From Training Bystanders

##### Hepatitis C Virus Testing and Naloxone Distribution

Before becoming part of the OOPP, our community outreach was focused solely on HCV testing and education; adding naloxone distribution/overdose education to our services provided an opportunity to partner with a broader range of organizations. This addition increased our collaboration with other programs such as homeless shelters, and court-mandated residential and chemical dependency treatment programs and, in turn, allowed us to engage a patient population that is often distrustful of the healthcare system and build more meaningful relationships with the community.

While hosting training events, many participants disclosed the need for additional services such as stigma-free risk reduction counseling, office-based buprenorphine treatment, HCV testing or treatment, overdose response training, mental health services that offer support groups. Naloxone distribution provided an entry point into care, and our team was able to either provide information about services to participants and family members or schedule an appointment for requested services on the spot.

##### Reaching Participants With Limited Literacy and/or English Proficiency

Naloxone education poses various challenges depending on the setting and population. Many program participants had limited literacy and/or English proficiency. To overcome these challenges, we aimed to have a bilingual staff member in locations where we knew we would encounter a large Spanish speaking population and both the training literature and data collection forms were printed in Spanish. In the case of the Georgian community, we solicited volunteers who could provide cultural context and translation into Georgian and Russian (including translation of the NRF forms).

#### Lessons Learned From Training Clinical Staff

##### REACH Interfacing With the Larger Health Care System

REACH’s monthly hospital cafeteria trainings made clear to us the tremendous overlap across health care professionals, other hospital staff, community members, and patients. Often those trained at these events were members of two or more of these groups. Many of these trainings were done with a diverse group of attendees, including the general public, health staff, PWUD, and family members of people at-risk for an opioid overdose. We found that holding public OEND with a heterogeneous group membership had the potential to decrease institutional stigma associated with drug overdose. Based on comments received from trainees and new referrals from new departments to provide trainings; we believe that these in-house trainings contributed to shifting the culture within Mount Sinai Hospital around the care for opioid use disorder and promoted a more humane treatment of survivors of opioid overdose.

The REACH Program OOPP initiated actions within the larger health system to address opioid overdose risk. Naloxone kits were made available at nurses’ stations in the primary care outpatient clinics and providers and nurses were able to give naloxone kits to at-risk patients. More could and should be done. For example, if a patient is prescribed an opioid, the electronic health system could trigger a reflex order for naloxone to the patient’s preferred pharmacy. As another example, the Mount Sinai emergency department started a collaboration in early 2020 that connects participants who have had a near fatal overdose to peer “wellness advocates” who are deployed to the ED to offer overdose education, (Welch et al., 2019), naloxone, linkage to care (including to the REACH Program) and supportive follow-up up for 90 days.

##### Medical Students and Residents Role in OEND

As part of an academic medical center, we expanded naloxone distribution to first-year medical students and resident physicians who, after being trained, were able to train others. The residents in turn increased referrals to REACH and to nurses able to provide naloxone training to identified patients.

##### Empowering Others to Expand Their Naloxone Knowledge (Train the Trainer)

This approached helped broaden our reach by training clinic and non-clinic staff on how to teach OEND to others. By empowering other hospital staff (medical students, residents and nurses) we increased sensitivity to conducting substance use assessments, and potentially decreased stigmatization of PWUD.

##### Collaboration With Other Medical Specialties

Sometimes OEND training was provided as a one-off event, and other times, as part of a multicomponent approach to address substance use in different hospital areas in. We noticed an increased interest in naloxone trainings in the second scenario. For example, when OEND was combined with training on buprenorphine treatment to specialty areas serving patients with a high prevalence of substance use disorders. Naloxone functioned as a tool for teams to collaborate on addressing patients’ substance use journeys.

### Lessons Learned as a Program

#### Naloxone Distribution by Setting

Overall, we realized that we succeeded in getting more participants interested in naloxone in some settings as compared to others. Based on audience engagement and trainers’ experience, we realized that settings like the hospital, substance use programs, and homeless prevention programs presented an audience very open to learning about naloxone. We noticed that at the substance use programs, some public events that target PWUD or staff working with PWUD, and at homeless prevention programs, most participants (including staff) knew about naloxone administration, overdose survival, and/or had experienced grief from losing a loved one to an overdose. As a natural progression during the presentation, participants and staff shared their own experiences. These moments allowed for clarification of misbeliefs and reinforced the need for adequate naloxone administration. If the training was done as a 10–15 one-on-one talk, REACH staff connected the participants with resources offered by REACH including appointments with one of our providers, and invitations to join the weekly harm reduction group or the Community Reinforcement Approach to Family Training (CRAFT) program.

On the other hand, at events where the public was diverse in naloxone knowledge, our team more often experienced less audience engagement, more stigmatized understandings of substance use, and a lacking of understanding of the purpose of naloxone. In the future, broadening future outreach to the public may help decrease stigma surrounding drug use and expand the use of naloxone.

### Lessons Learned During COVID-19

#### During the COVID-19 Pandemic

We recommend that OEND through Zoom training followed by mailing naloxone should continue post-COVID. PWUD face many barriers to care, and offering multiple options on how to engage in medical care should be best practice.

#### Pharmacies Naloxone Dispensing During COVID

More than 2,600 pharmacies throughout New York State have naloxone available without a prescription through a standing order (New York State Department of Health n. d.). Although dispensing naloxone through a standing order may seem like an advantage, there’s no widespread public knowledge of this option. Both “train-the-trainer” and bystander trainings provided by our program, aimed to address this knowledge gap.

While the NYC DOHMH provides funding and guidelines on how to obtain and replenish inventory of naloxone kits and collects and reports kit distribution metrics, its distribution and event protocol guidance is limited. Consequently, protocols on how to organize events and distribution are left to each OOPP. The New York State Department of Health does provide opioid-related data to support prevention efforts, including timely overdose reporting, which helps identify struggling communities. The NYC DOHMH web site provides information on upcoming training, community-based programs that can be contacted for free naloxone, and access to additional information about naloxone. The website also includes the ability to download the Stop OD NYC app, which provides guidelines to recognize and prevent opioid overdoses while indicating sources of naloxone close to the user (New York City Department of Health n. d.). Nonetheless, additional centralized guidance regarding naloxone distribution could be a useful tool for OEND programs.

### Program Challenges

#### Reporting Naloxone Used

At all training sessions, we encouraged participants to contact the REACH program or NYC DOHMH if they utilized one or both naloxone doses. Many REACH patients have recounted to their medical providers the experiences of using naloxone to save the lives of others or having their own lives saved by someone else using naloxone on them. Reporting the use of the kit to the OOPP from which it was obtained is not a priority at these moments. This presents a challenge to establish a metric of success through reversal reports, and has been a roadblock that other naloxone-based studies have frequently experienced (Enteen et al., 2010; Lewis et al., 2016; Lott and Rhodes, 2016; Bennett et al., 2011; Bennett et al., 2018; Siegler et al., 2017).

Although outcome metrics are limited for naloxone distribution, the ability to provide education on opioid overdose prevention to an at-risk population has merit in itself. While it is difficult to quantify the specific impact of naloxone distribution efforts, the NYC DOHMH announced in August 2019 that there was a decrease in the number and rate of overdose deaths from 2017 to 2018 after seven consecutive years of increasing drug overdose deaths (New York City Department of Health and Mental Hygiene, 2019). Emerging data shows that this positive trend has been dramatically reversed by the COVID-19 pandemic (New York State Department of Health and Mental Hygiene, 2021), making efforts to mitigate overdose deaths even more crucial than ever before.

#### Lack of Hospital Policies for Naloxone Distribution

Hospitals do not mandate staff OEND training or keeping naloxone stocked at inpatient units or outpatients clinics. Our program faced barriers when trying to train staff and establish protocols for naloxone education and distribution. For example, nurses did not always feel that distributing OEND was in their scope of work. We recommend that hospitals and health care facilities have clear guidelines for training all staff and educating at-risk patients and the general public. Without clear guidelines and policy recommendations, substance use and PWUD will remain stigmatized. The lack of a mandate also limits expansion of naloxone training to other agencies with clinical and non-clinical personnel. If we want to decrease overdose deaths, we need to implement naloxone education and access for all health care personnel and at risk patients and provide the funding to implement this.

#### Program Limitations

There are limitations to the work presented. The development of the OEND program occurred organically over time in coordination with the evolution of other REACH program initiatives; it was not guided by an a priori systematic framework or logic model. The work was conducted in an academic medical center setting with significant resources and may not be generalizable to other sites with fewer resources. Additionally, REACH benefited from strong institutional and administrative support for its initiatives to enhance services provided to people who use drugs and reduce associated stigma.

#### Program Recommendations

• Provide OEND alongside other services that are of interest to PWUD
• OEND trainings should be developed for populations with limited literacy and/or English proficiency
• Training non-clinical health care personnel by utilizing the ‘train the trainer’ method can expand the reach of OEND
• Naloxone distribution presents an entry point to expand program collaboration
• Pharmacies can play an important role in OEND
• Holding public OEND trainings with heterogeneous group membership has the potential to decrease the stigma associated with drug overdose.
• OEND guidelines should be established for all hospitals and health care facilities. All health staff should carry naloxone.

## Conclusion

The opioid epidemic in the United States requires urgent attention. While national policies in the last few years have begun to encourage naloxone distribution as a safe medication to combat opioid overdose fatalities, these measures must result in clear guidelines for health care institutions across the United States in order to be effective in reaching the most vulnerable communities. The REACH program at Mount Sinai Hospital represents a comprehensive model to train bystanders and medical providers to use naloxone while distributing the medication through connections with community partners and hospital staff. REACH’s community-based, harm-reduction approach to overdose prevention and primary care has allowed for outreach through programs previously not linked to Mount Sinai Hospital. While these connections have facilitated increased naloxone distribution in communities across NYC, they would be greatly enhanced if integrated into a systematic and coordinated health system response to the treatment for patients at risk of overdose. Although quantifiable data on overdose reversals with naloxone has been difficult to obtain because of low reporting rates, the successes and roadblocks encountered during REACH’s almost four years of experience as an OOPP can meaningfully shape future policy initiatives In NYC and across the nation. In the process, community-based outreach programs can continue to play an integral role in fighting the opioid epidemic and furthering the agenda to create a more coordinated multi-component response.

## Data Availability

The raw data supporting the conclusion of this article will be made available by the authors, without undue reservation.

## References

[B1] Bazazi A. R. Zaller N. D. Fu J. J. Rich. J. D. (2010). Preventing Opiate Overdose Deaths: Examining Objections to Take-Home Naloxone. J. Health Care Poor Underserved. 21 (4), 1108–1113. 10.1353/hpu.2010.0935 21099064 PMC3008773

[B2] Bennett A. S. Bell A. TomediHulsey L. Hulsey E. G. Kral A. H. (2011). Characteristics of an Overdose Prevention, Response, and Naloxone Distribution Program in Pittsburgh and Allegheny County, Pennsylvania. J. Urban Health. 88 (6), 1020–1030. 10.1007/s11524-011-9600-7 21773877 PMC3232410

[B3] Bennett A. S. Bell A. Doe-Simkins M. Elliott L. Pouget E. Davis C. (2018). Alice Bell, Maya Doe-Simkins, Luther Elliott, Enrique Pouget, and Corey Davis.from Peers to Lay Bystanders: Findings from a Decade of Naloxone Distribution in Pittsburgh, PA. J. Psychoactive Drugs. 50 (3), 240–246. 10.1080/02791072.2018.1430409 29424656

[B4] Council of Economic Advisers (2020). “Council of Economic Advisers Report: The Underestimated Cost of the Opioid Crisis.” the White House. Available at: https://www.whitehouse.gov/briefings-statements/cea-report-underestimated-cost-opioid-crisis/

[B5] Dettmer K. Saunders B. Strang J. (2001). Take home Naloxone and the Prevention of Deaths from Opiate Overdose: Two Pilot Schemes. BMJ. 322, 895–896. 10.1136/bmj.322.7291.895 11302902 PMC30585

[B6] Division D. C. D. (2018). 5-Point Strategy to Combat the Opioid Crisis. Digital Communications Text. U.S. Department of Health and Human Services. HHS.Go. Available at: https://plus.google.com/+HHShttps://www.hhs.gov/opioids/about-the-epidemic/hhs-response/index.html (Accessed May 8, 2018).

[B8] Doyon S. Aks S. E. Schaeffer S. (2014). Expanding Access to Naloxone in the United States. Clin. Toxicol. (Phila). 52 (10), 989–992. 10.3109/15563650.2014.968657 25283253

[B10] Haegerich T. M. Jones C. M. Cote P. O. Robinson A. Ross L. (2019). Evidence for State, Community and Systems-Level Prevention Strategies to Address the Opioid Crisis. Drug Alcohol Depend. 204 (November), 107563. 10.1016/j.drugalcdep.2019.10756311 31585357 PMC9286294

[B11] Hedegaard H. Warner M. Miniño A. M. (2017). Drug Overdose Deaths in the United States, 1999–2016 NCHS Data Brief, No 294. Hyattsville, MD: National Center for Health Statistics. 2017.

[B12] Jakubowski A. Pappas A. Isaacsohn L. Castillo F. Masyukova M. Silvera R. (2019). Development and Evaluation of a Pilot Overdose Education and Naloxone Distribution Program for Hospitalized General Medical Patients. Subst. Abus. 40 (1), 61–65. 10.1080/08897077.2018.1518836 30475162 PMC6778336

[B13] Maxwell S. Bigg D. Stanczykiewicz K. Carlberg-Racich S. (2006). Prescribing Naloxone to Actively Injecting Heroin Users: a Program to Reduce Heroin Overdose Deaths. J. Addict. Dis. 25, 89–96. 10.1300/J069v25n03_11 16956873

[B14] New York City Department of Health and Mental Hygiene (2019). Unintentional Drug Poisoning (Overdose) Deaths in New York City in 2018. Available at: https://www1.nyc.gov/assets/doh/downloads/pdf/epi/databrief116.pdf.

[B15] New York City Department of Health and Mental Hygiene (2021). Unintentional Drug Poisoning (Overdose) Deaths Quarters. New York City, 1-3,2020. April 2021. provisional-overdose-report-third-quarter-2020.pdf nyc.gov.

[B16] New York City Department of Health and Mental Hygiene. n.d. “Naloxone” (2020). Available at: https://www1.nyc.gov/site/doh/health/health-topics/naloxone.page (Accessed March 24)

[B17] New York State Department of Health and Mental Hygiene (2021). n.d. “Availability of Naloxone in Pharmacies. Available at: (accessed February 14, 2020).

[B18] Opioid Overdose Prevention Programs Providing Naloxone to Laypersons — United States, 2014 (2020). February 14, 2020. Available at: https://www.cdc.gov/mmwr/preview/mmwrhtml/mm6423a2.htm PMC4584734 26086633

[B19] Peckham A. M. Boggs D. L. (2016). The Overdose Education and Naloxone Distribution Program at a VA Hospital. Fed. Pract. 33 (11), 28–31. PMC6373704 30766147

[B20] Siegler A. Huxley-Reicher Z. Maldjian L. Jordan R. Oliver C. Jakubowski A. (2017). Naloxone Use Among Overdose Prevention Trainees in New York City: A Longitudinal Cohort Study. Drug Alcohol Depend. 179 (October), 124–130. 10.1016/j.drugalcdep.2017.06.029 28772172

[B22] Skolnick P. (2018). On the Front Lines of the Opioid Epidemic: Rescue by Naloxone. Eur. J. Pharmacol. 835, 147–153. 10.1016/j.ejphar.2018.08.004 30092179

[B23] Sohn M. Talbert J. C. Huang Z. Lofwall M. R. Freeman P. R. (2019). Association of Naloxone Coprescription Laws with Naloxone Prescription Dispensing in the United States. JAMA Netw. Open. 2 (6), e196215. 10.1001/jamanetworkopen.2019.6215 31225895 PMC6593960

[B25] Welch A. E. Jeffers A. Allen B. Paone D. Kunins H. V. (2019). Relay: A Peer-Delivered Emergency Department-Based Response to Nonfatal Opioid Overdose Am. J. Public Health. 109, 1392–1395 10.2105/AJPH.2019.305202 31415200 PMC6727316

[B26] Wheeler E. Jones T. S. Gilbert M. K. Davidson P. J. Centers for Disease Control and Prevention (Cdc) (2015). Opioid Overdose Prevention Programs Providing Naloxone to Laypersons - United States, 2014. MMWR Morb Mortal Wkly Rep. 64 (23), 631–635. 10.15585/mmwr.mm6436a6 26086633 PMC4584734

[B27] Winhusen T. Walley A. Fanucchi L. C. Hunt T. Lyons M. Lofwall M. (2017). The Opioid-Overdose Reduction Continuum of Care Approach (ORCCA): Evidence-Based Practices in the HEALing Communities Study. Drug Alcohol Depend. 217, 108325. 10.1016/j.drugalcdep.2020.108325 PMC7533113 33091842

[B28] Winstanley E. L. Clark A. Feinberg J. Wilder C. M. (2016). Barriers to Implementation of Opioid Overdose Prevention Programs in Ohio. Subst. Abus. 37 (1), 42–46. 10.1080/08897077.2015.1132294 26682929 PMC4848747

