# Supplementary material for: Community Case Study of Naloxone Distribution by Hospital-Based Harm Reduction Program for People Who Use Drugs in New York City
Source: Front Sociol. 2021 Jul 7;6:619683. doi: 10.3389/fsoc.2021.619683 (PMC8292929; doi:10.3389/fsoc.2021.619683)
Supplement: Supplementary file 1 [file Presentation1.PPTX]

## Slide 1
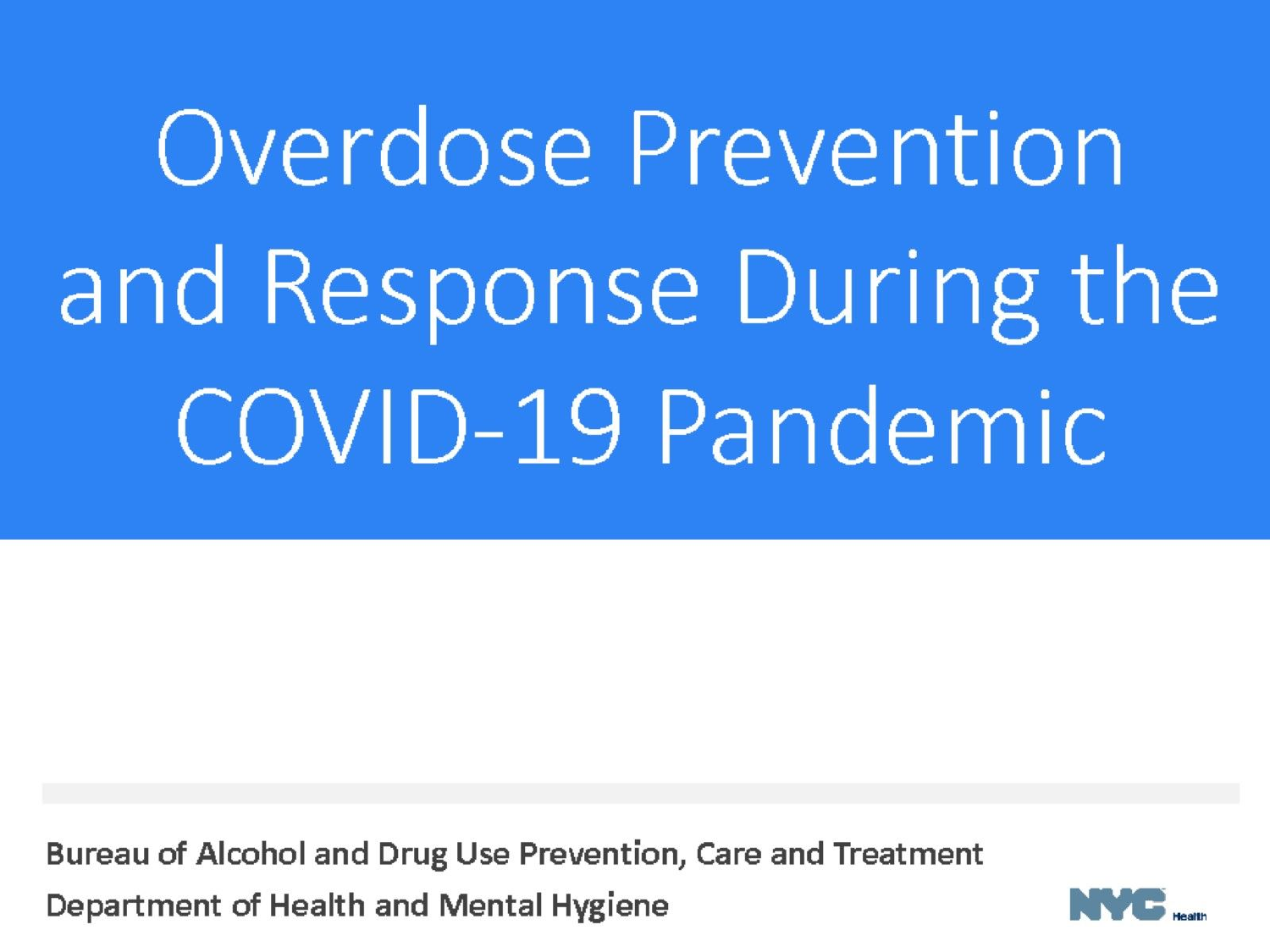

# Overdose Prevention and Response During the COVID-19 Pandemic

## Slide 2
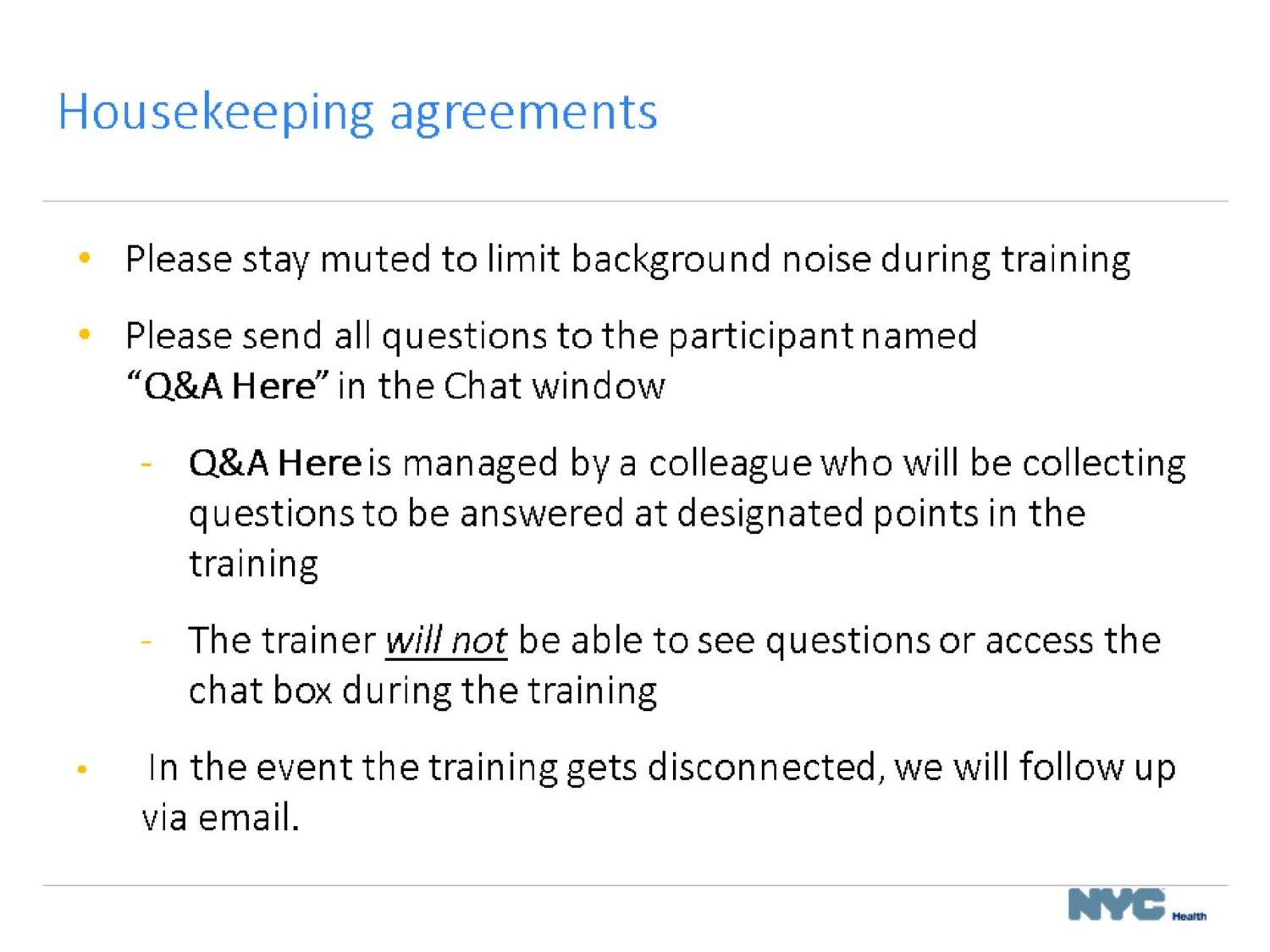

# Housekeeping agreements

## Slide 3
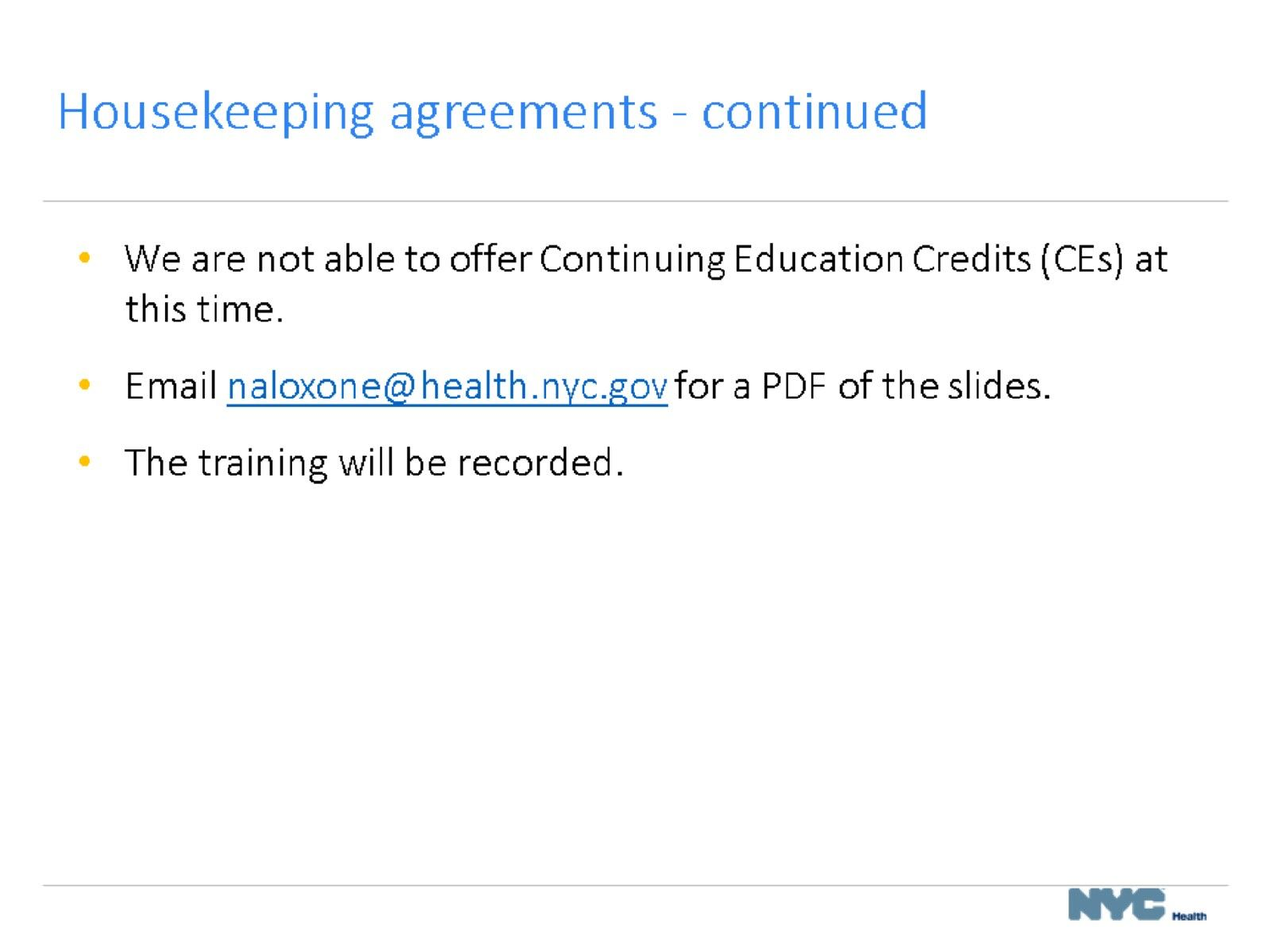

# Housekeeping agreements - continued

## Slide 4
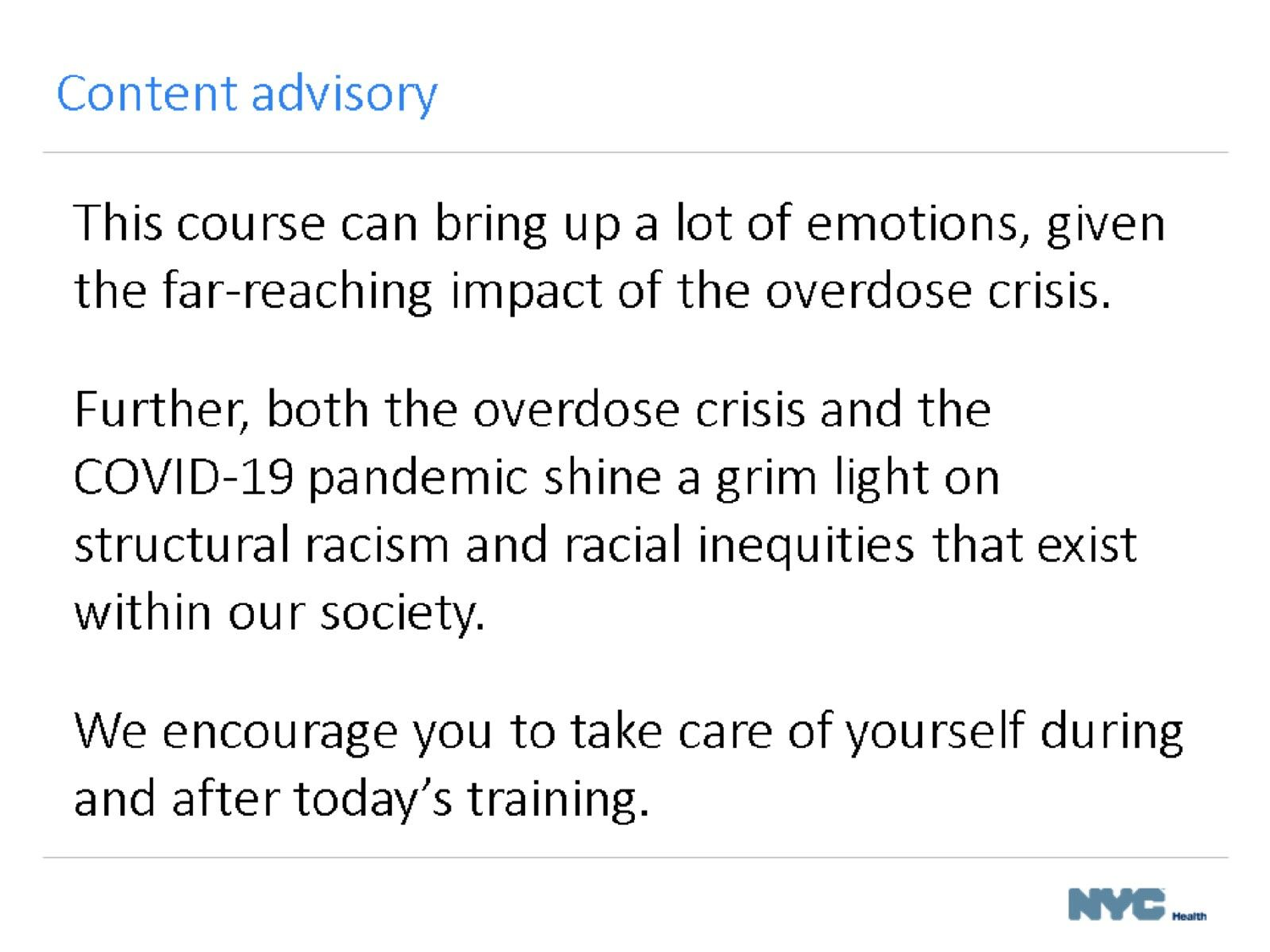

#

## Slide 5
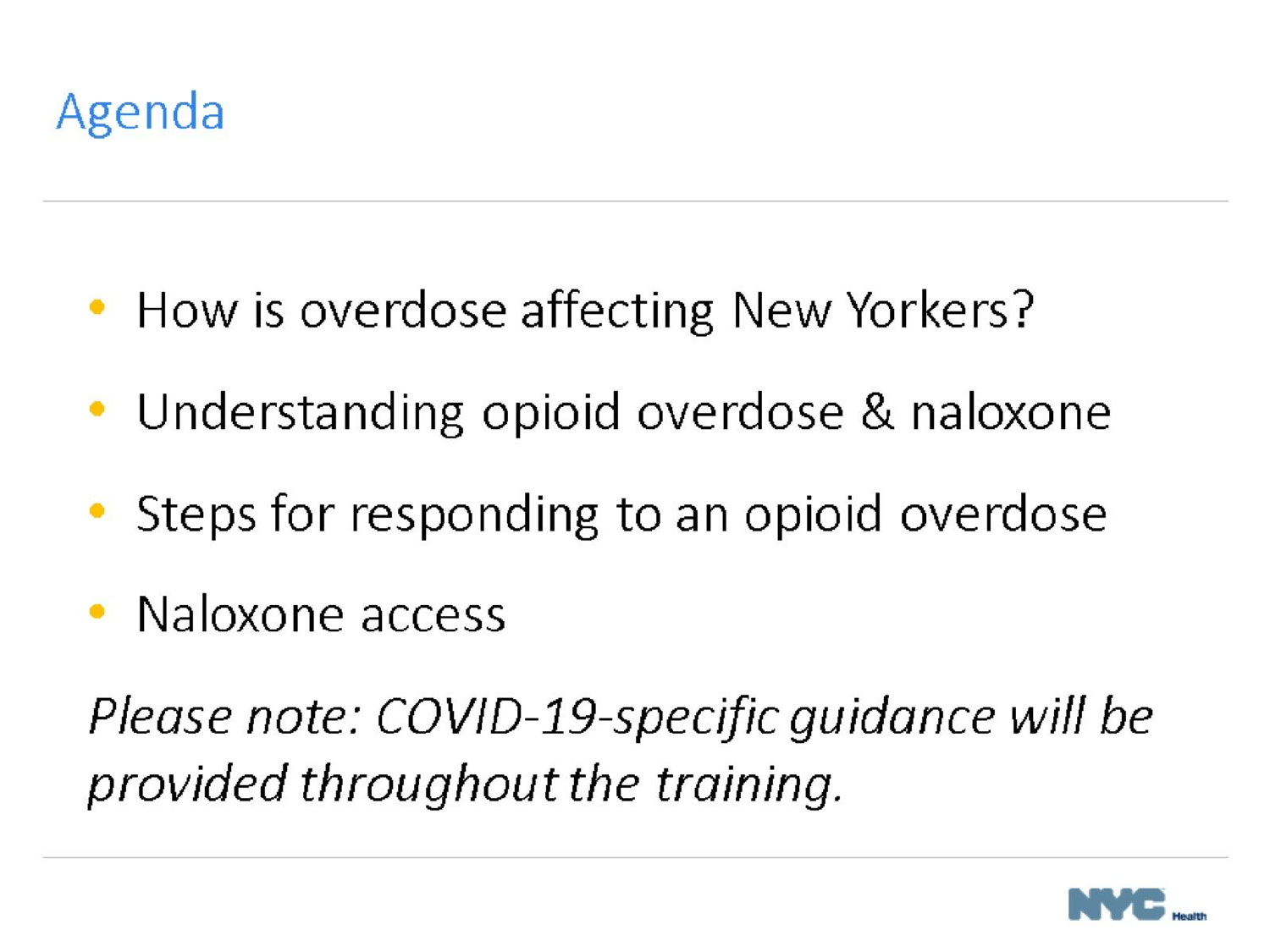

# Agenda

## Slide 6
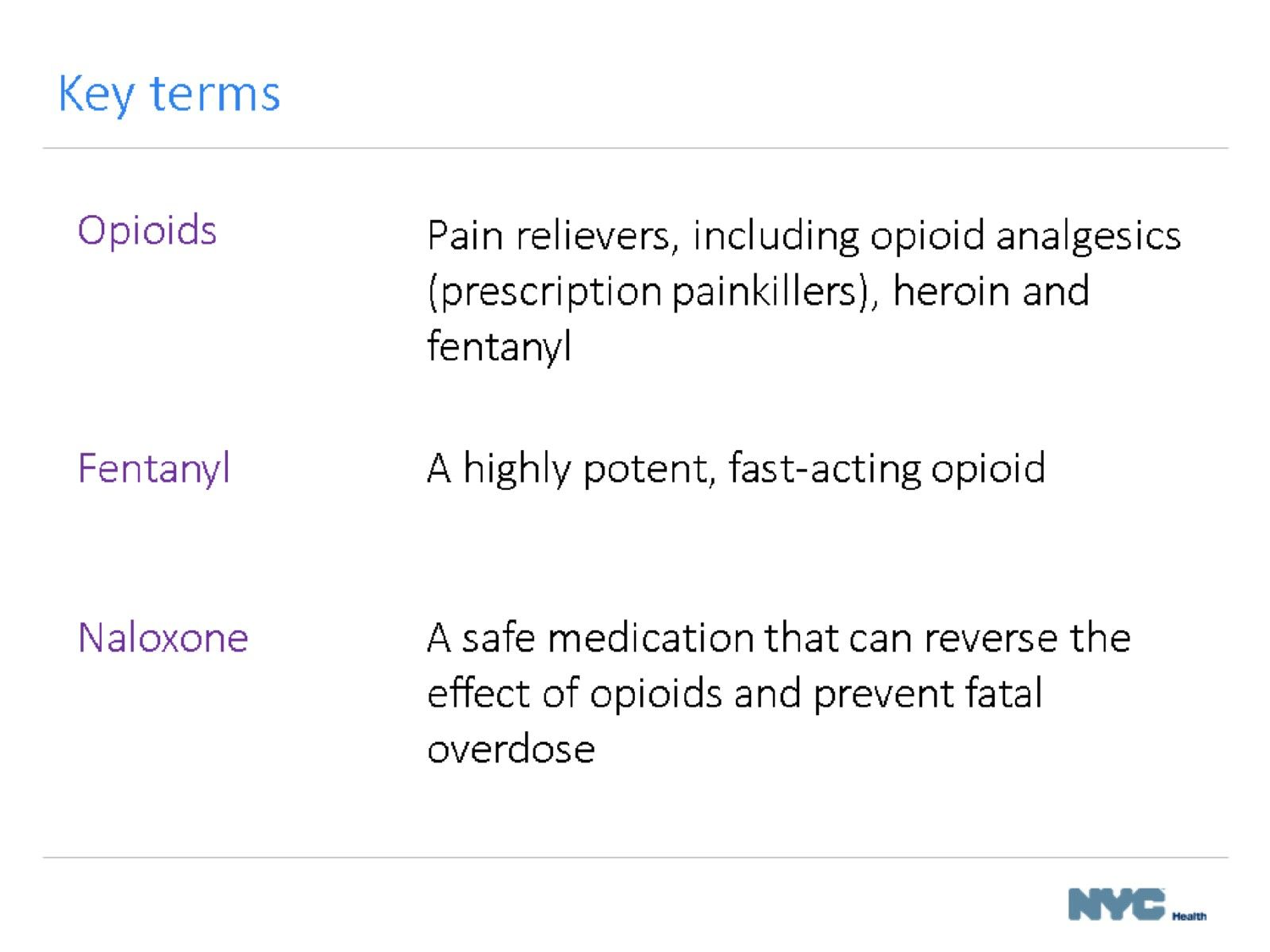

#

## Slide 7
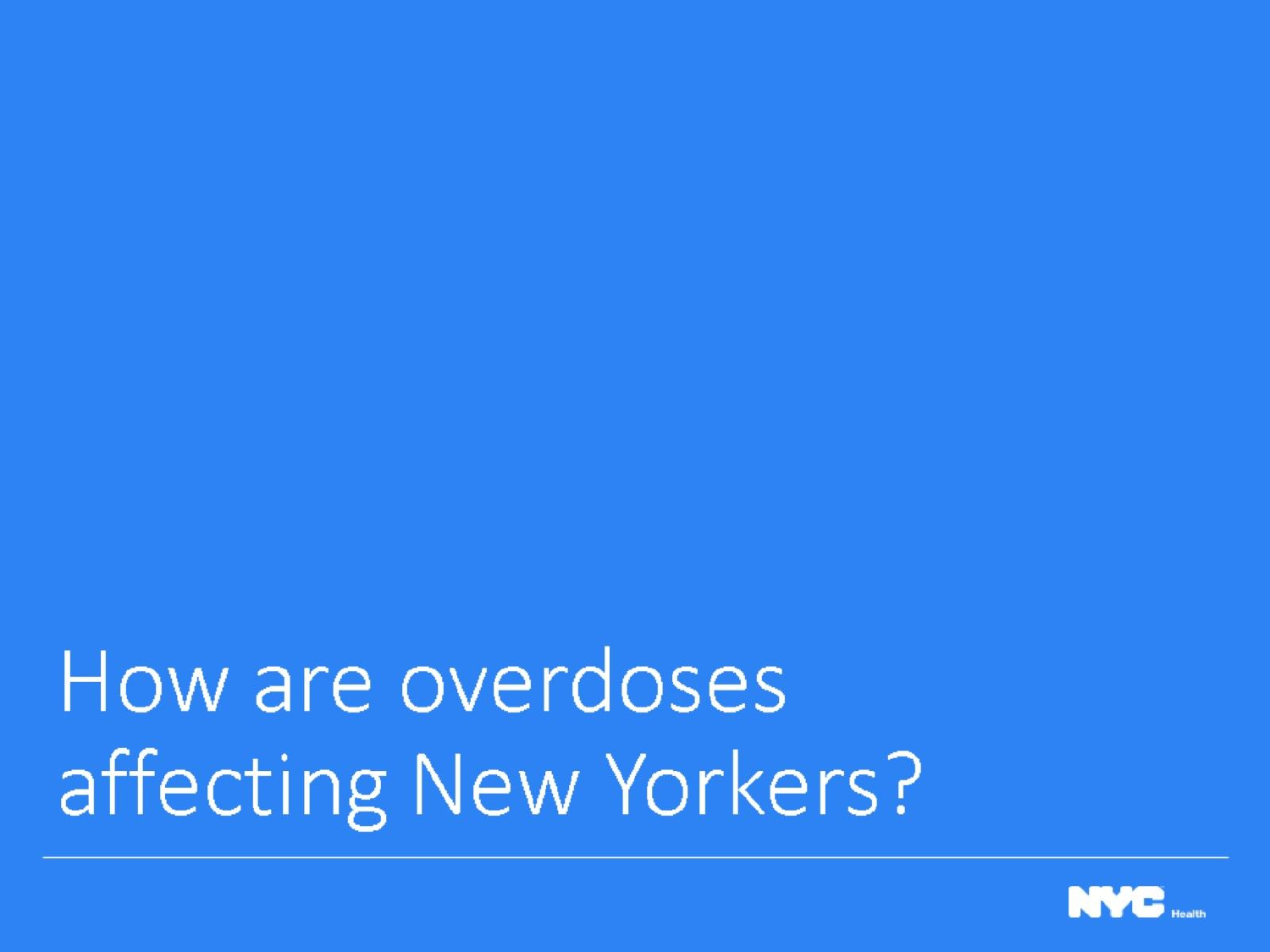

# How are overdoses affecting New Yorkers?

## Slide 8
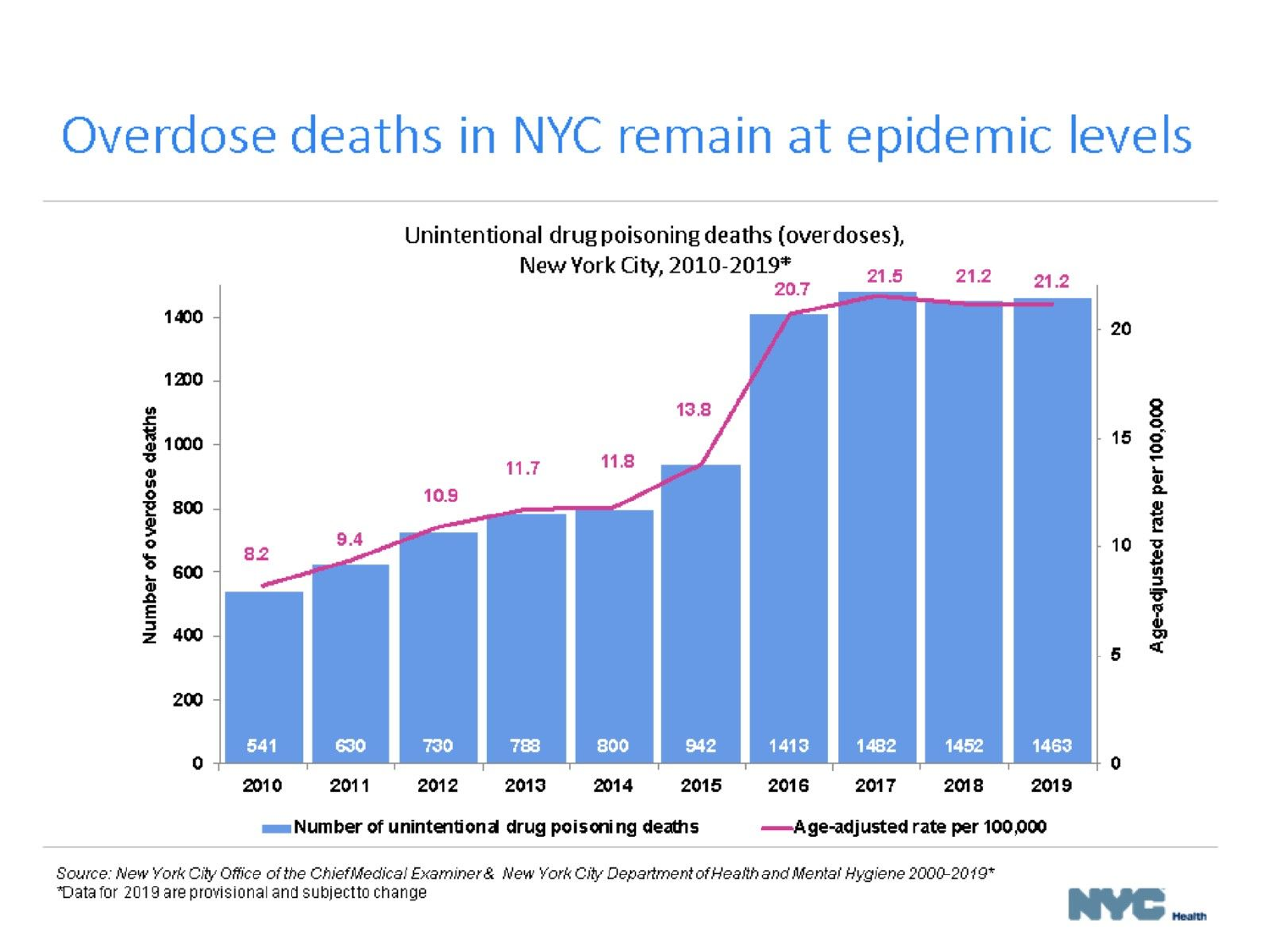

#

## Slide 9
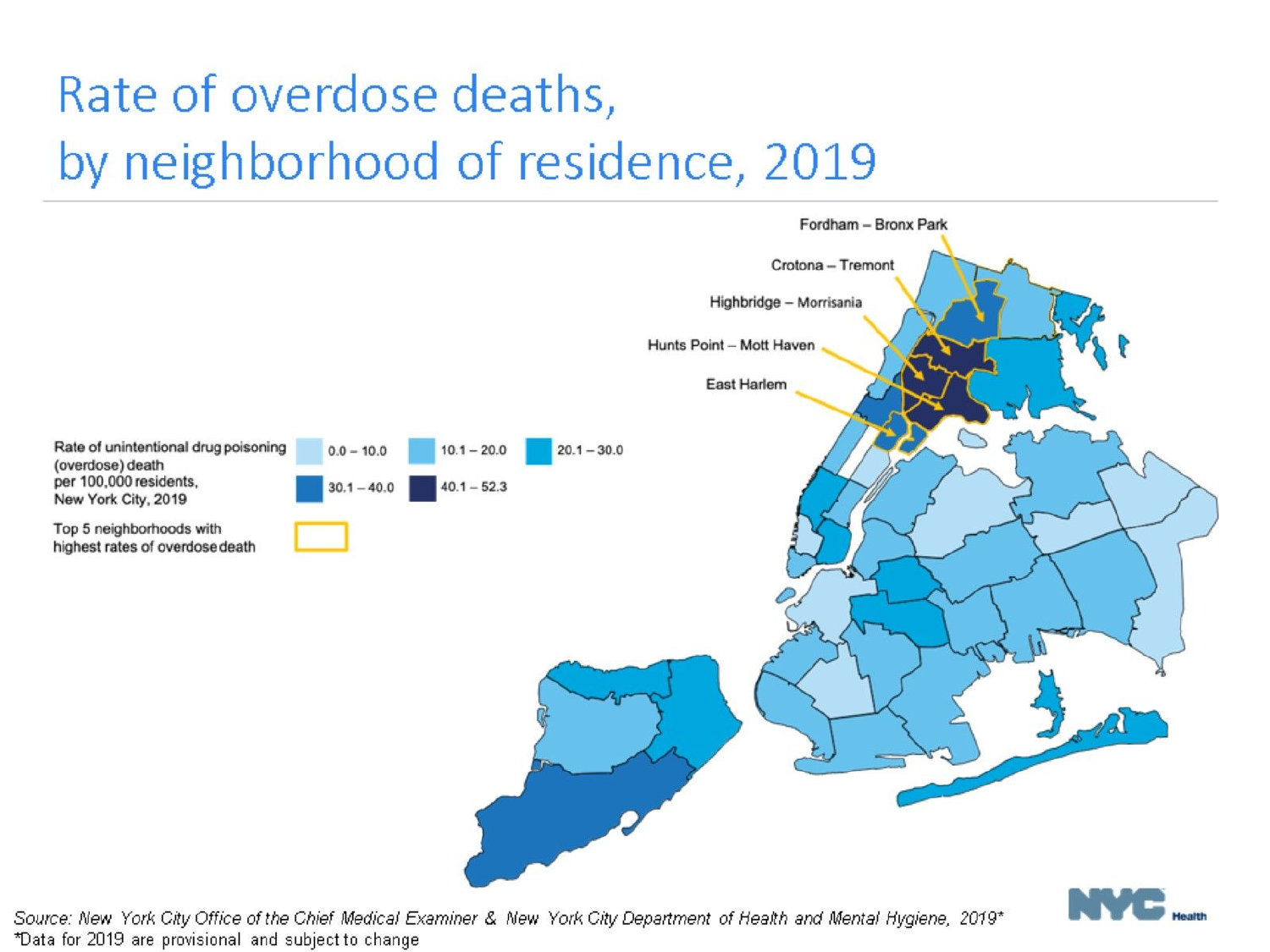

#

## Slide 10
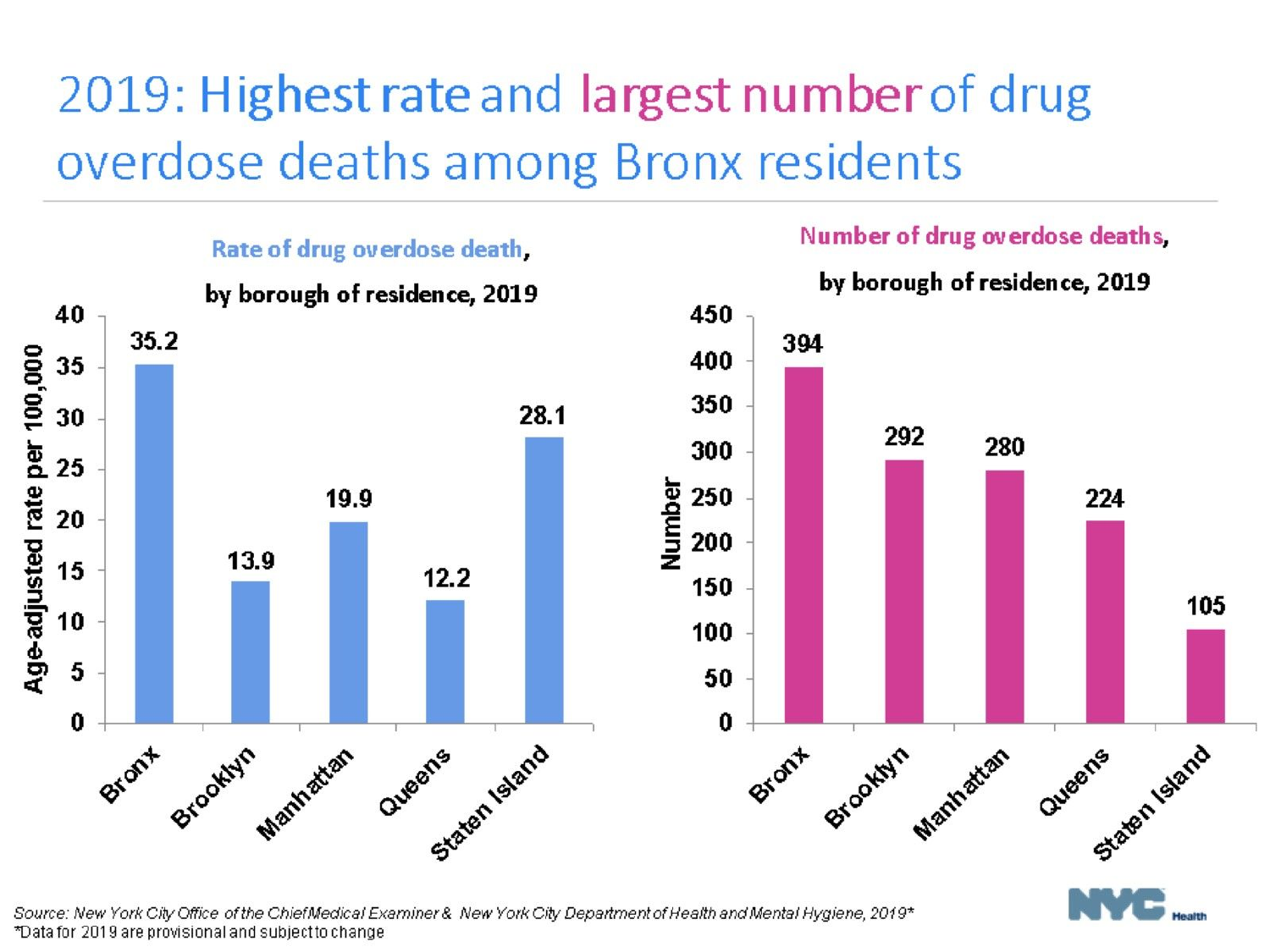

#

## Slide 11
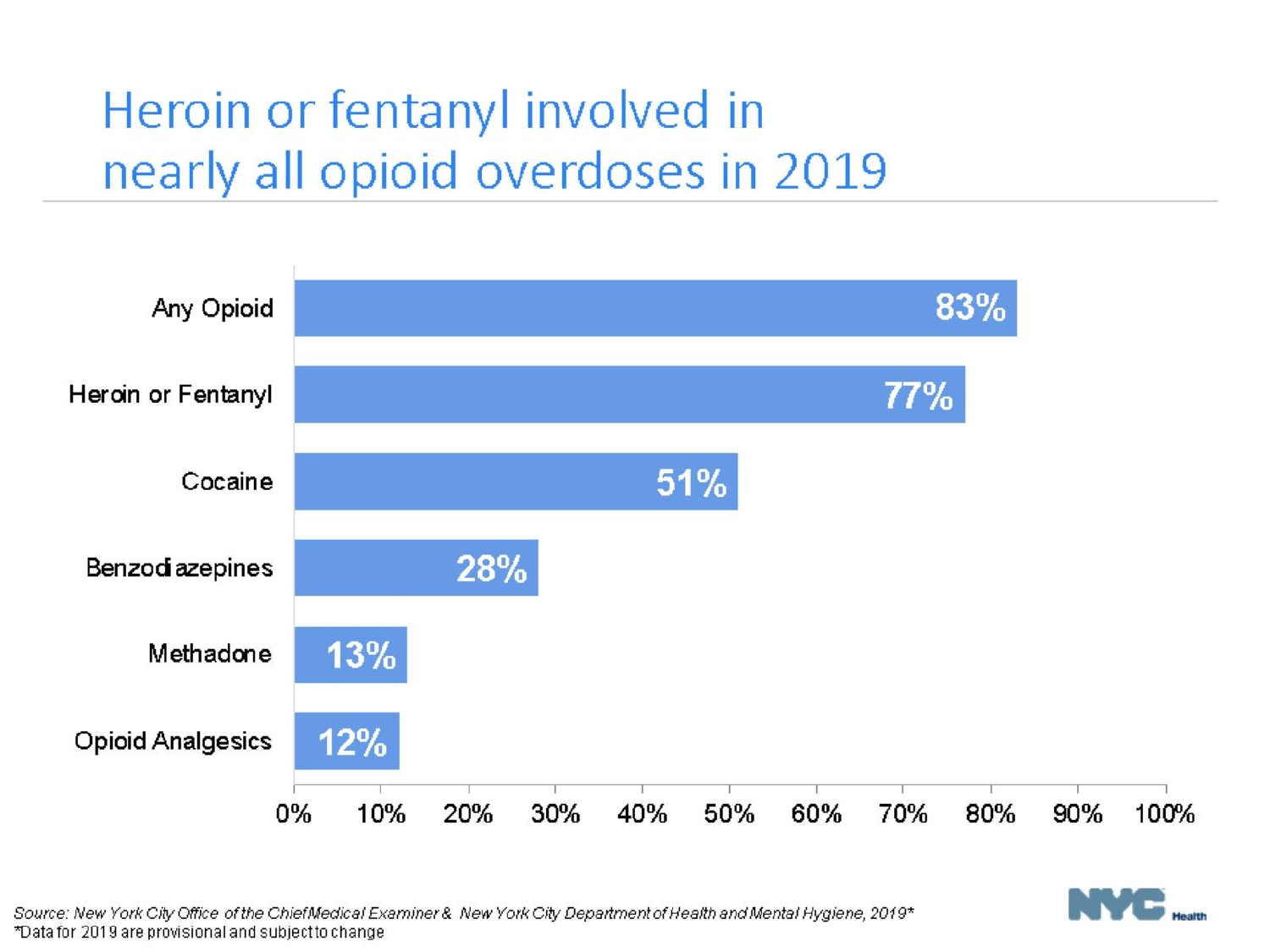

# Heroin or fentanyl involved in nearly all opioid overdoses in 2019

## Slide 12
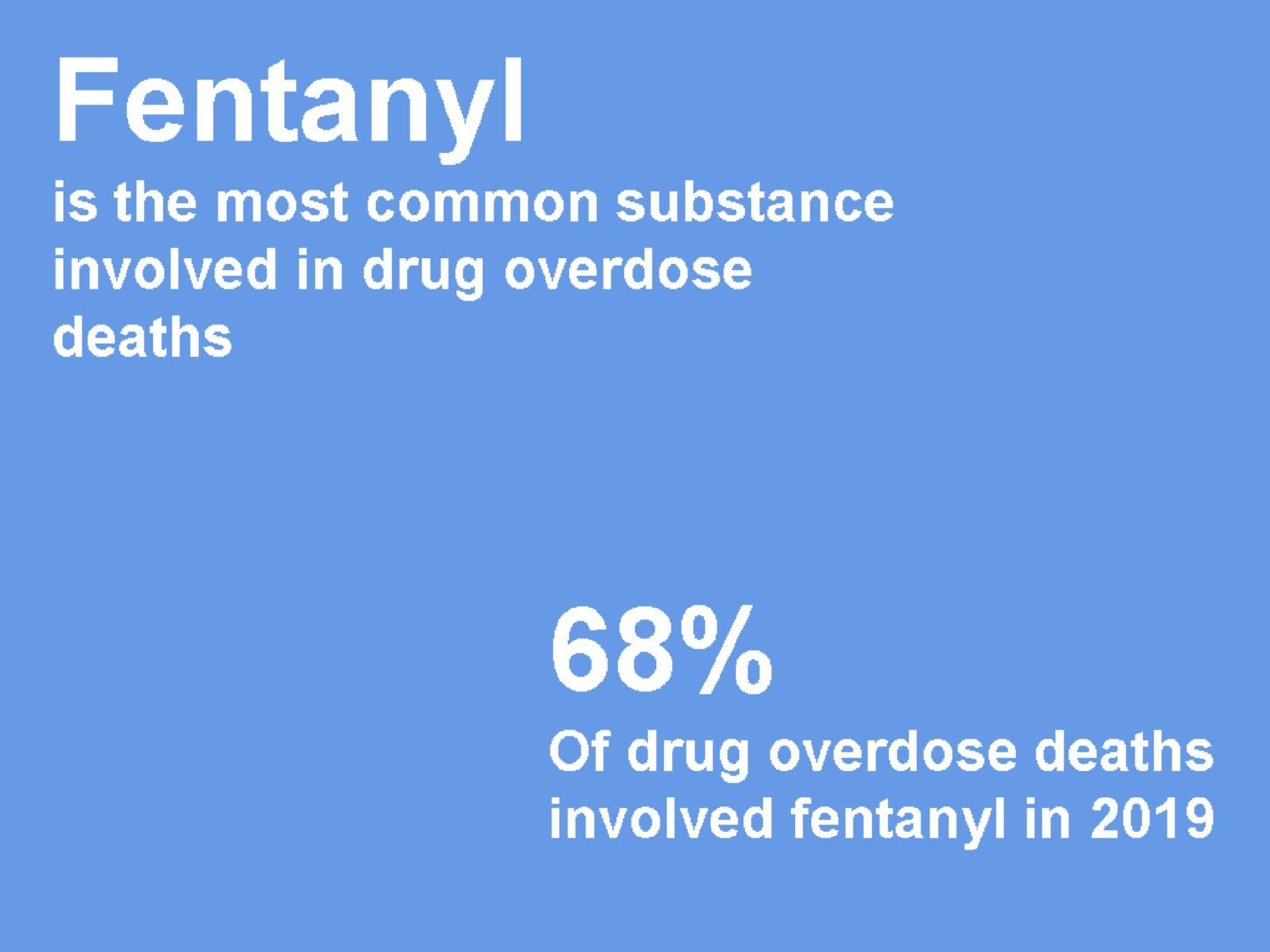

#

## Slide 13
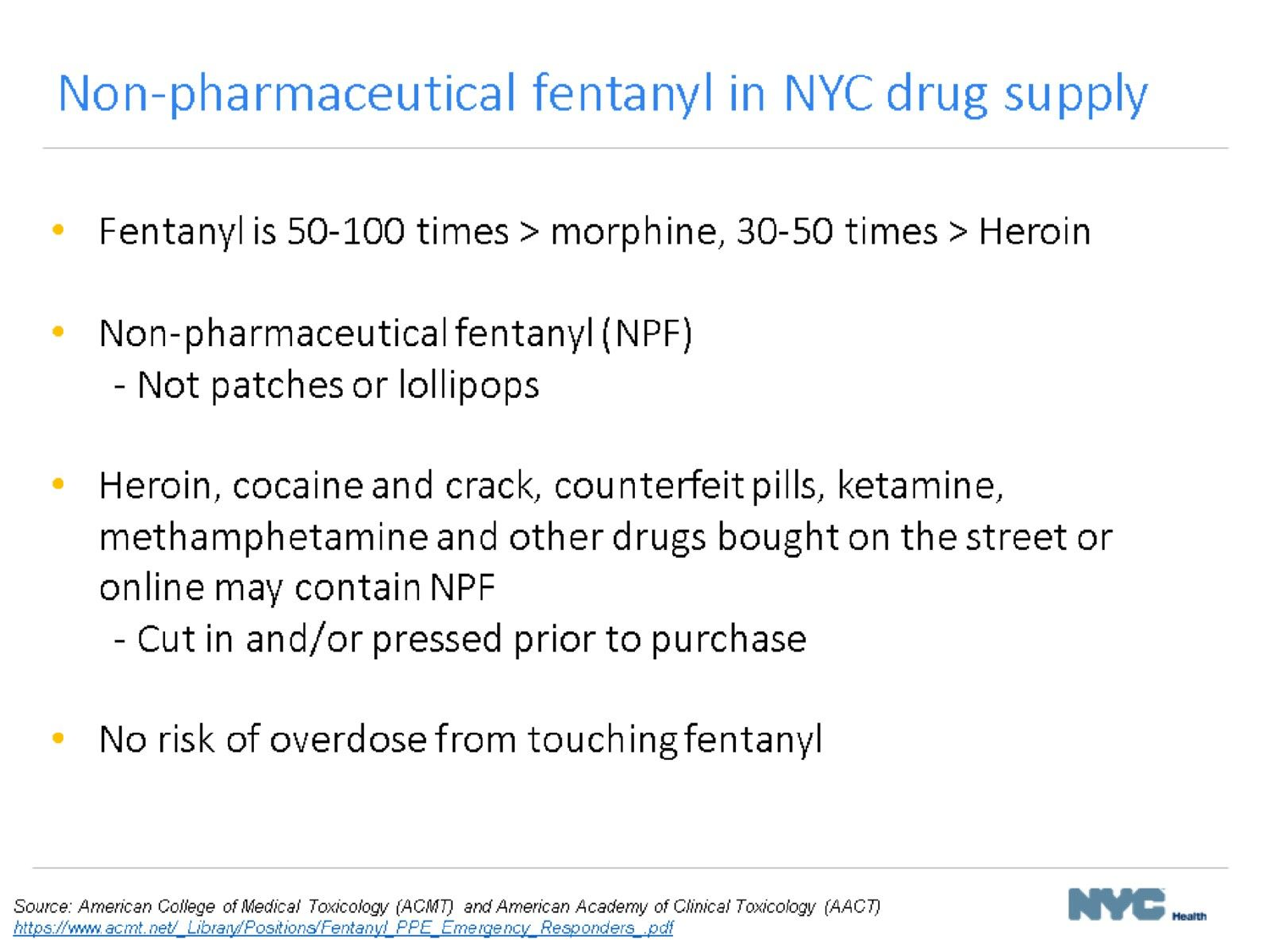

#

## Slide 14
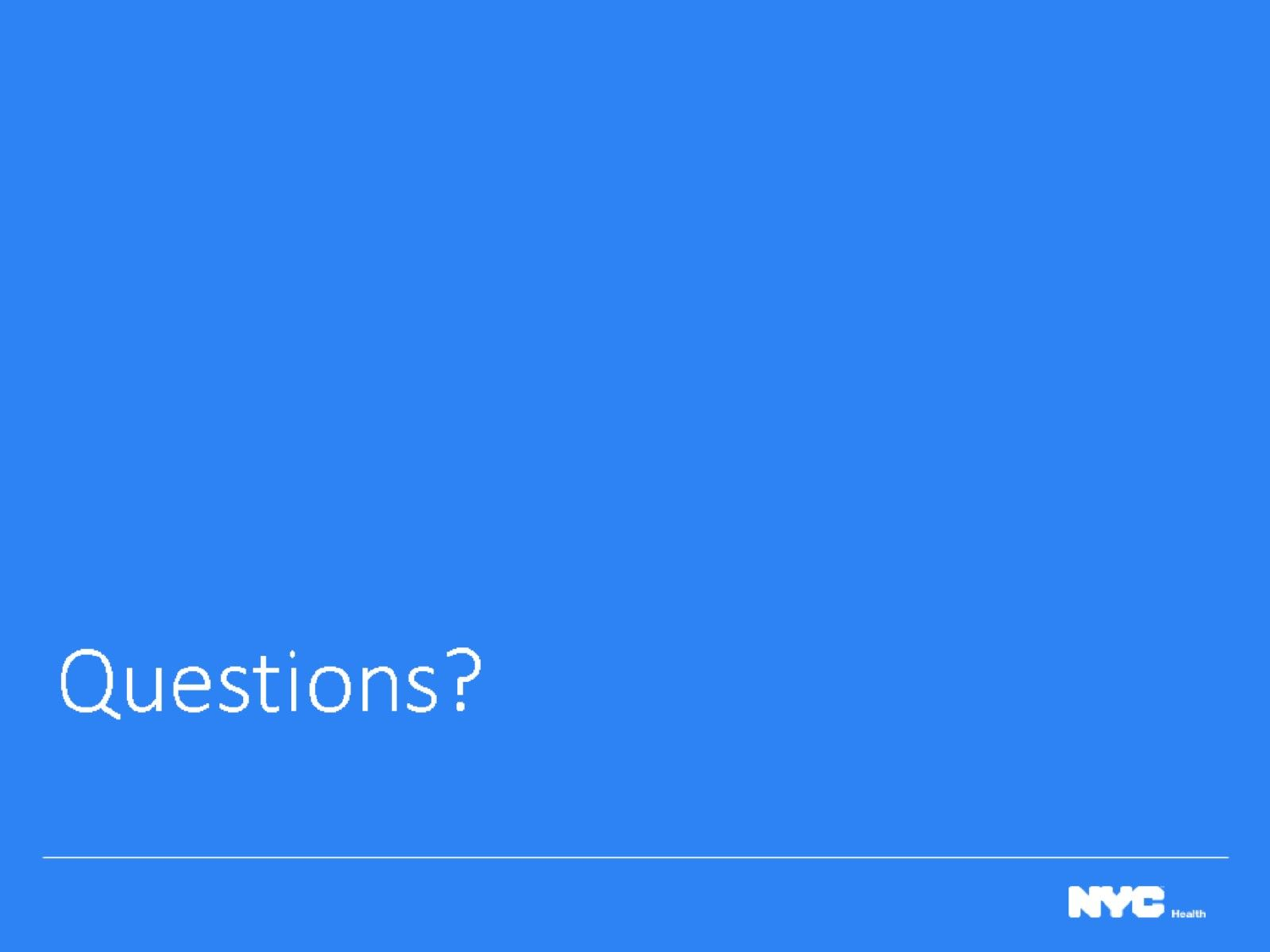

# Questions?

## Slide 15
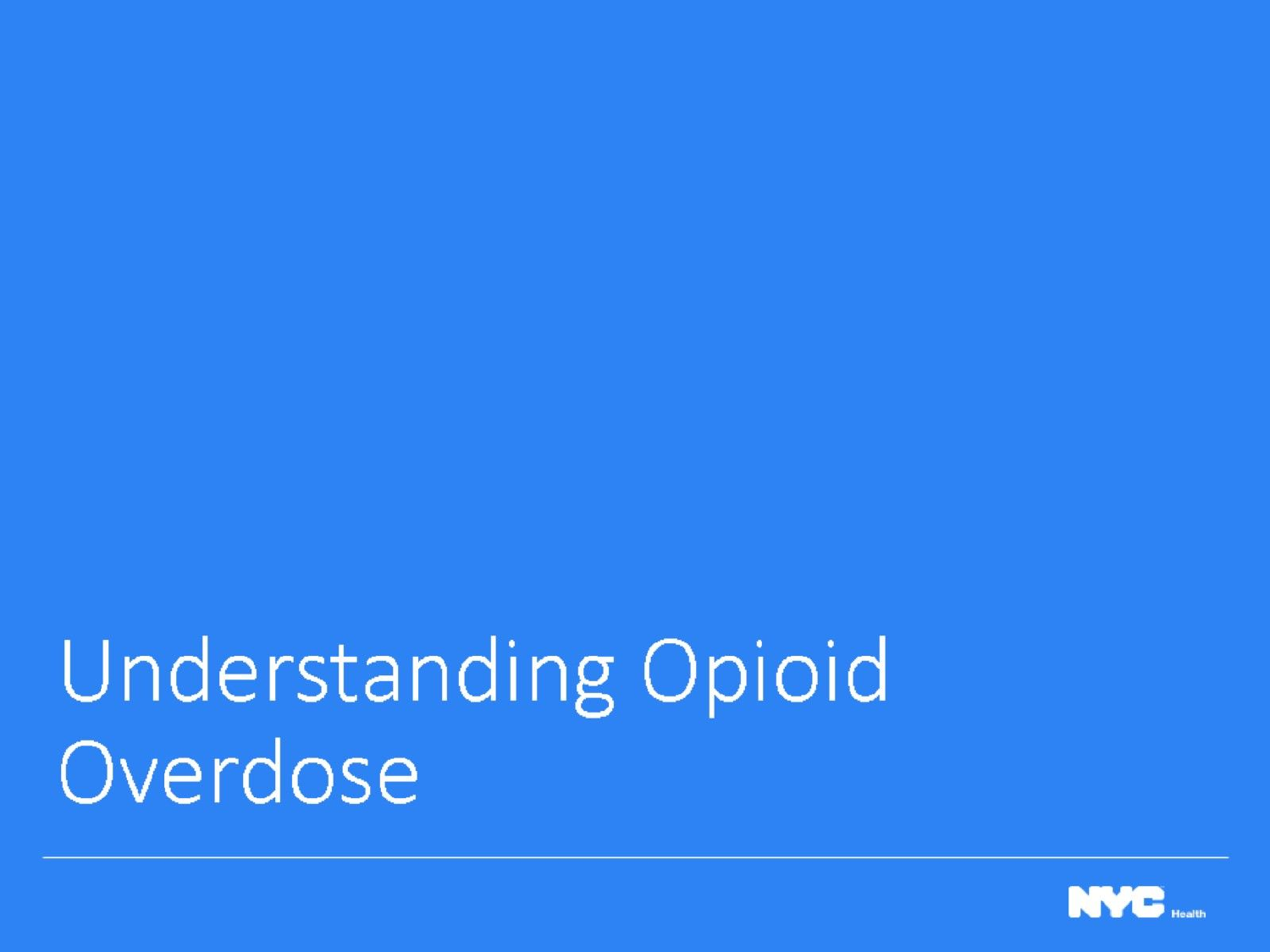

# Understanding Opioid Overdose

## Slide 16
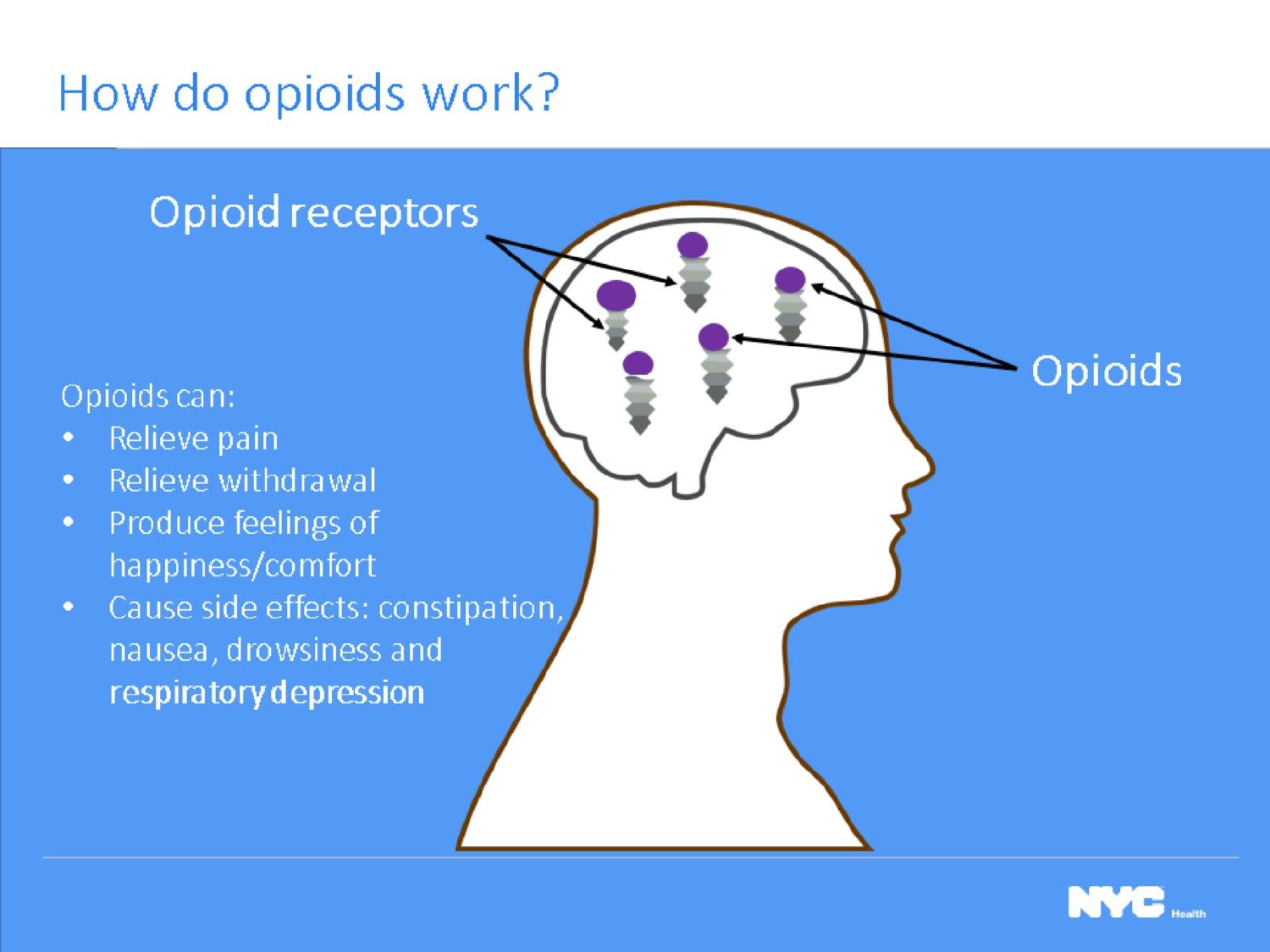

#

## Slide 17
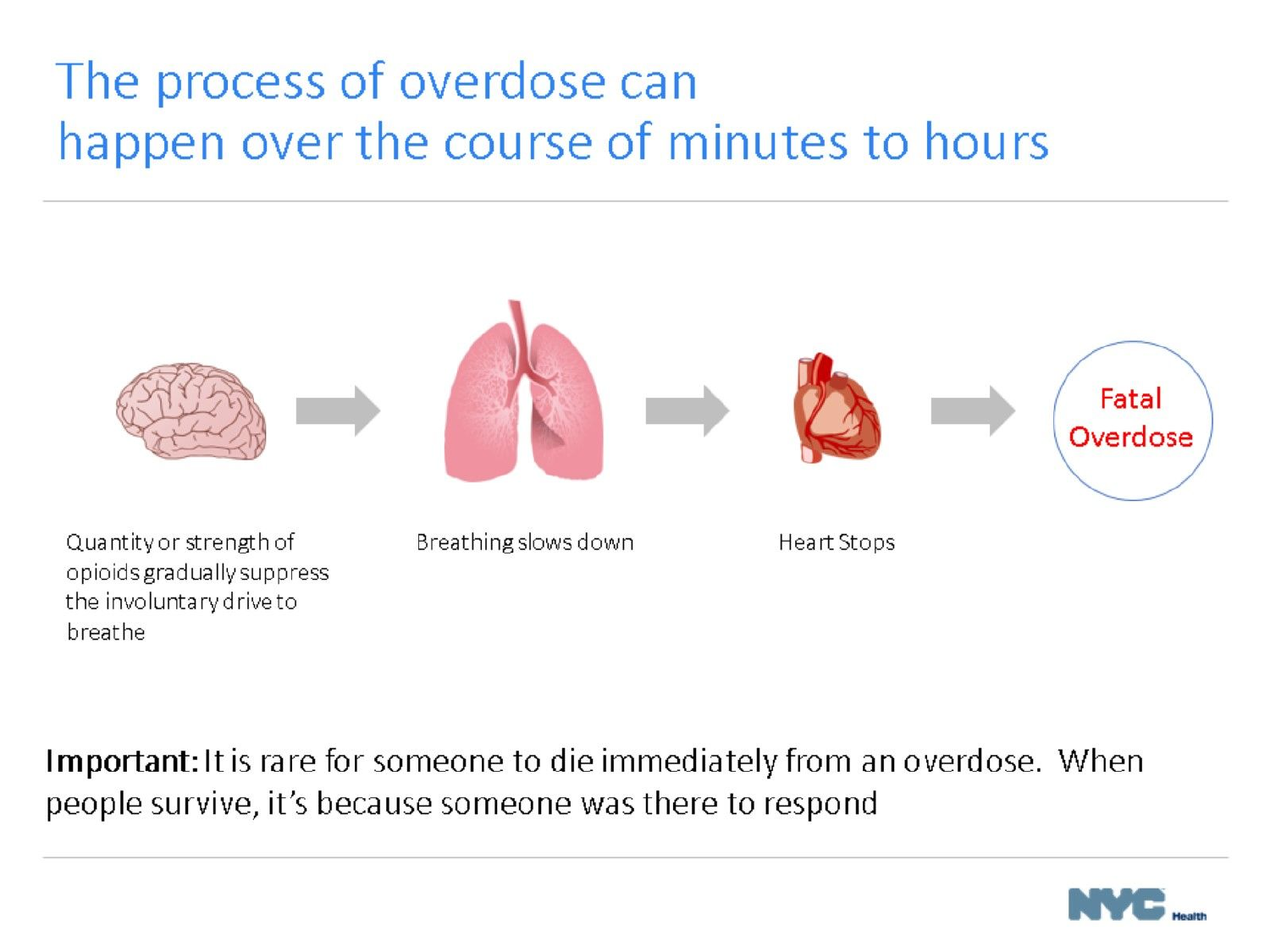

# The process of overdose can happen over the course of minutes to hours

## Slide 18
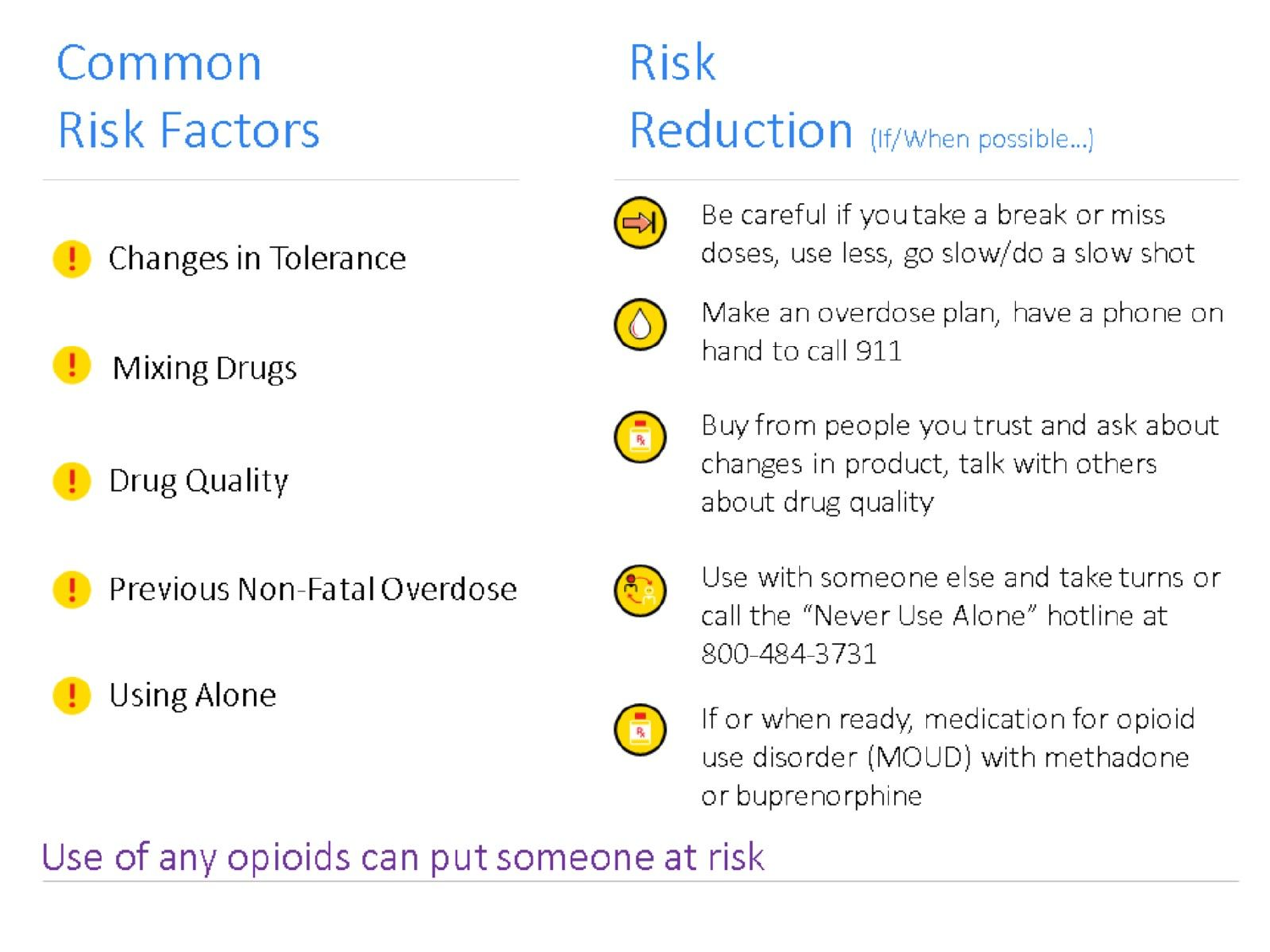

#

## Slide 19
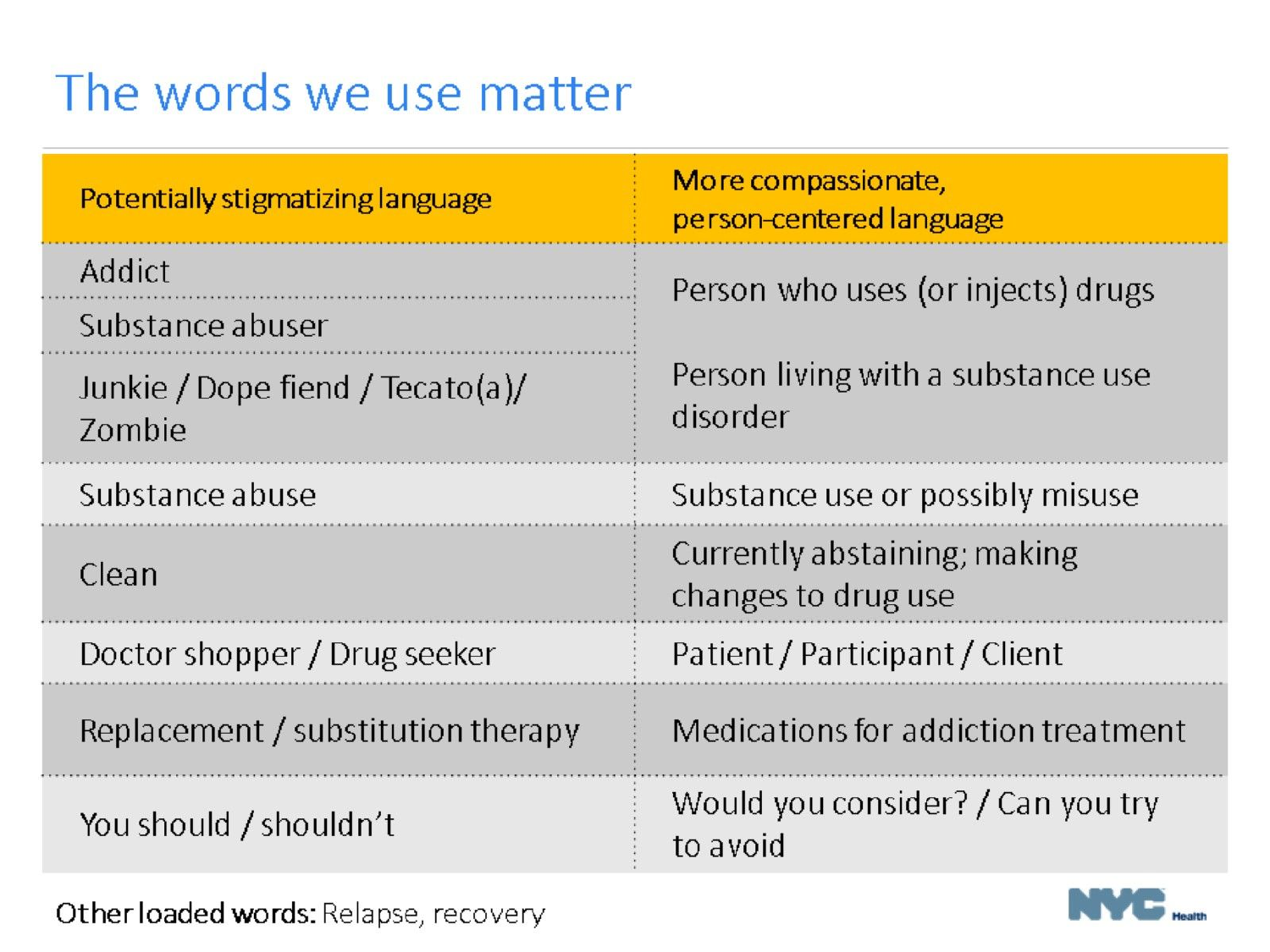

#

## Slide 20
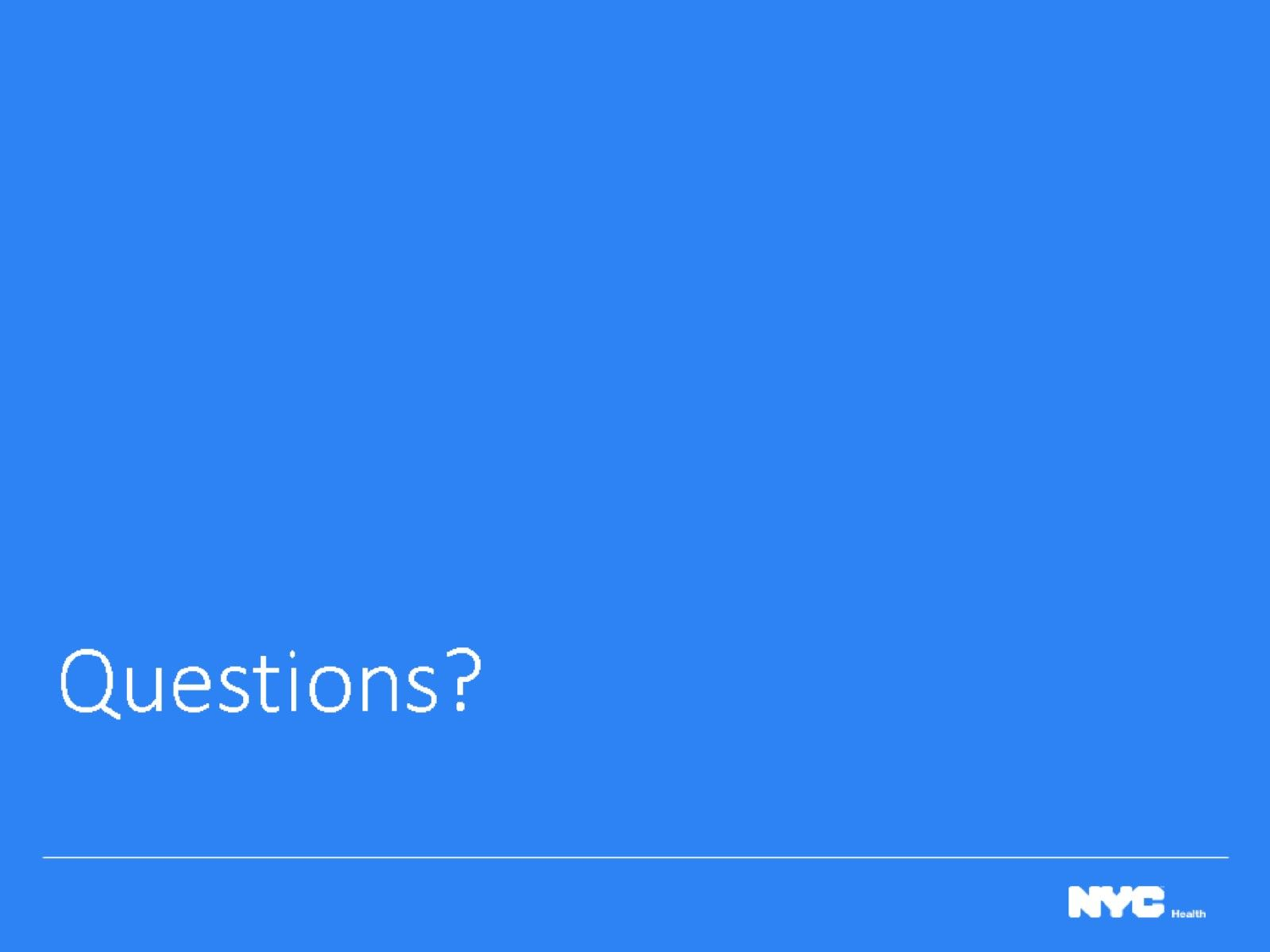

# Questions?

## Slide 21
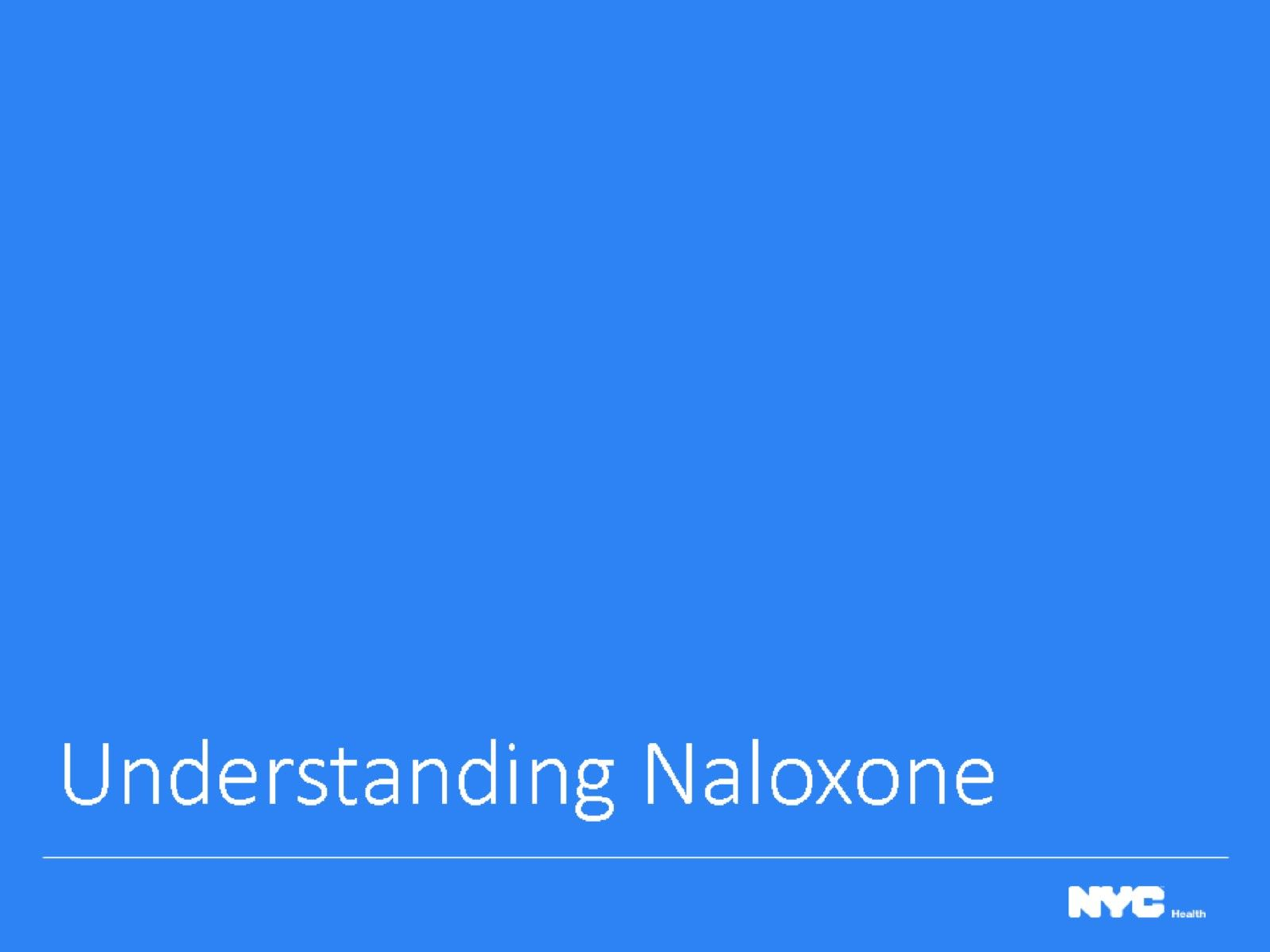

# Understanding Naloxone

## Slide 22
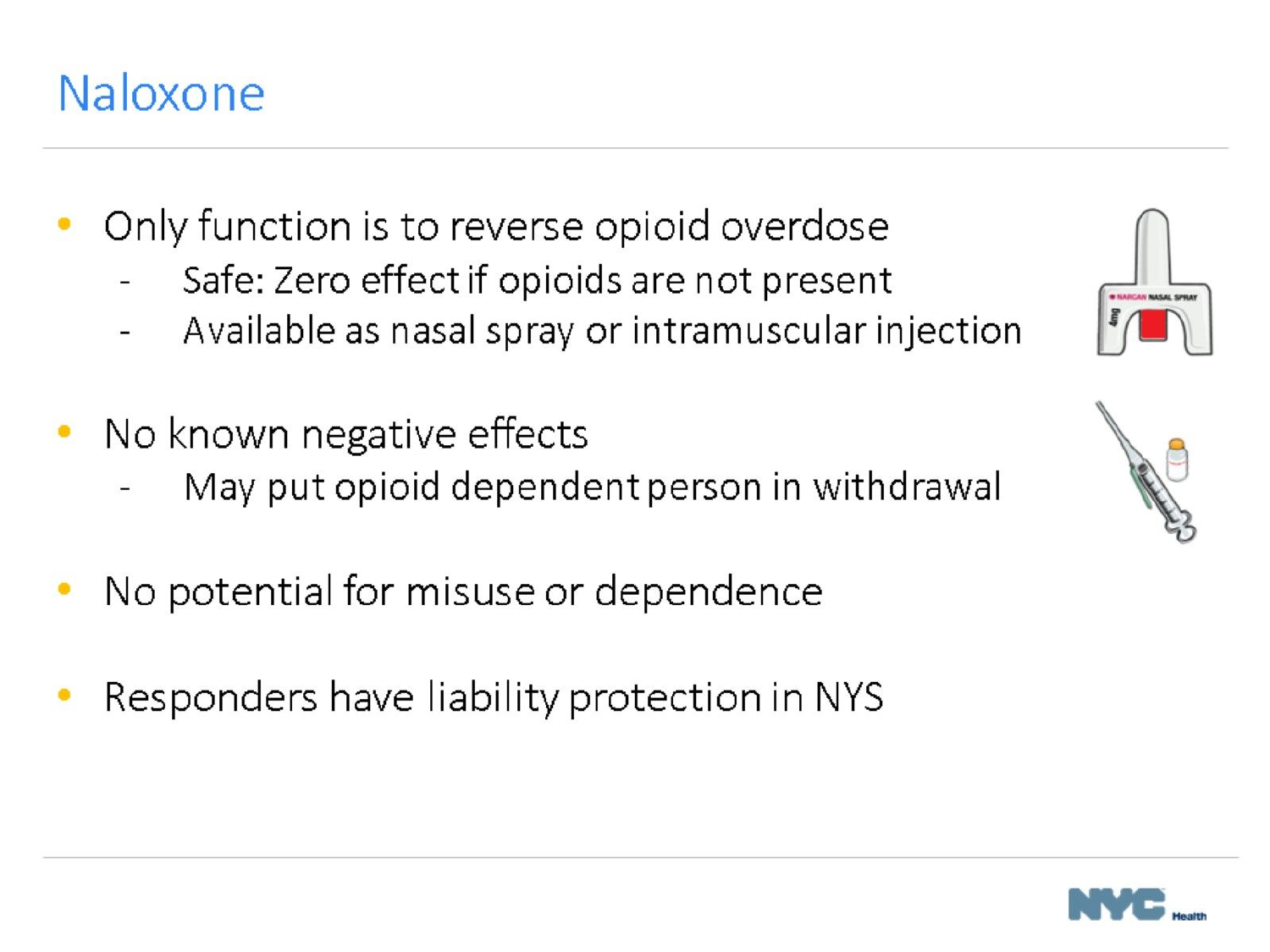

#

## Slide 23
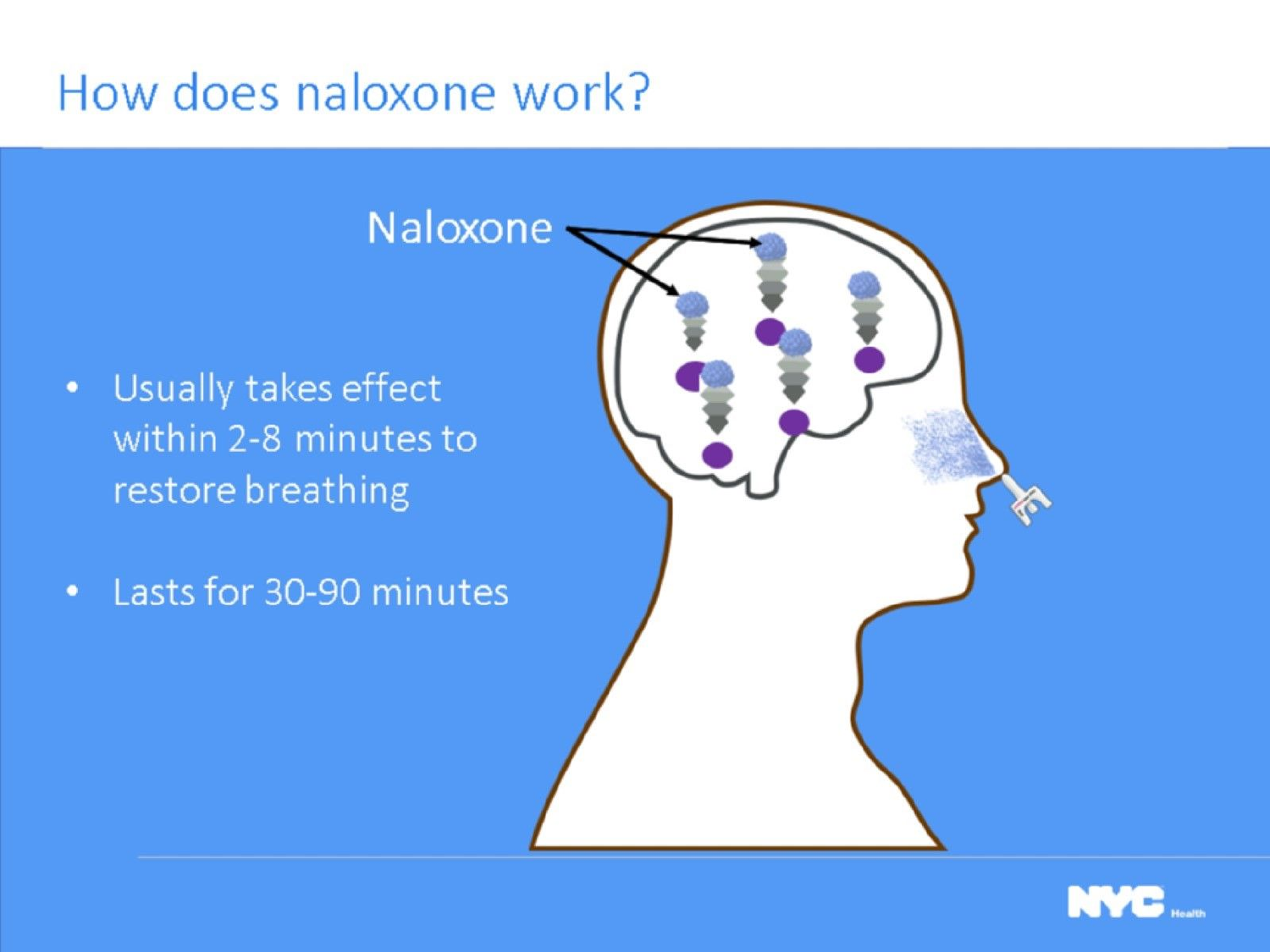

#

## Slide 24
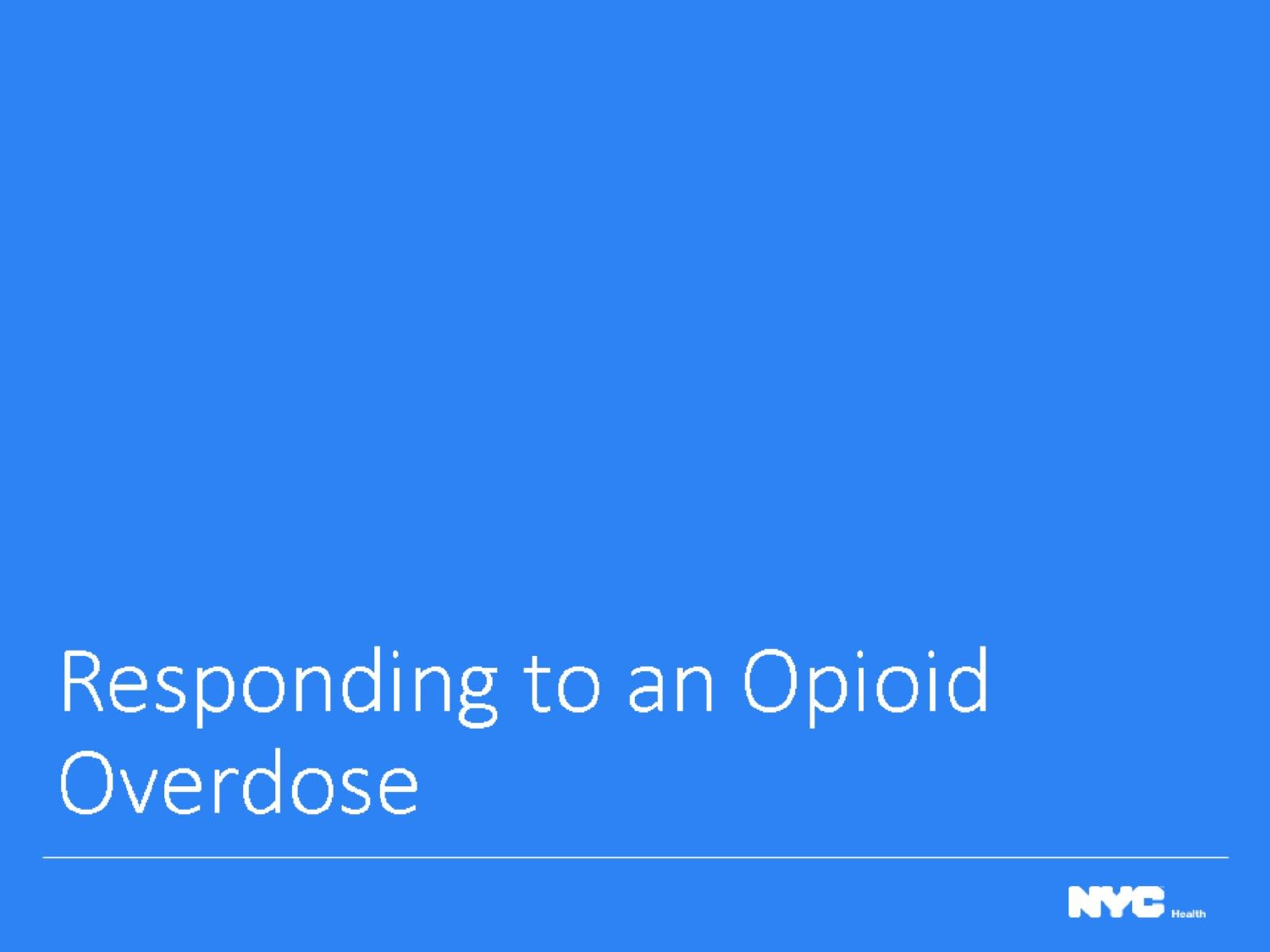

# Responding to an Opioid Overdose

## Slide 25
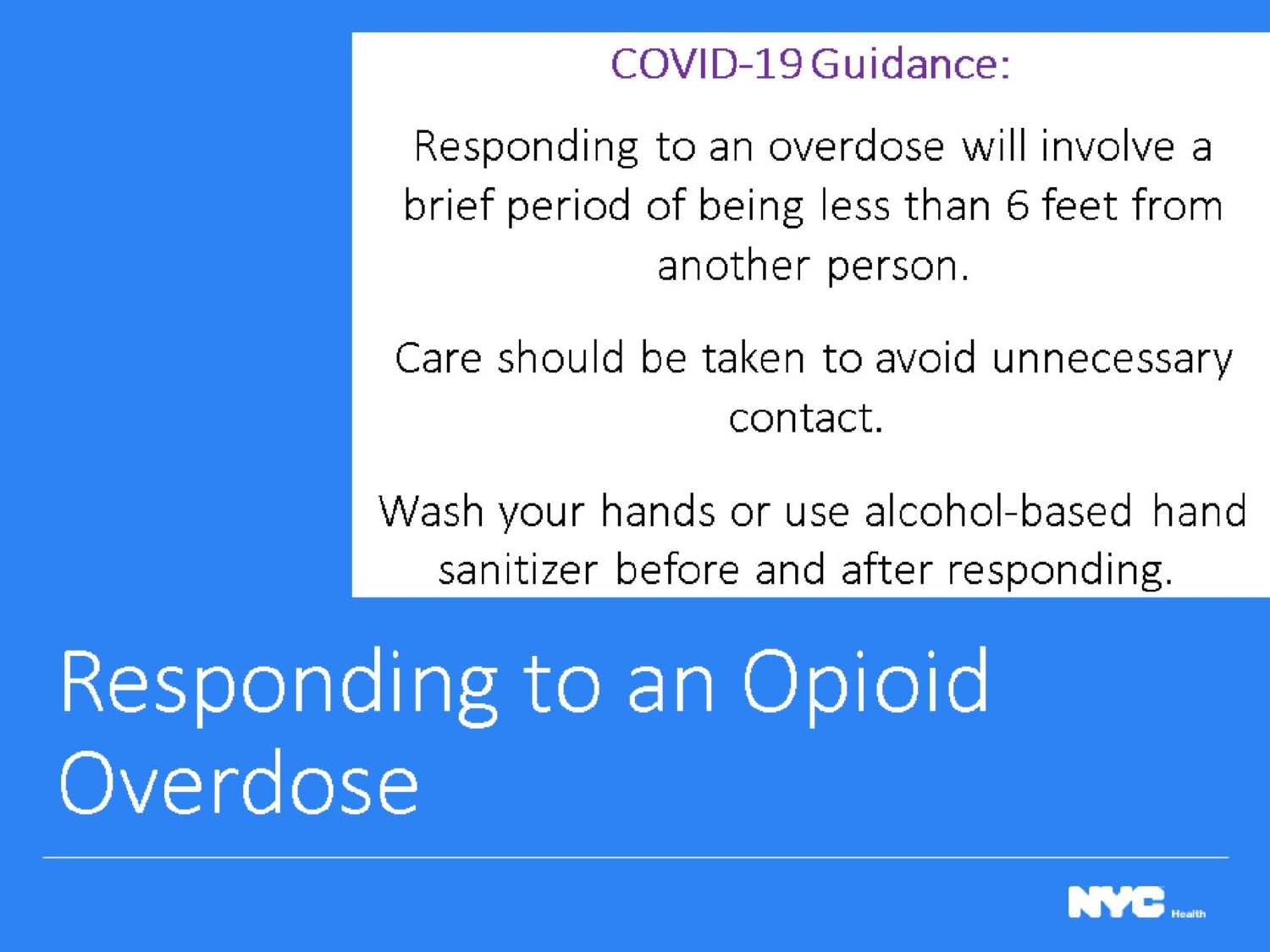

# Responding to an Opioid Overdose

## Slide 26
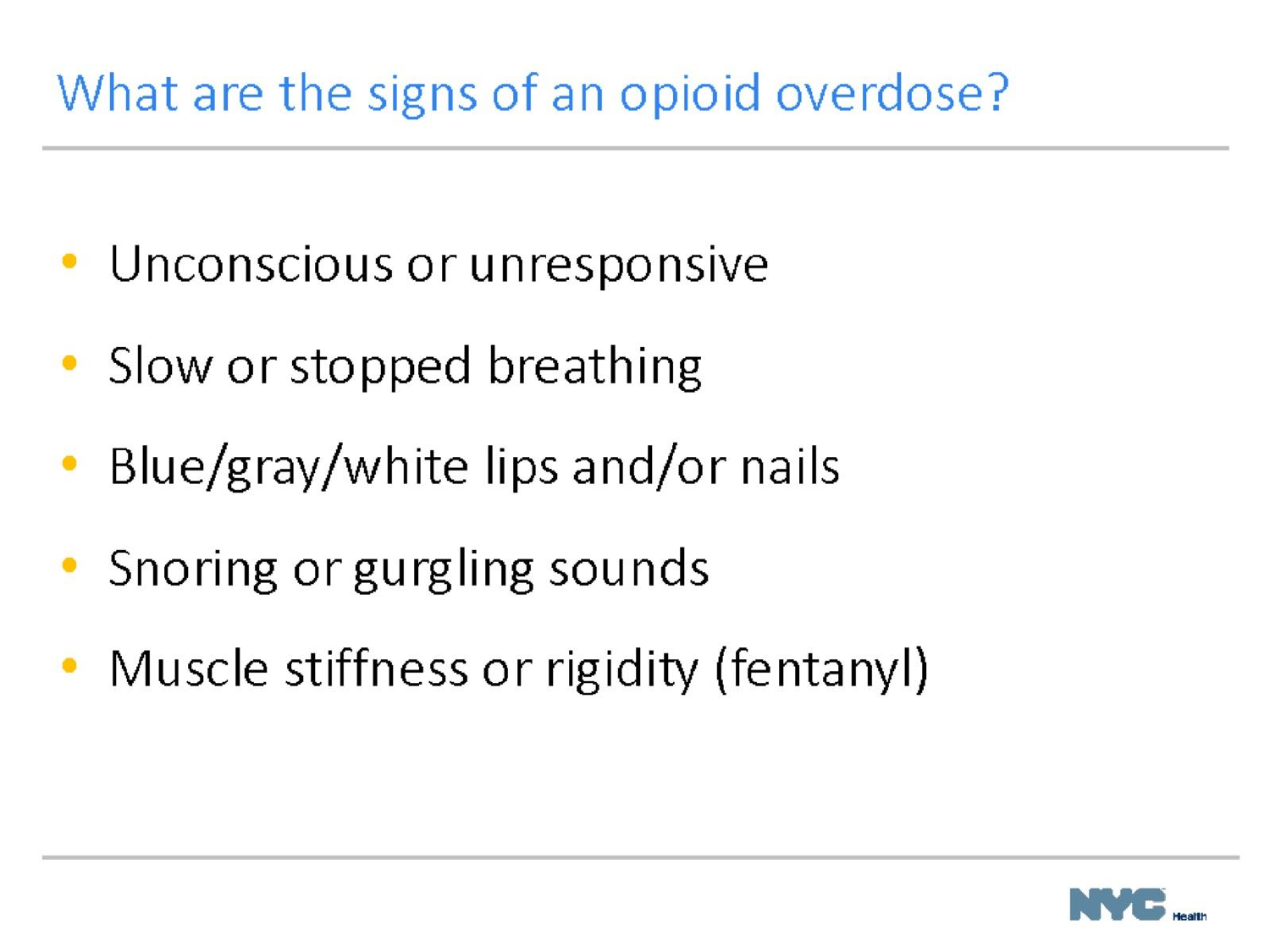

#

## Slide 27
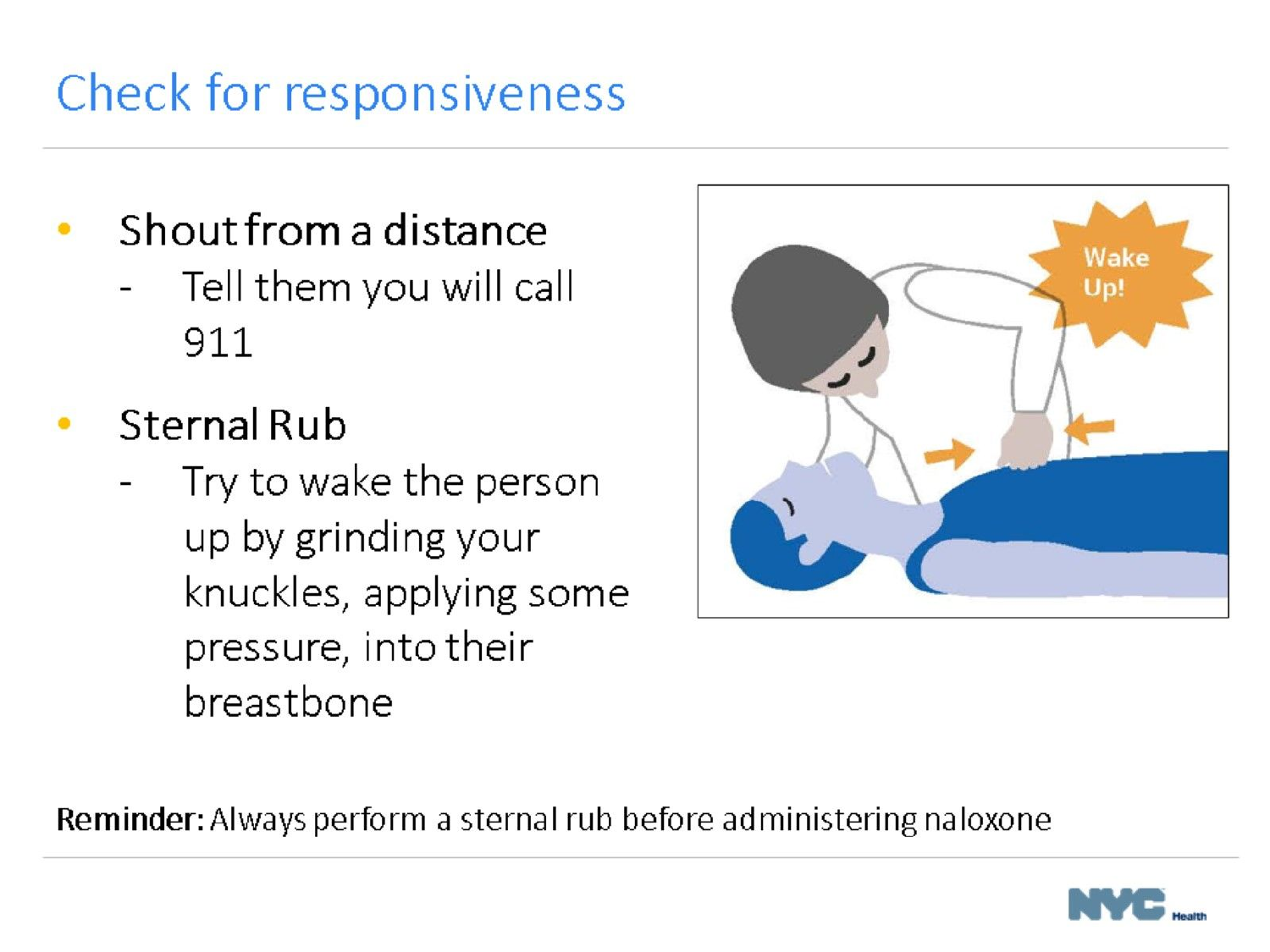

#

## Slide 28
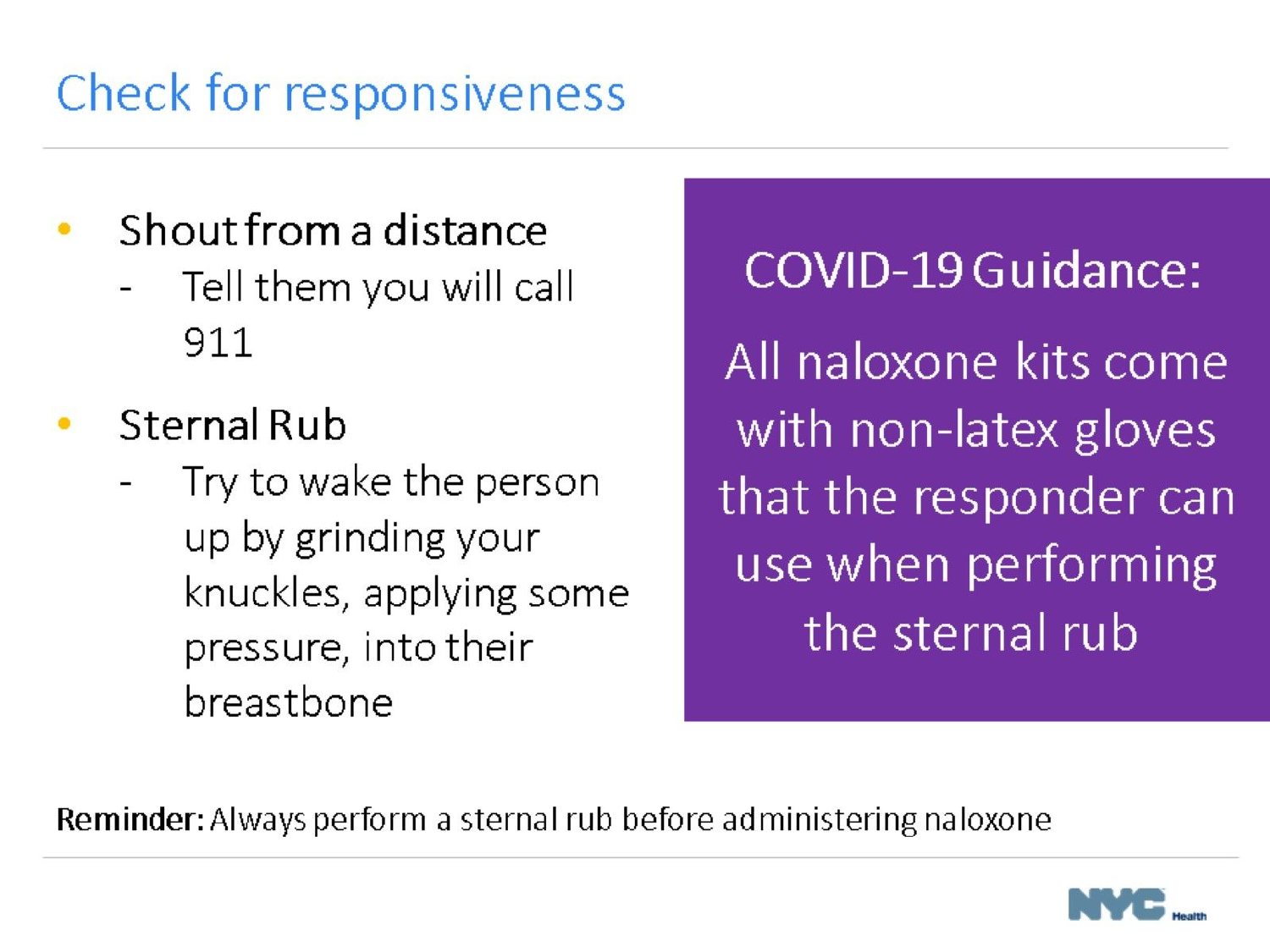

#

## Slide 29
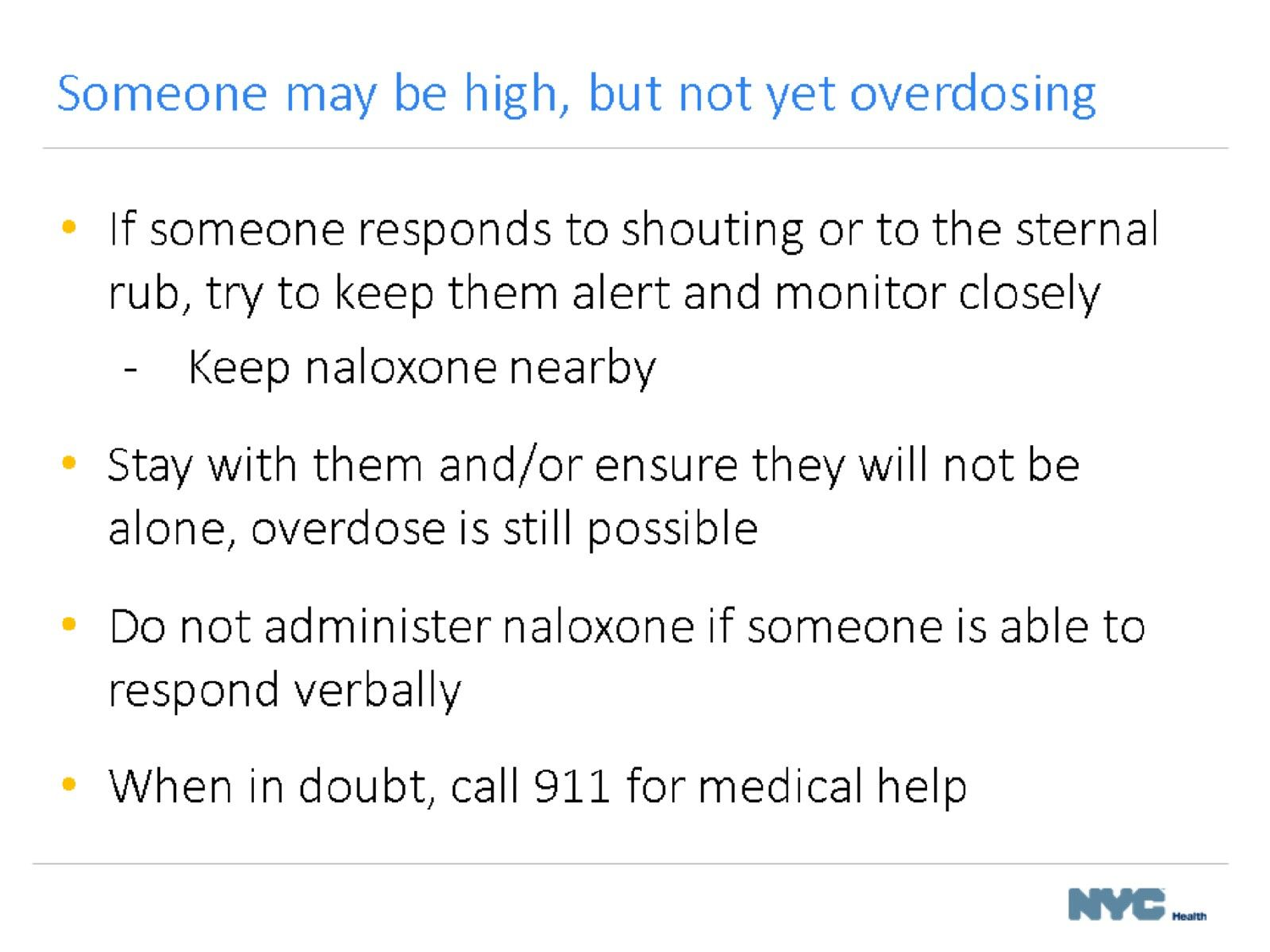

#

## Slide 30
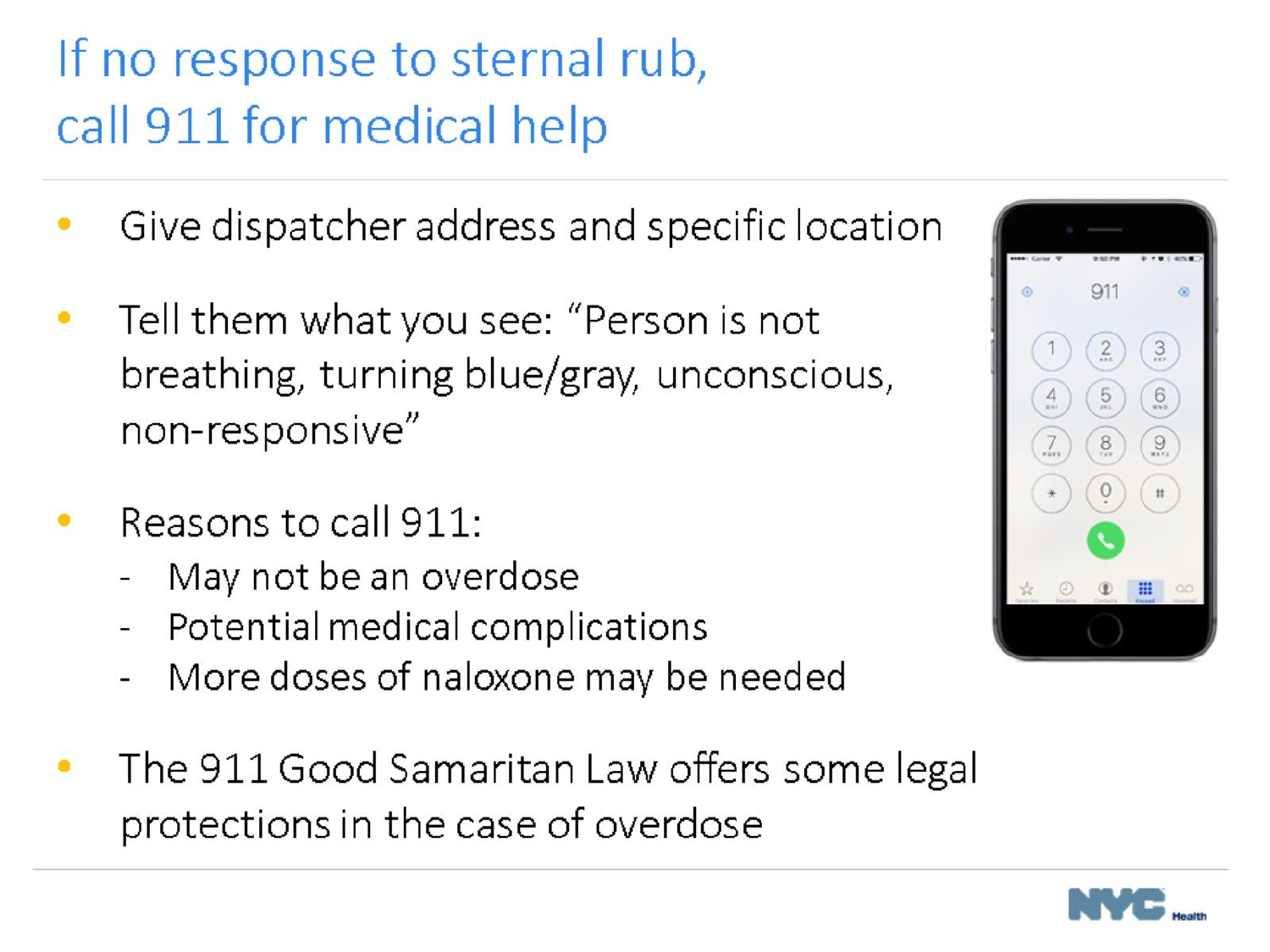

#

## Slide 31
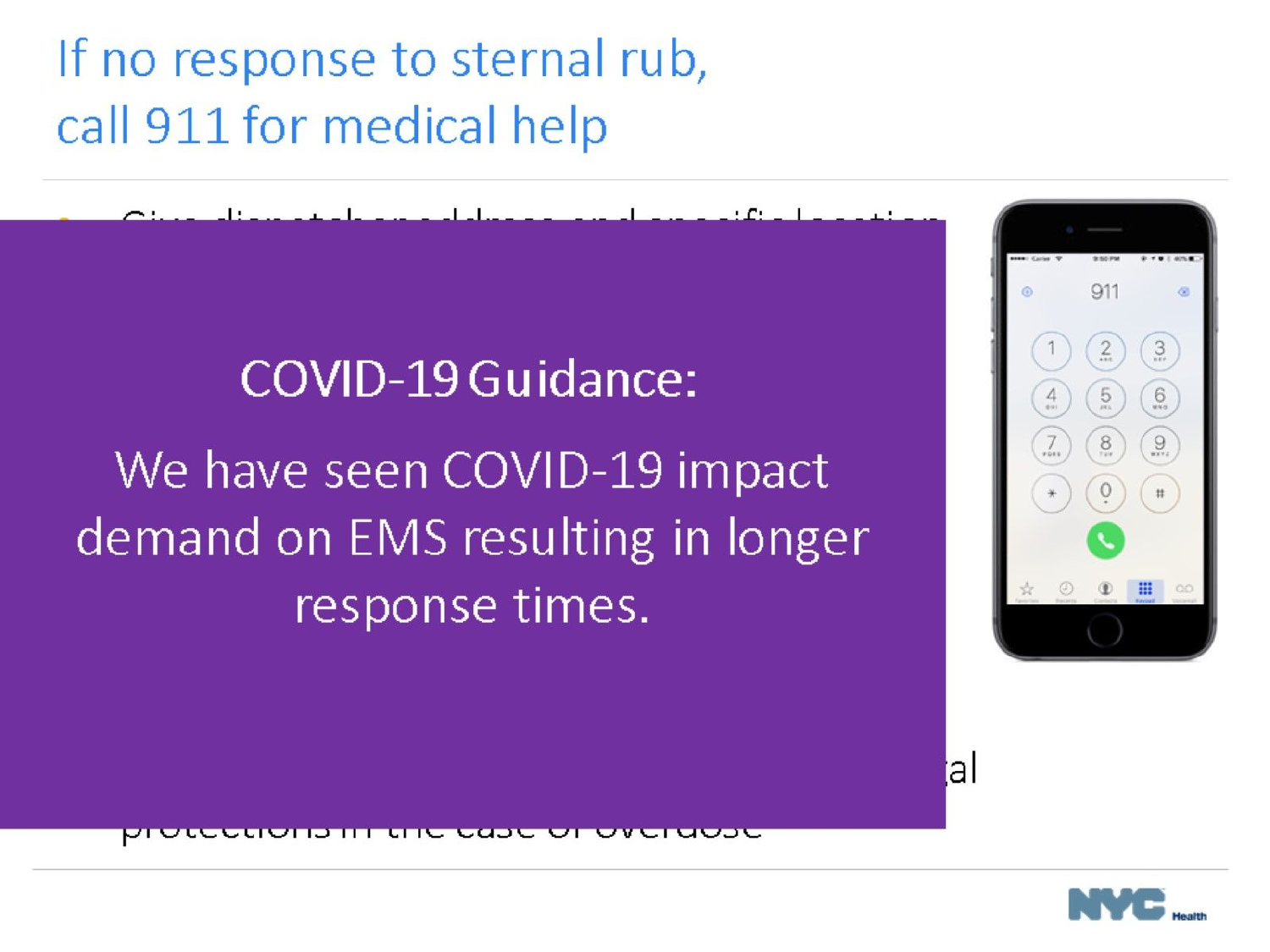

#

## Slide 32
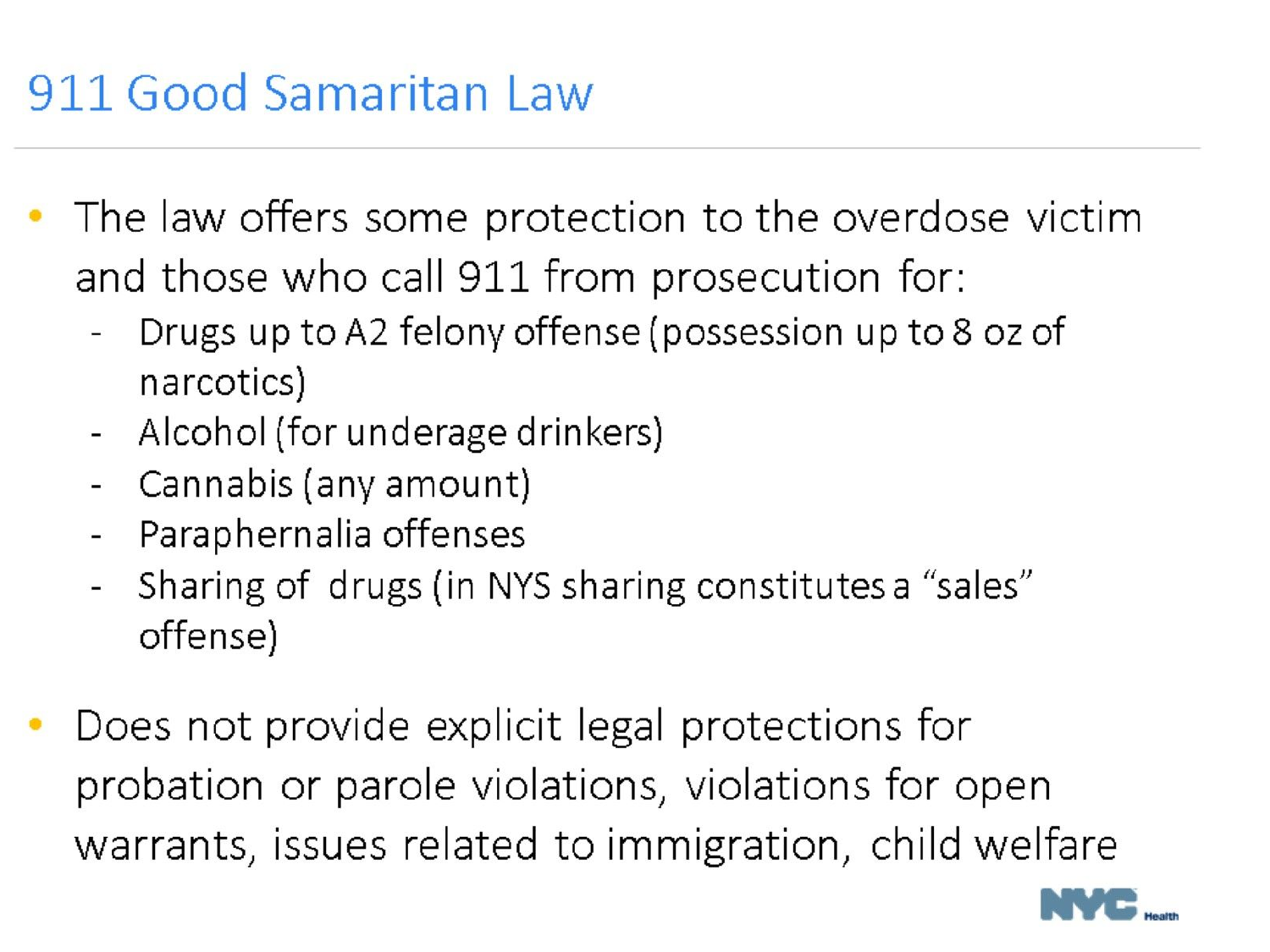

#

## Slide 33
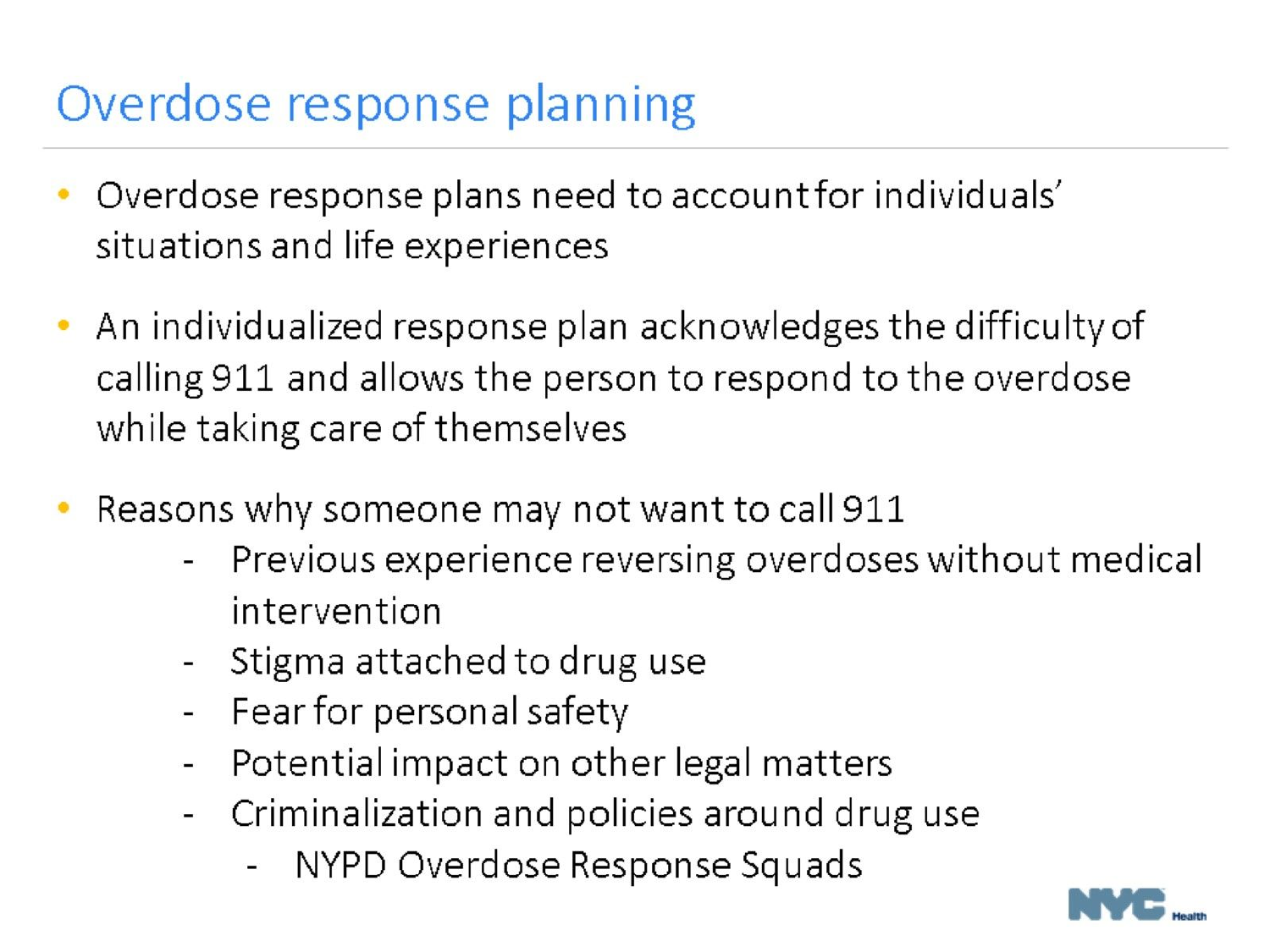

#

## Slide 34
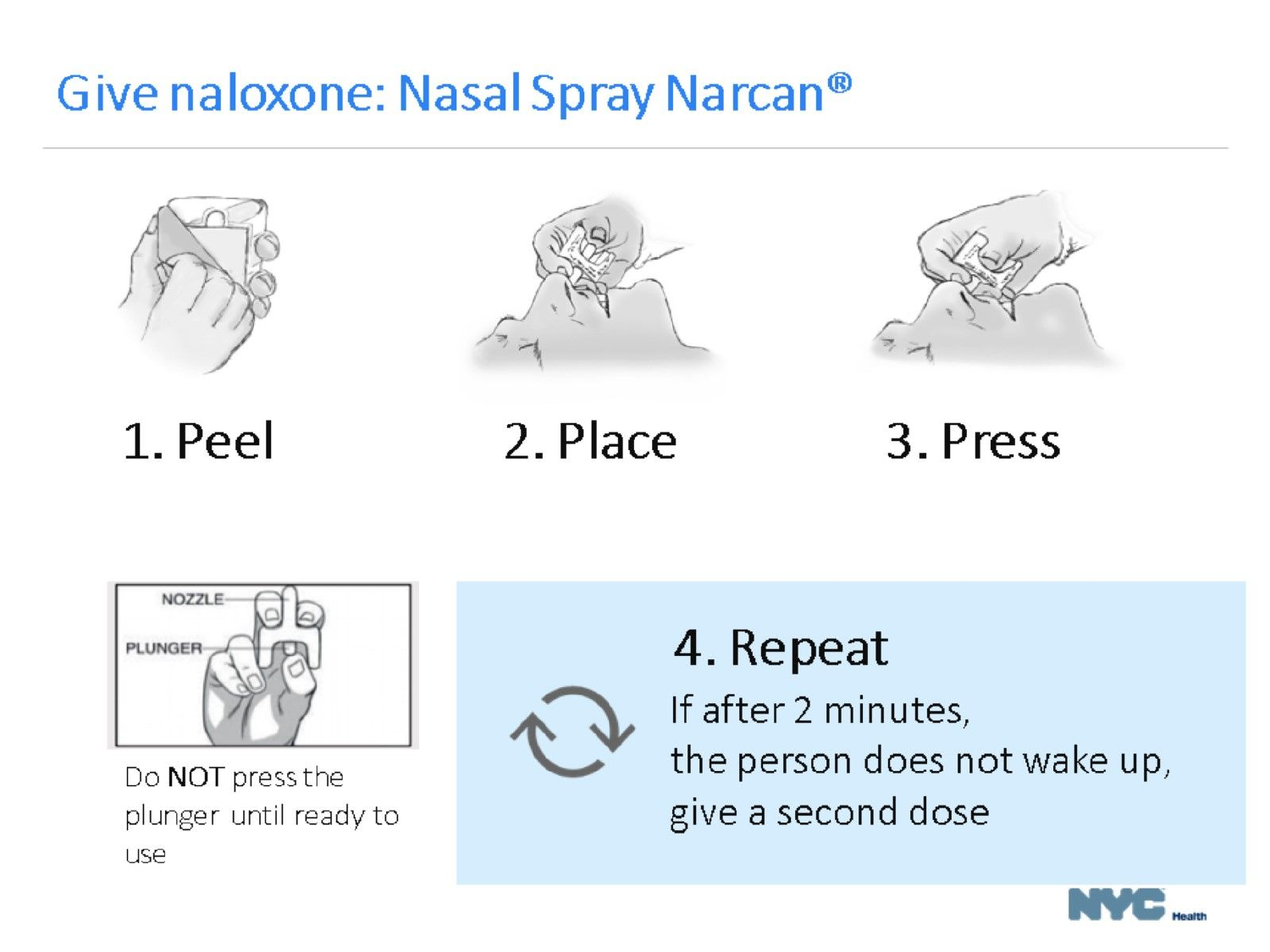

#

## Slide 35
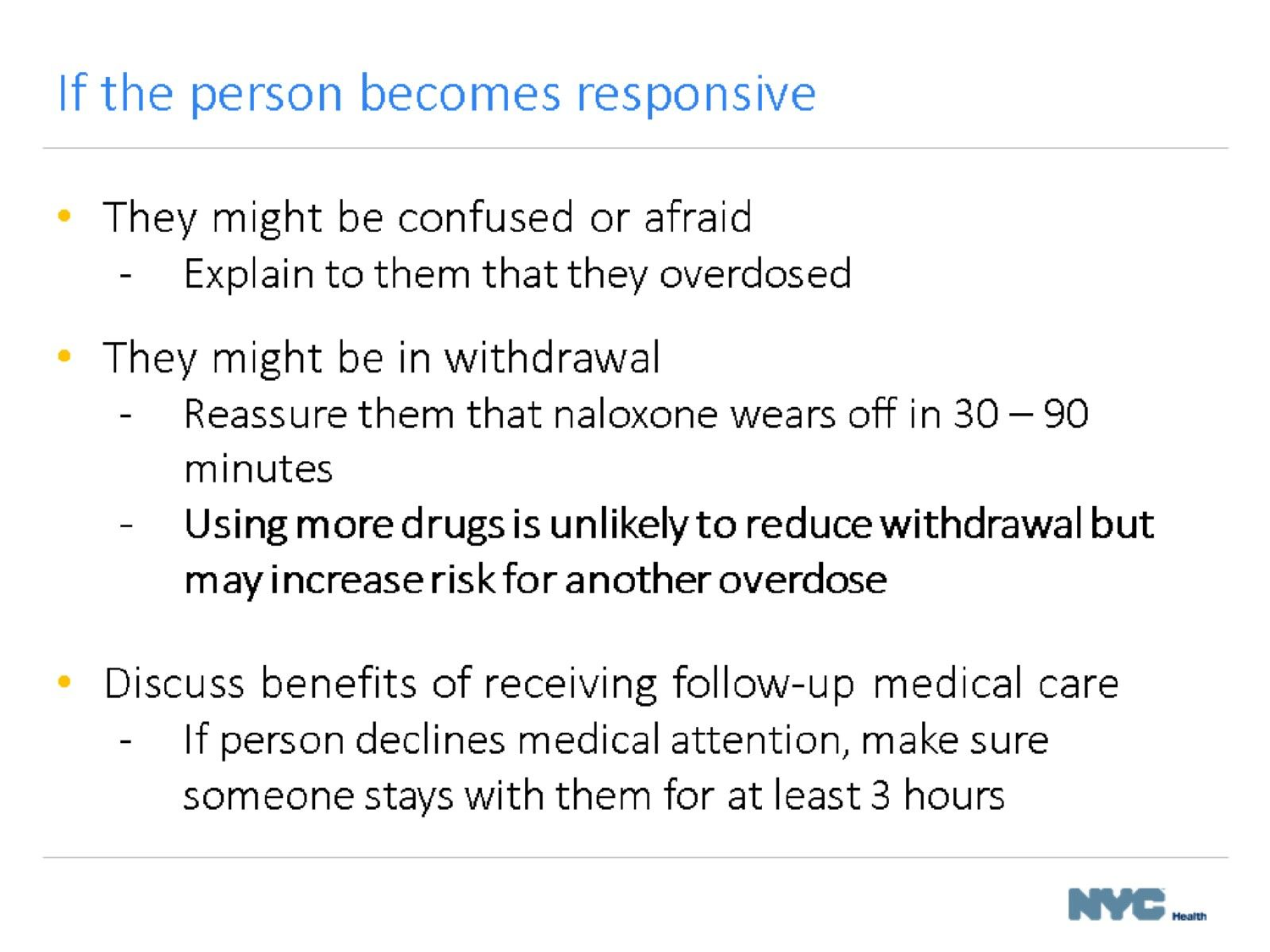

#

## Slide 36
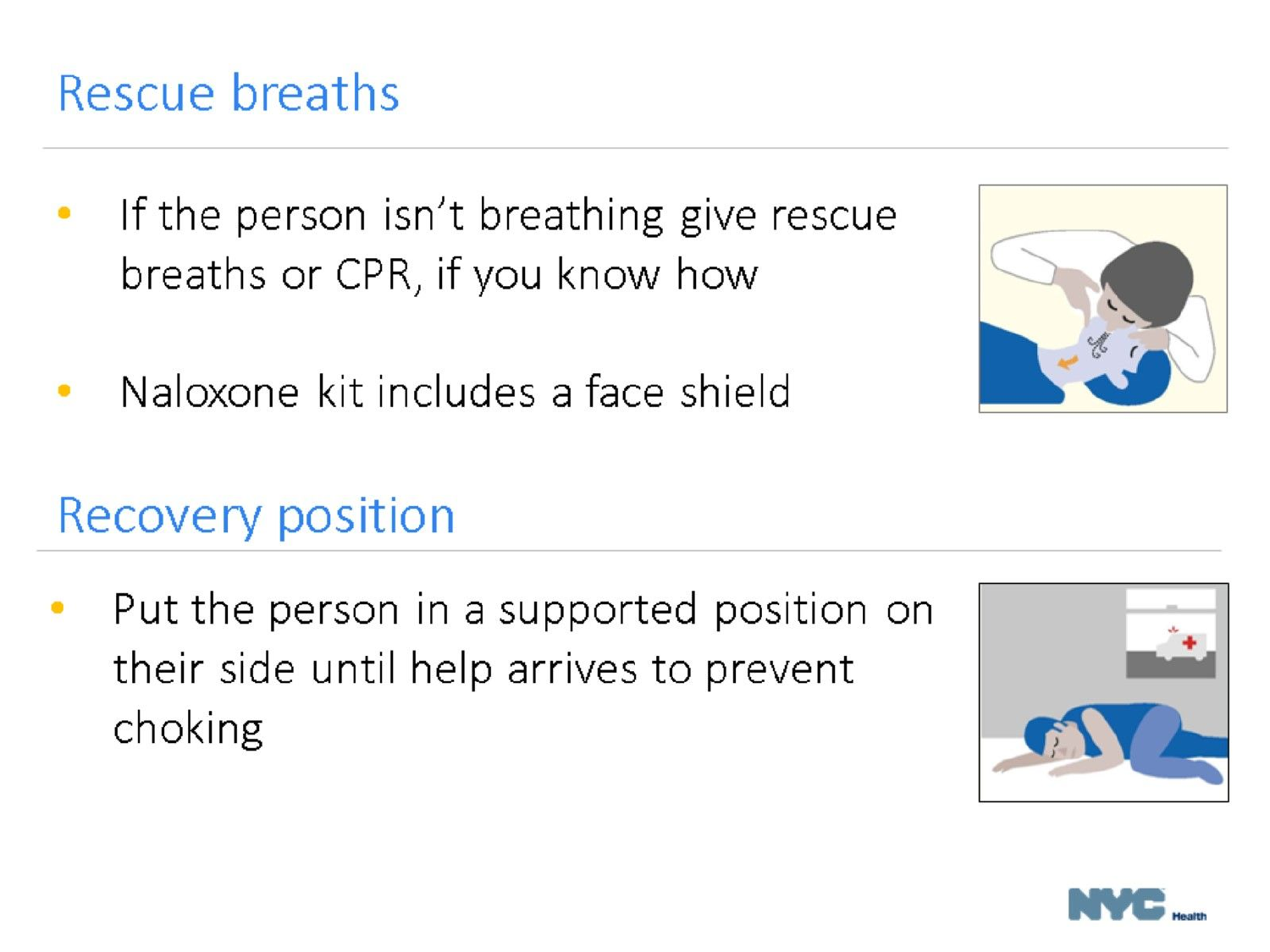

#

## Slide 37
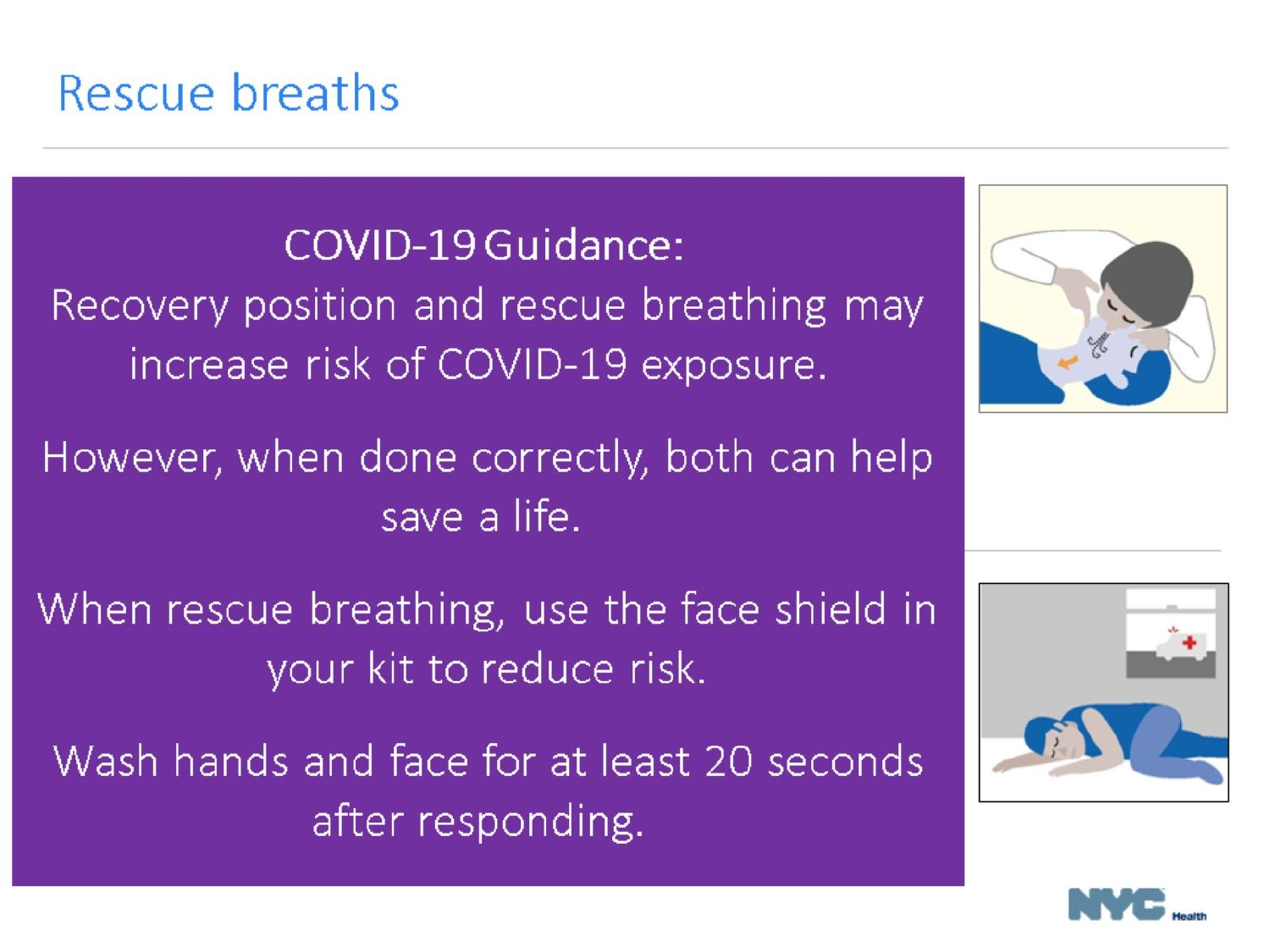

#

## Slide 38
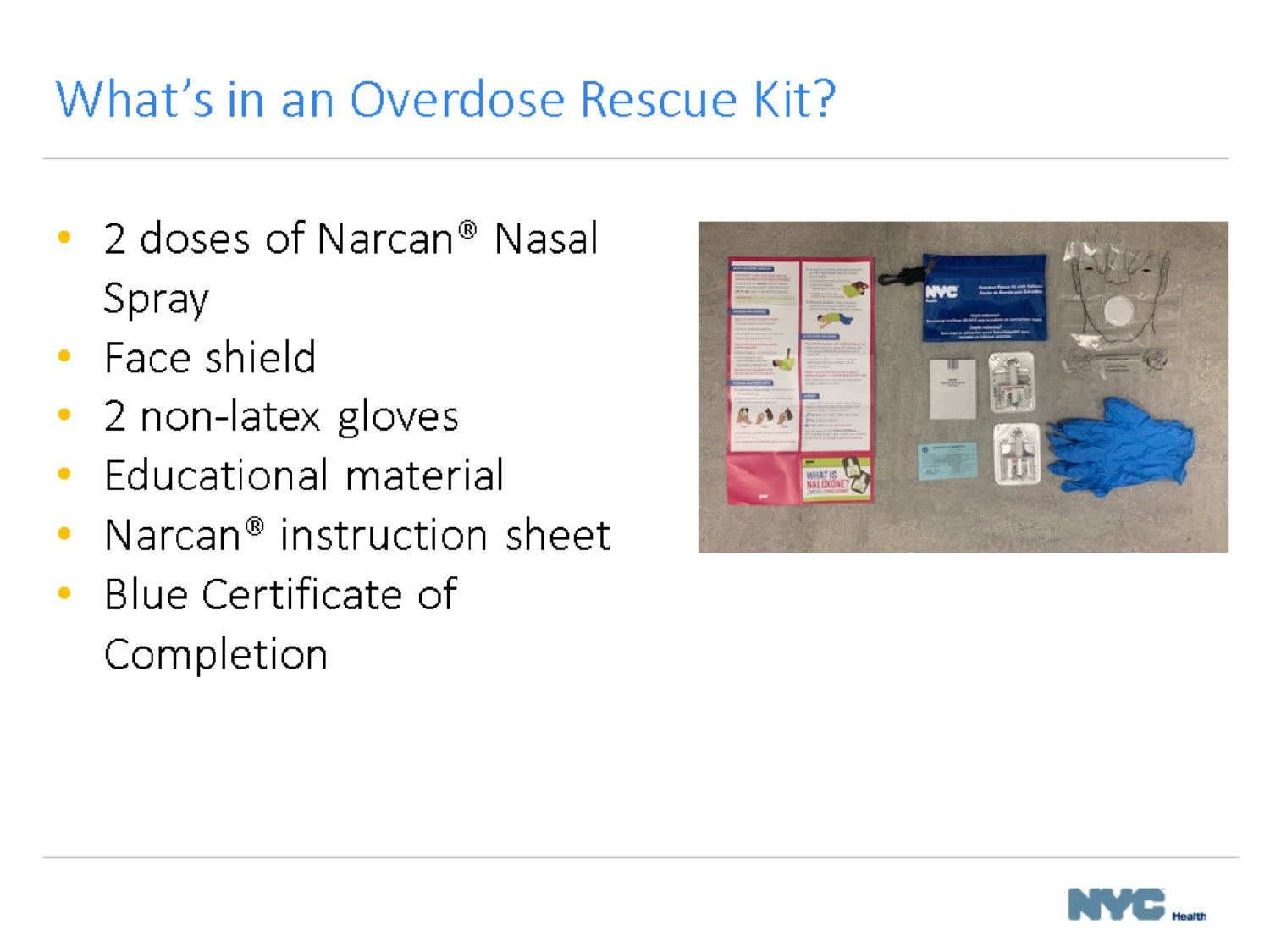

#

## Slide 39
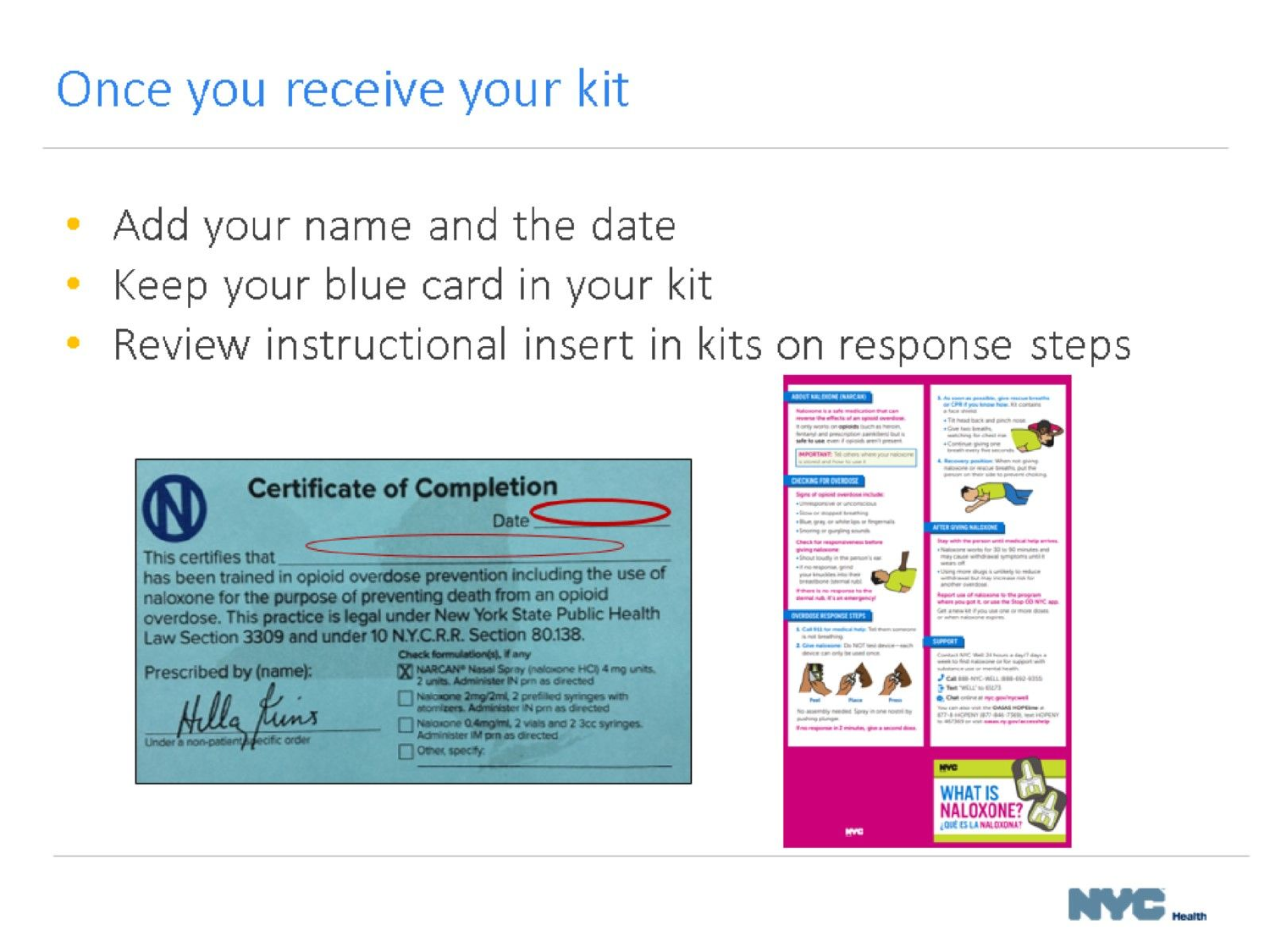

#

## Slide 40
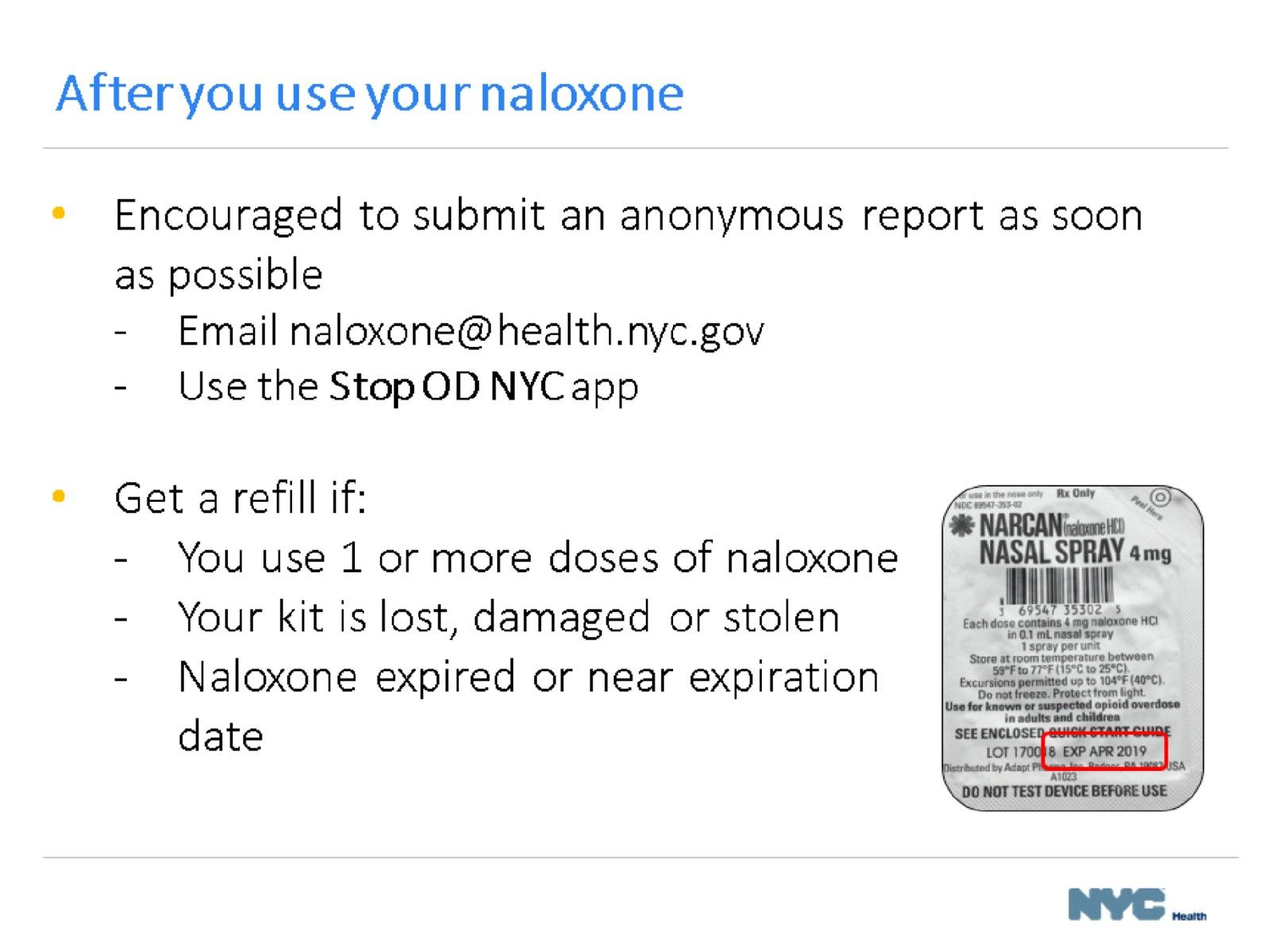

#

## Slide 41
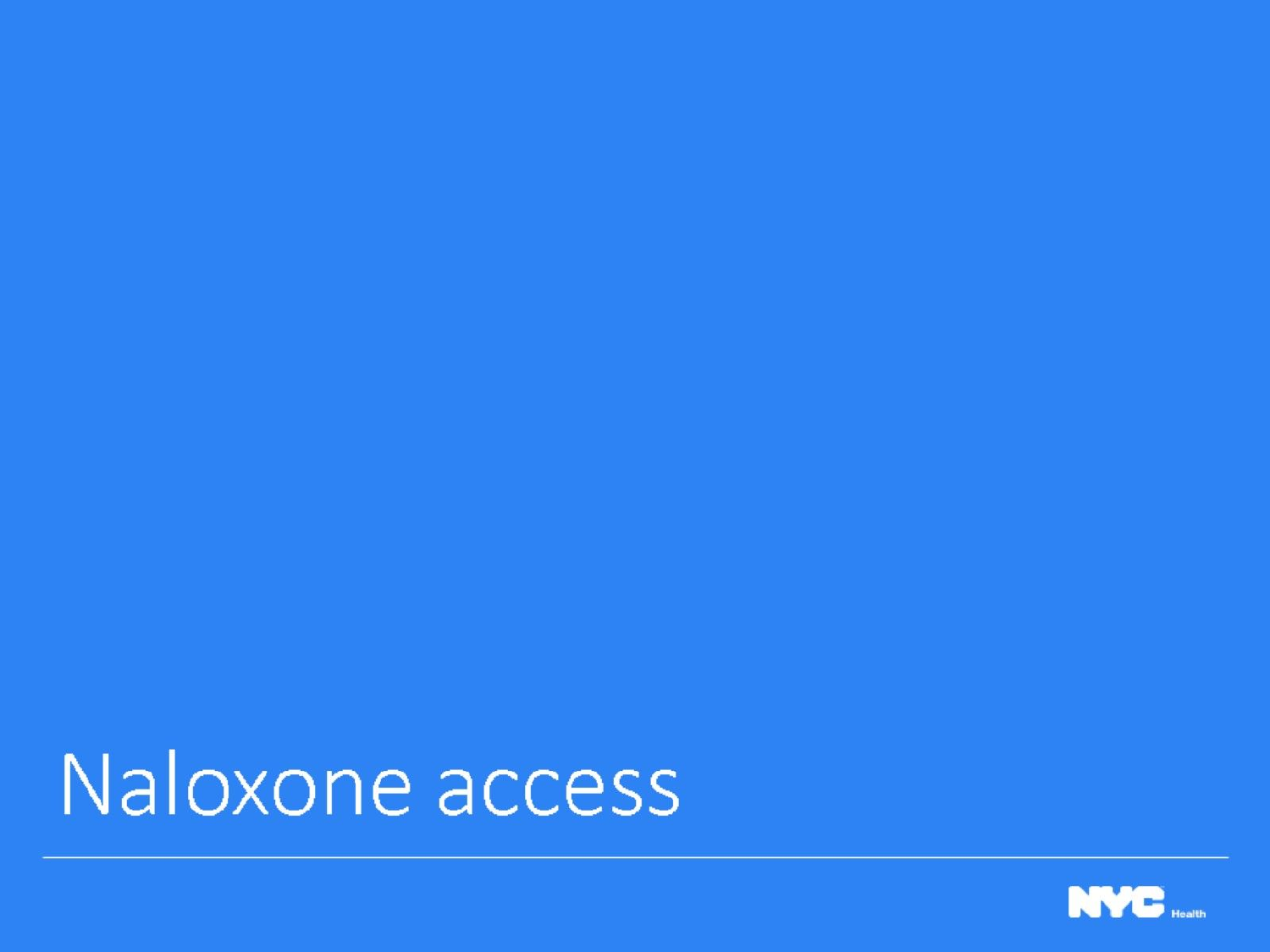

# Naloxone access

## Slide 42
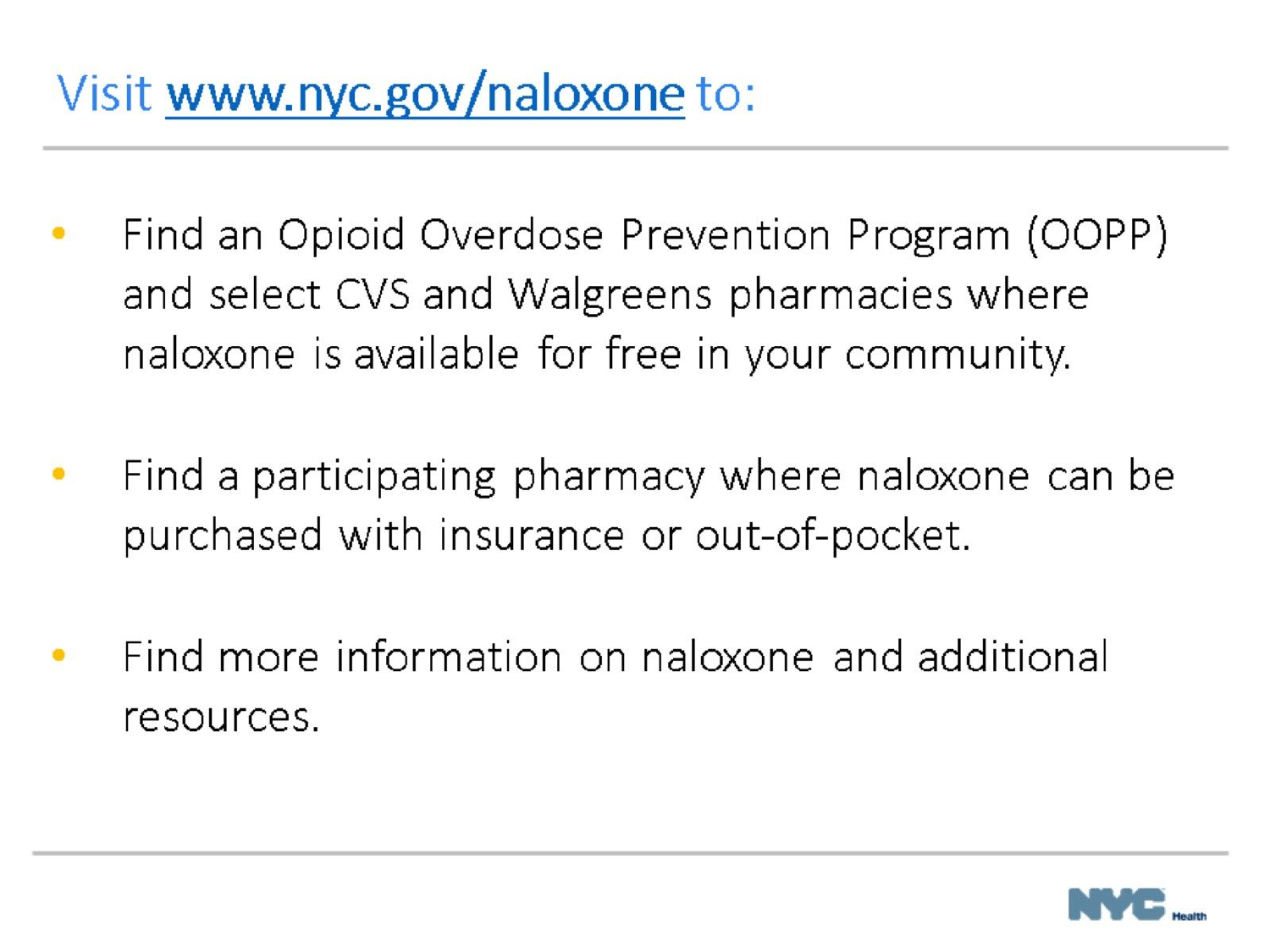

#

## Slide 43
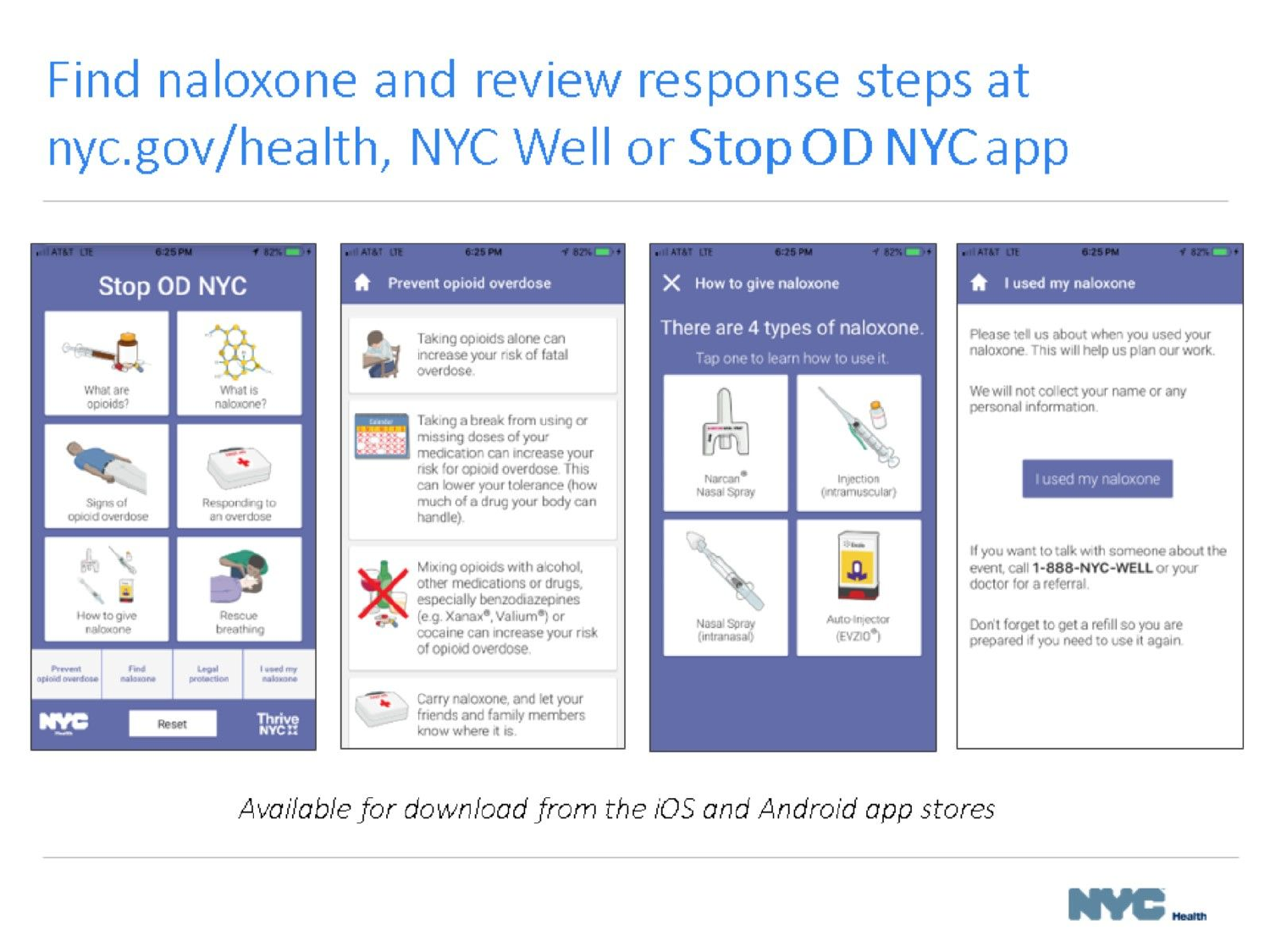

#

## Slide 44
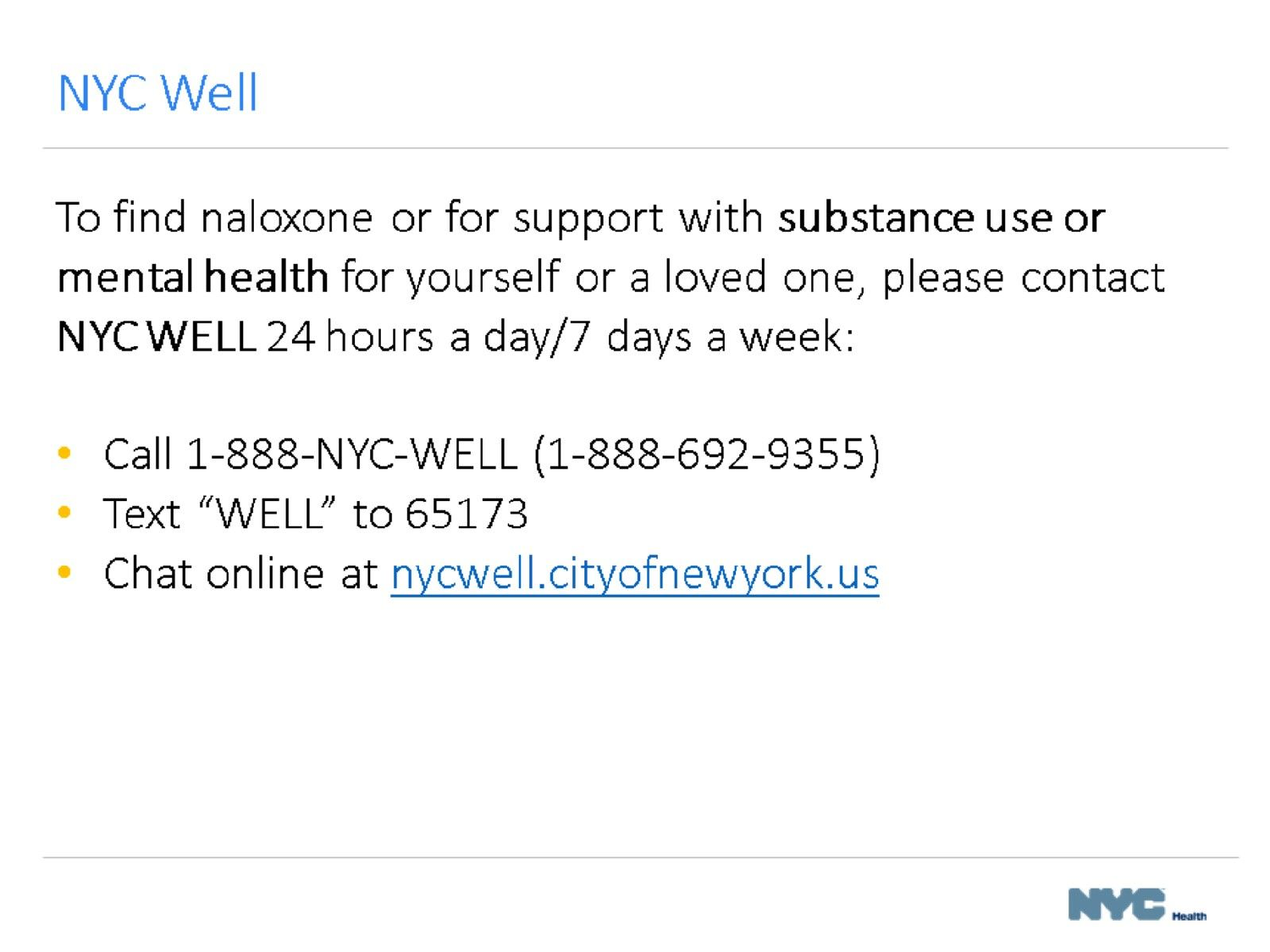

#

## Slide 45
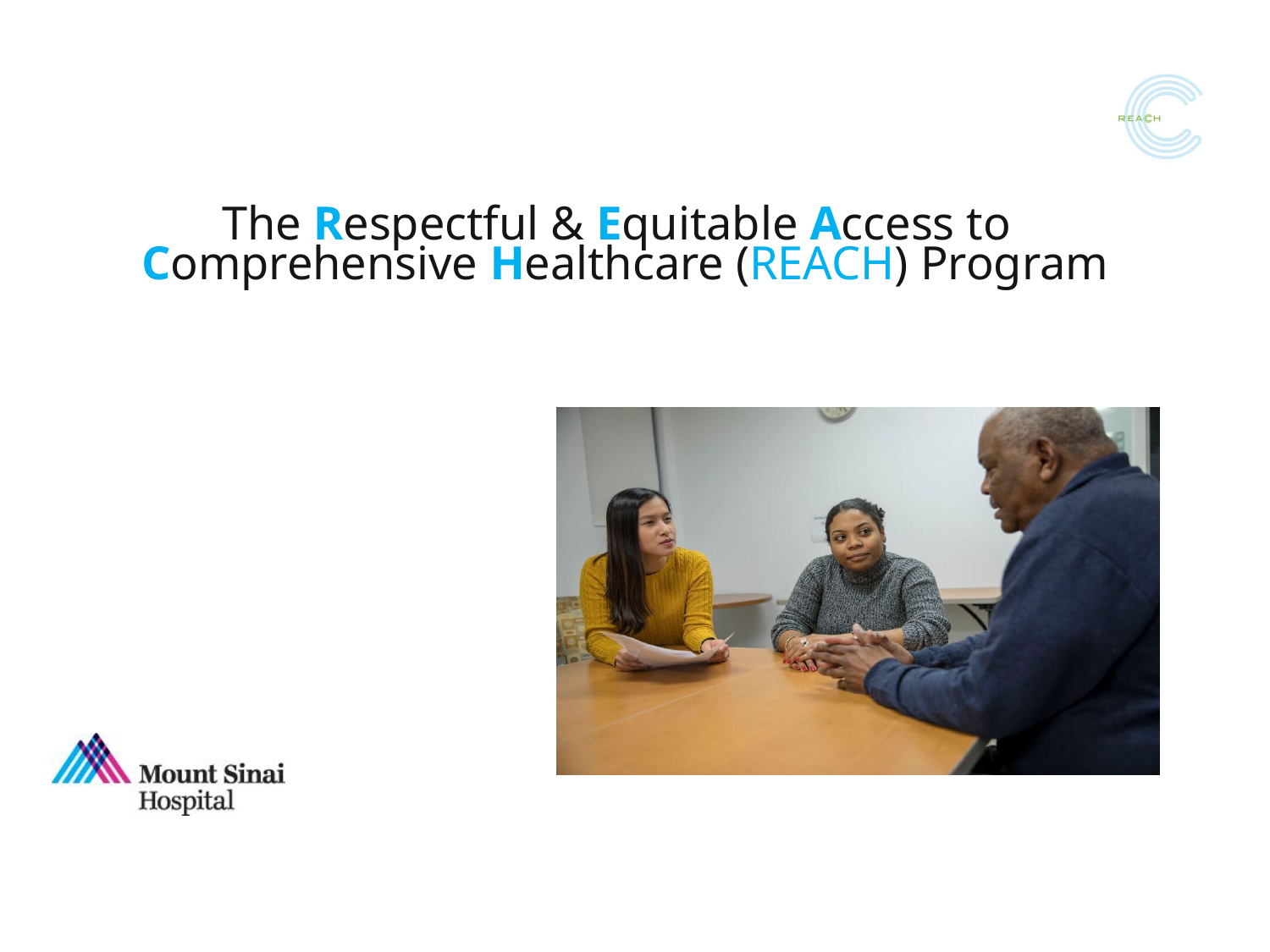

The Respectful & Equitable Access to Comprehensive Healthcare (REACH) Program

## Slide 46
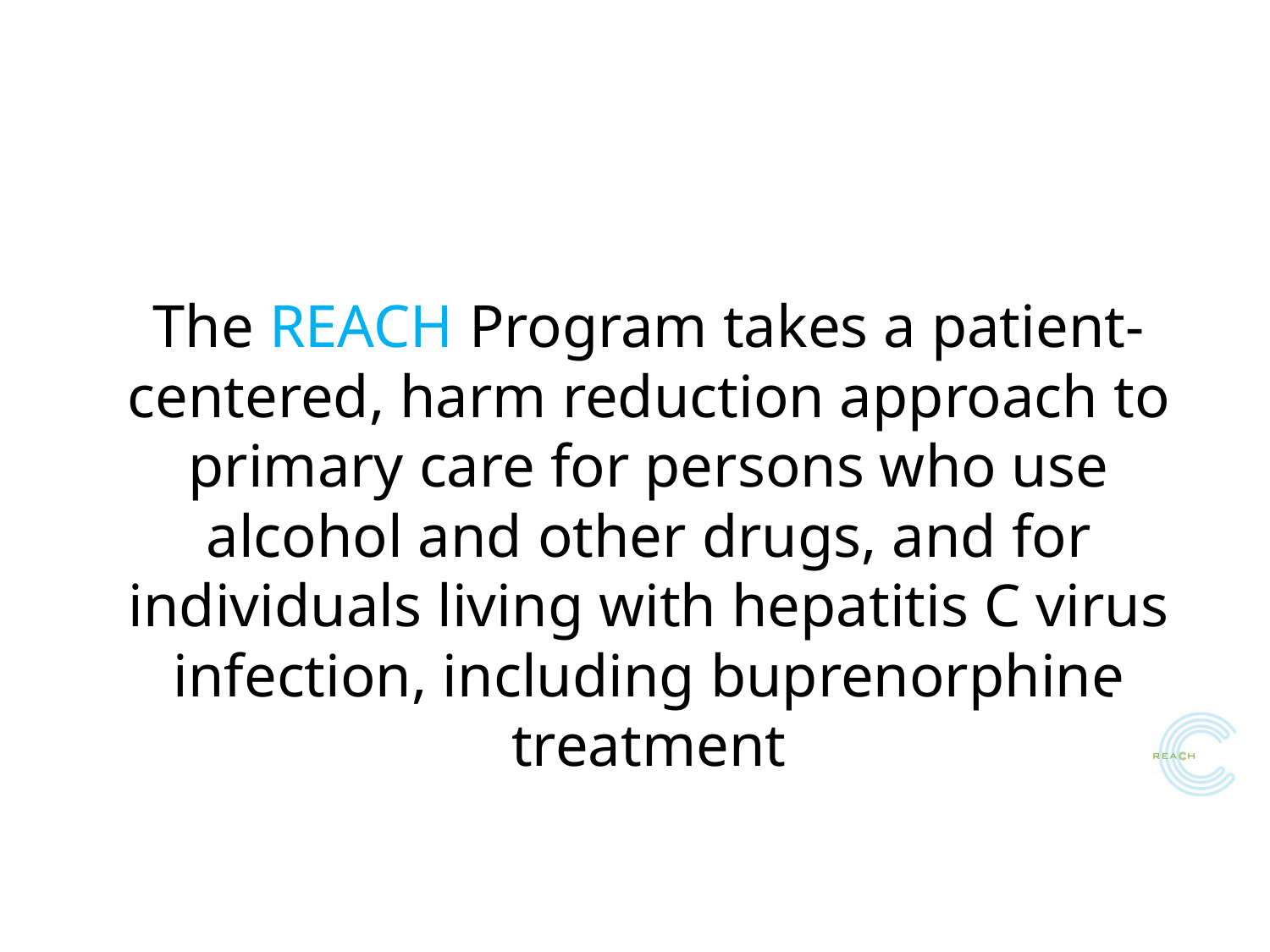

The REACH Program takes a patient-centered, harm reduction approach to primary care for persons who use alcohol and other drugs, and for individuals living with hepatitis C virus infection, including buprenorphine treatment

## Slide 47
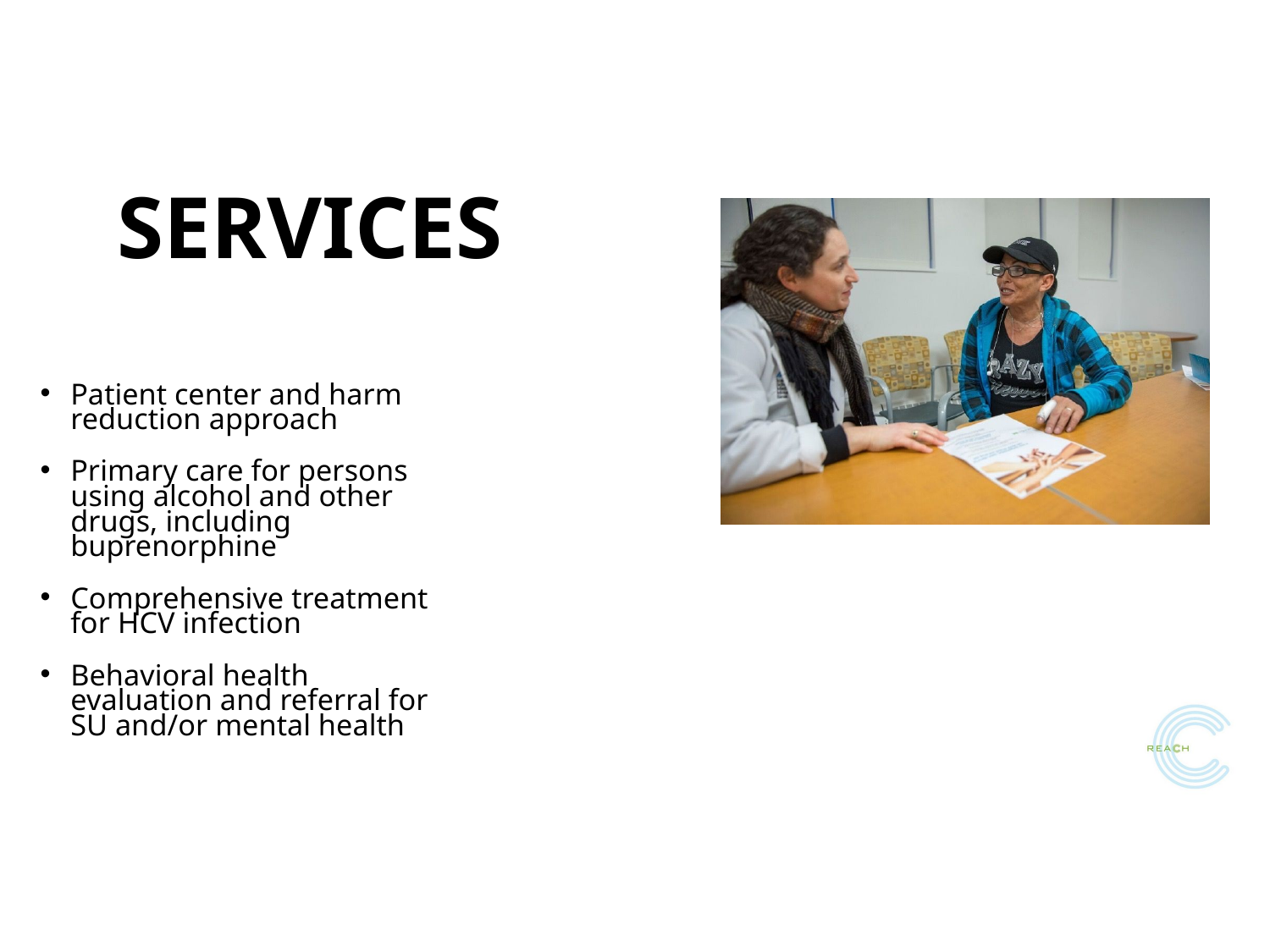

# Services
Patient center and harm reduction approach
Primary care for persons using alcohol and other drugs, including buprenorphine
Comprehensive treatment for HCV infection
Behavioral health evaluation and referral for SU and/or mental health

## Slide 48
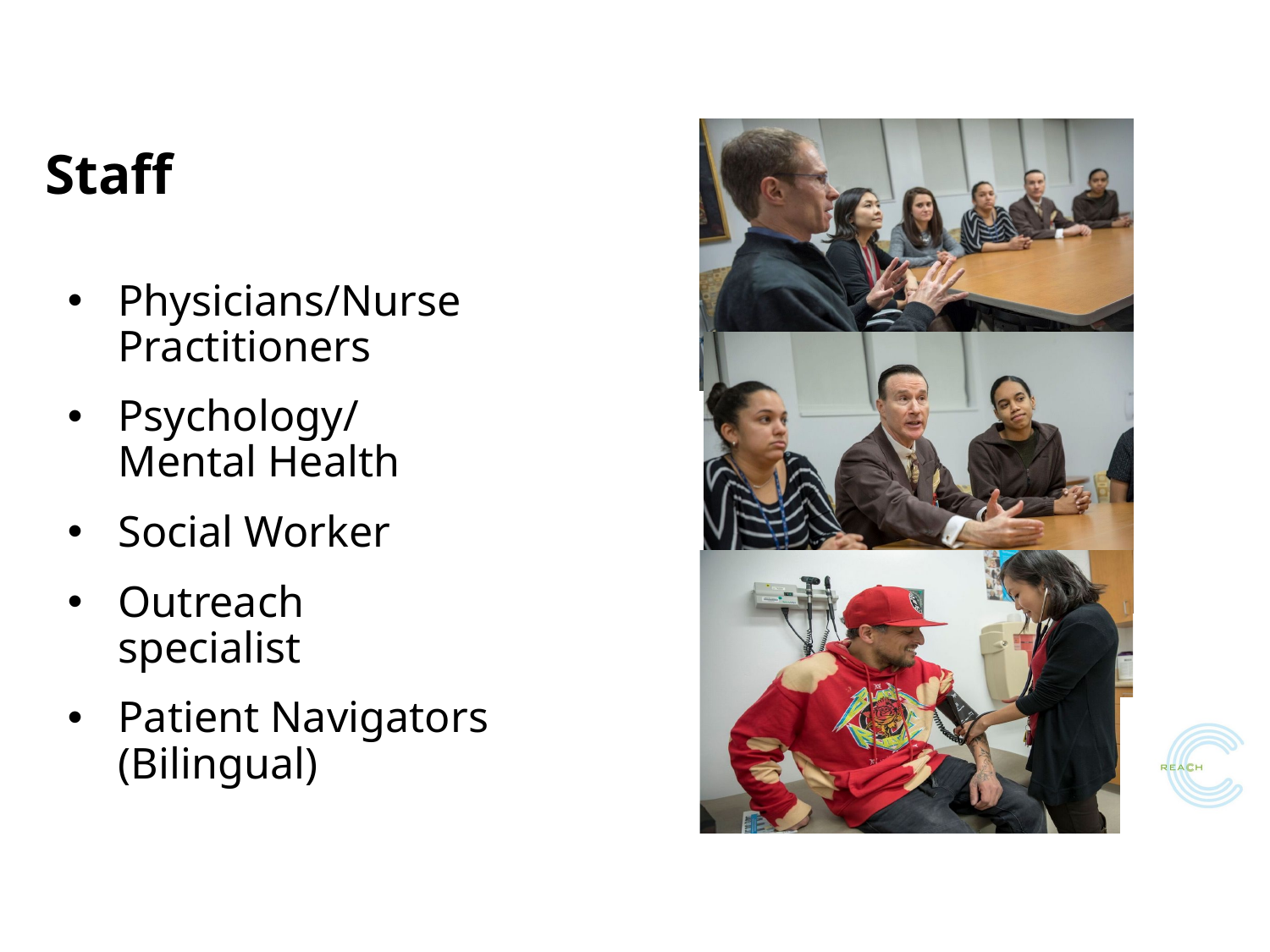

# Staff
Physicians/Nurse Practitioners
Psychology/Mental Health
Social Worker
Outreach specialist
Patient Navigators (Bilingual)

## Slide 49
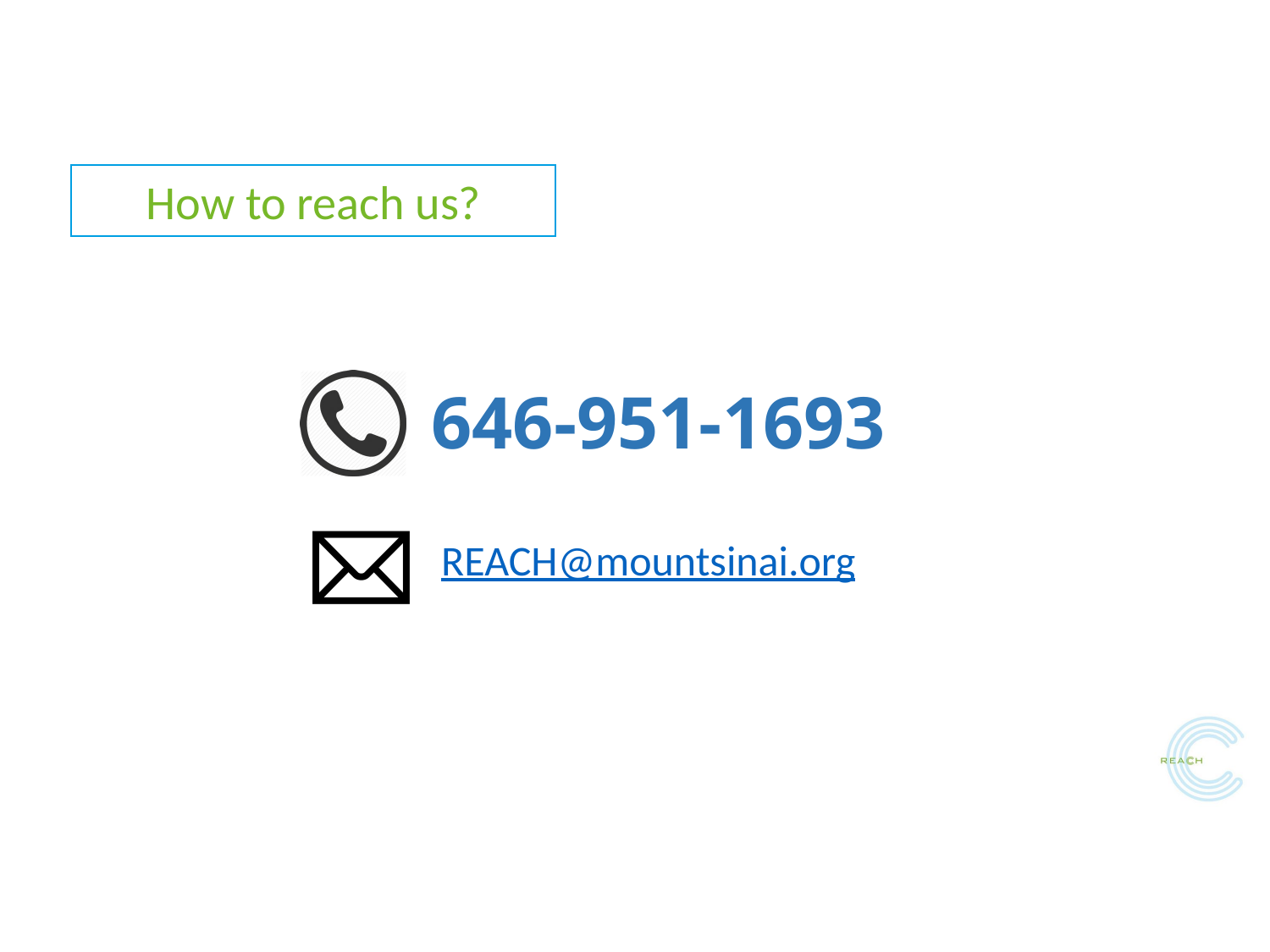

How to reach us?
#
646-951-1693
REACH@mountsinai.org
#REACHWHF19

## Slide 50
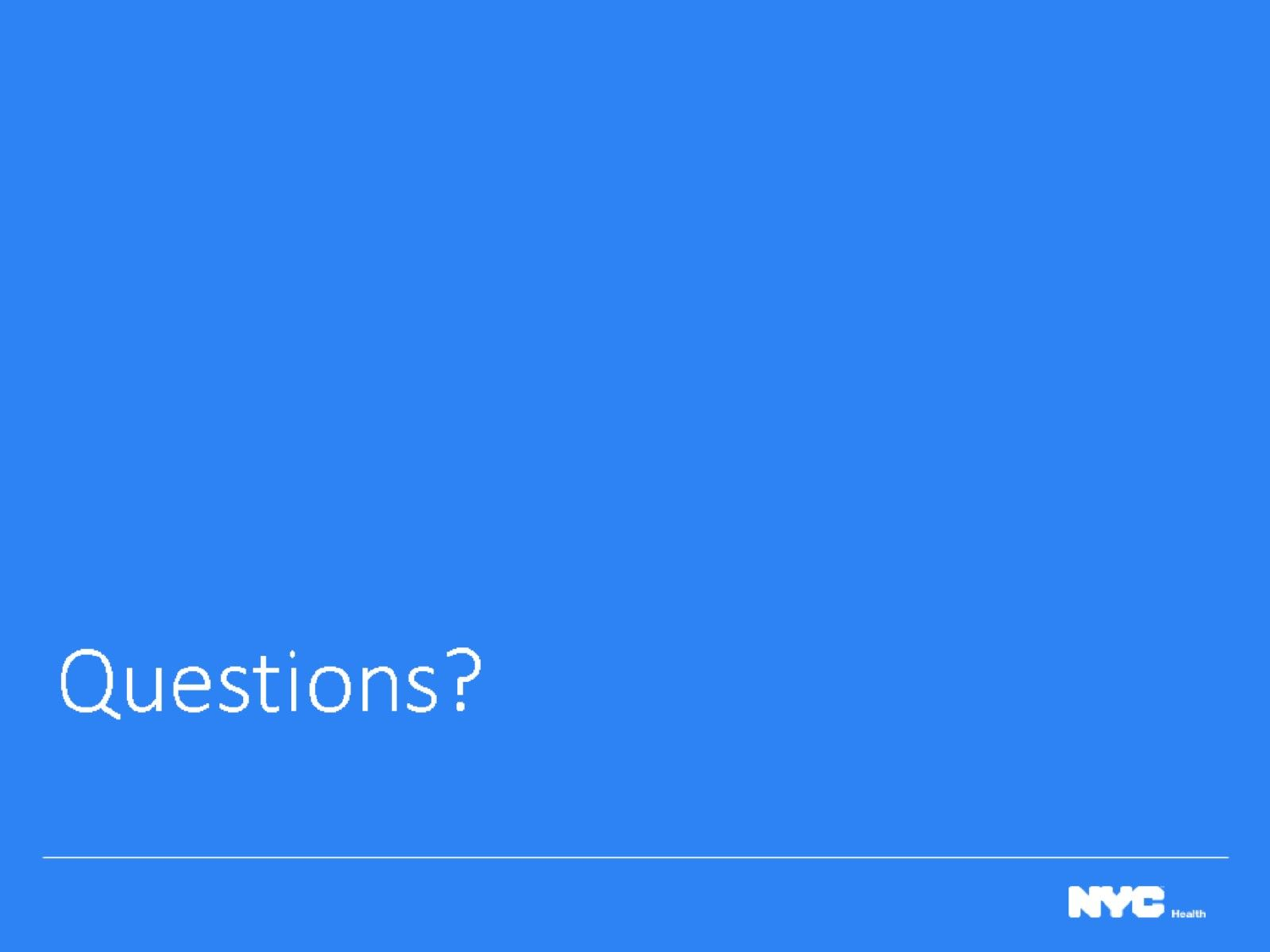

# Questions?
